# Perifollicular stromal cells sustain folliculogenesis via MDK–NCL signaling in mice and humans

**DOI:** 10.1038/s41421-026-00907-3

**Published:** 2026-07-28

**Authors:** Zhe Zhang, Na Kong, Jie Mei, Xiaoqiang Sheng, Jidong Zhou, Haiquan Wang, Nannan Kang, Yang Zhang, Lina Yu, Pengfei Xu, Xin Zhen, Min Wu, Lijun Ding, Guangyi Cao, Dake Li, Chaojun Li, Guijun Yan, Haixiang Sun

**Affiliations:** 1 Department of Reproductive Medicine and Obstetrics and Gynecology, Nanjing Drum Tower Hospital, State Key Laboratory of Pharmaceutical Biotechnology, Nanjing University, Nanjing, Jiangsu China; 2 Nanjing Clinical Medical Center for Reproductive Medicine, Nanjing, Jiangsu China; 3 Center for Molecular Reproductive Medicine, Nanjing University, Nanjing, Jiangsu China; 4 State Key Laboratory of Reproductive Medicine and Offspring Health, Nanjing Medical University, Nanjing, Jiangsu China; 5 Department of Gynecology, Women’s Hospital of Nanjing Medical University, Nanjing, Jiangsu China; 6 Jiangsu Human Reproductive Function Remodeling Engineering Research Center, Nanjing, Jiangsu China

**Keywords:** Cell growth, Senescence, Transcriptomics

## Abstract

Oocyte development requires coordinated metabolic and signaling support from granulosa and theca cells. By performing integrated single-cell RNA sequencing and spatial transcriptomic analyses of murine and human ovaries, we discovered a functionally specialized stromal subtype essential for folliculogenesis. These stromal cells (SCs) with glutamyl aminopeptidase (ENPEP) function, designated perifollicular SCs based on their circumferential follicle localization, exhibit two hallmark features: (1) dynamic proliferation synchronized with follicular maturation from primary to secondary to antral stages, and (2) secretion of midkine (MDK), which activates nucleolin (NCL) receptor signaling to drive granulosa cell (GC) expansion. Furthermore, analyses of ovarian aging revealed the concurrent depletion of perifollicular SCs and the attenuation of MDK–NCL signaling between perifollicular SCs and GCs. The unique spatial confinement and regulatory capacity of perifollicular SCs endow them with the potential to become important components of the follicular functional unit, providing new theoretical support for understanding the molecular regulatory mechanisms of ovarian aging from the perspective of the follicular microenvironment.

## Introduction

The ovarian architecture exhibits a dual-compartment organization comprising the parenchyma and stroma^1,2^. Each follicle, which serves as the fundamental functional unit of the ovarian parenchyma, consists of an oocyte enveloped by concentric somatic cell layers (granulosa and theca cells) that coordinate follicular expansion through paracrine/autocrine regulatory circuits^3^. These follicular structures reside within a stromal microenvironment and display pronounced spatial zonation, featuring dynamic cellular subtypes and region-specific extracellular matrix (ECM) remodeling^4^. Although the ovarian stroma dynamically participates in cellular crosstalk during follicular development and relies on structural and functional complexity, its functional zonation and regulatory mechanisms remain poorly resolved. Defining stromal specialization is critical for understanding reproductive competence and pathologies such as premature ovarian failure.

The ovarian stroma constitutes a dynamic multicellular niche that integrates immune populations, vascular networks, neural projections, and molecularly heterogeneous stromal subtypes within an ECM-rich matrix^5–7^. Functioning as a follicular interface, this compartment orchestrates folliculogenesis through biomechanical constraints and paracrine signaling^8^. Experimental models have demonstrated that three-dimensional culture systems supplemented with defined ECM components^9,10^ and soluble factors (gonadotropins, FGF, and IGF) partially recapitulate oocyte maturation, yet persistent functional disparities persist between in vitro-generated and in vivo-developed follicles^11^. This functional divergence may reveal that perifollicular stromal cells (SCs) actively contribute to follicular development. Single-cell RNA sequencing (scRNA-seq) analyses further reveal spatially partitioned stromal subtypes with discrete developmental trajectories^12,13^, although the current models fail to explain how localized signaling coordinates follicular maturation, partly due to the limited integration of transcriptomic and architectural data.

Through integrated single-cell transcriptomics and spatial profiling, we generated a multimodal developmental atlas spanning five postnatal stages (postnatal days (P) 3, 5, and 7 and weeks (W) 3 and 8) in murine ovaries^14^. Our analysis mapped four molecularly distinct stromal subtypes exhibiting discrete spatial zonation and functional specialization. Mechanistically, we identified a perifollicular SC subtype that orchestrates theca cell–granulosa cell (GC) coordination through midkine signaling (MK signaling) to sustain follicular progression. These findings establish perifollicular SCs as previously unrecognized functional components of the follicular developmental unit, which contributes to the definition of the functional ovarian niche and provides a framework for targeting stromal–GC interactions in individuals with fertility disorders.

## Results

### A single-cell map and spatiotemporal dynamics of gene expression during follicle development in the postnatal ovaries of mice

**Fig. 1:**
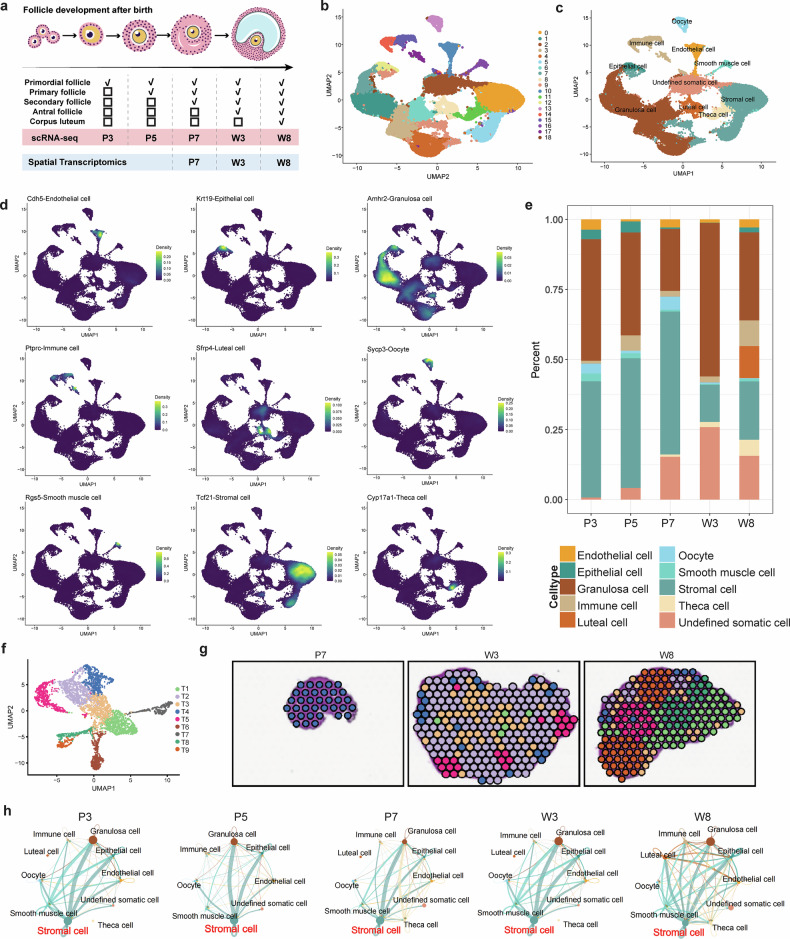
Single-cell map of follicle development in the postnatal ovaries of mice. **a** Temporal and spatial dynamic transcriptome sequencing strategy for mouse ovaries. Ovaries were isolated and disaggregated into single-cell suspensions; cells were barcoded and used for library construction and sequencing using the 10× Genomics platform, and the data produced after sequencing were analyzed with dedicated software (P3, P5, P7, W3, and W8). **b** UMAP plot of ovarian cells colored by 19 clusters. **c** UMAP plot of 10 ovarian cell populations. **d** UMAP plot of cellular markers of different cell populations shown as a function of density. **e** Percentages of the 10 ovarian cell populations at P3, P5, P7, W3, and W8. **f**, **g** UMAP plot and SpatialFeaturePlot of ovarian spots colored by cluster at 10× magnification. **h** Weights/strength of interactions of the 10 ovarian cell populations during the time course from P3 to P5, P7, W3, and W8.

We established a multimodal cellular atlas by integrating single-cell transcriptomic profiles from five developmental stages (P3, P5, P7, W3, and W8) with spatial transcriptomic profiles from three developmental stages (P7, W3, and W8) to elucidate the spatiotemporal dynamic changes in postnatal folliculogenesis (Fig. 1a). This design displays follicular developmental trajectories from primordial to antral stages across the postnatal stage to sexual maturity while systematically mapping niche-specific cellular interactions and enabling the systematic mapping of cellular states across development and their microenvironmental dependencies.

Our integrated single-cell atlas encompassed 58,319 ovarian cells and resolved 19 transcriptionally distinct clusters that were annotated based on canonical marker expression (Fig. 1b). A cluster-specific marker analysis (FindAllMarkers) further classified cells into nine established lineages and one undefined somatic population (Fig. 1c; Supplementary Table S1), with GCs and SCs dominating the cellular proportions (Fig. 1d, e). Spatial transcriptomic analyses (10× Visium) of samples collected at P7, W3, and W8 mapped scRNA-seq-identified lineages onto ovarian niches. The uniform manifold approximation and projection (UMAP) plot revealed nine spatiotemporal clusters (Fig. 1f), with spatial localization patterns revealing a conserved topological organization: endothelial cells, SCs, and GCs formed concentric layers around the oocytes (Fig. 1g; Supplementary Fig. S1a). This resource provides a systematic spatiotemporal annotation of ovarian cellular ecosystems.

The quantitative assessment showed stage-specific variations in interaction frequency and intensity across developmental time points (P3, P5, P7, W3, and W8), with peak activity observed at P3, P7, and W8 (Supplementary Fig. S1b), according to the result of the CellChat analysis. Dynamic intercellular communication patterns during folliculogenesis revealed that SCs predominantly exhibit signaling activity (Fig. 1h). An analysis of different signaling roles indicated that SCs possess distinct functional and structural characteristics compared to other ovarian cell populations (Supplementary Fig. S1c), which suggested that SCs are the most important cell types involved in the transmission of biological signals in the ovary. These findings suggest that SCs function as functional orchestrators of follicular niche dynamics and are essential for coordinating developmental signaling with other cell types in the microenvironment of follicles.

### Identification and genetic dynamics of ovarian SC subtypes

**Fig. 2:**
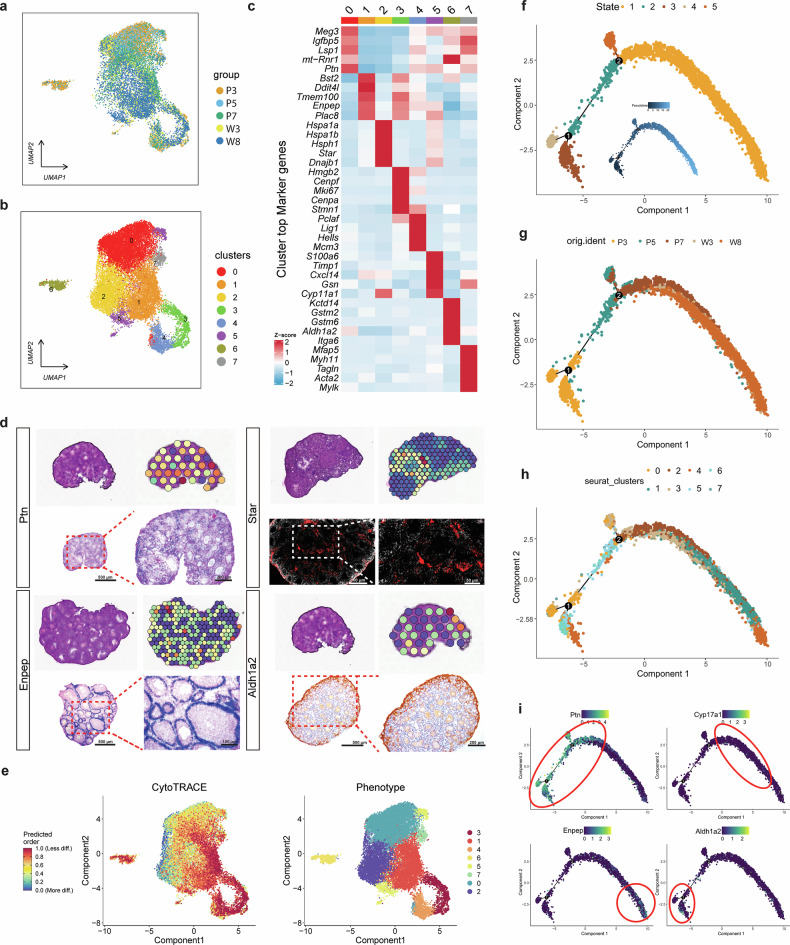
Genetic dynamics of ovarian SC subpopulations. **a** UMAP plot of ovarian SCs colored by time at P3, P5, P7, W3, and W8. **b** UMAP plot of ovarian SCs colored by the 8 clusters. **c** Heatmap of the top 5 marker genes expressed in the SC clusters. **d** The locations of the cellular markers *Ptn*, *Enpep*, STAR, and ALDH1A2 in sections based on the results of the 10× Visium analysis and ISH, IHC, IF staining. **e** CytoTRACE analysis showing the reconstruction of the cellular differentiation trajectories of the 8 clusters. **f** Single-cell trajectories of the 5 SC states identified through a pseudotime analysis as a function of the developmental timeline. **g** Single-cell trajectories of SCs along the development time points (P3, P5, P7, W3, and W8). **h** Single-cell trajectories of SCs along the 8 clusters. **i** Expression of *Ptn*, *Cyp17a1*, *Enpep*, and *Aldh1a2* along single-cell pseudotime trajectories. The red circles highlight cells with high expression of the genes on the pseudotime axis.

Leveraging CellChat-inferred signaling networks, we investigated ovarian SC heterogeneity across five developmental time points. Single-cell sequencing resolved eight transcriptomically distinct stromal subtypes (Fig. 2a, b), with spatially restricted clusters (e.g., Clusters 1, 2, and 6) exhibiting niche-specific distributions (Supplementary Fig. S2a). Cluster-defining markers (*Star*, *Cyp17a1*, *Aldh1a2*, *Gstm2*, *Ptn*, *Col1a1*, *Enpep*, and *Tmem100*) were systematically identified (Fig. 2c; Supplementary Fig. S2c and Table S2), and spatial mapping with the 10× Visium platform combined with orthogonal validation by in situ hybridization (ISH), immunohistochemistry (IHC), and immunofluorescence (IF) staining, confirmed the compartmentalized expression of *Ptn* mRNA*, Enpep* mRNA, STAR protein, and ALDH1A2 protein (Fig. 2d). Functional zonation emerged: *Enpep* mRNA marked perifollicular stromal niches correlated with follicular growth; STAR protein localized to both theca-adjacent and distal stromal domains; and ALDH1A2 protein dominated the cortical stroma and epithelial interfaces. These findings establish ovarian SCs as molecularly and spatially stratified subtypes that are classifiable through integrated single-cell and spatial omics analyses.

We integrated the CytoTRACE-predicted differentiation potential with marker expression to determine the SC fate during folliculogenesis and identified Cluster 6 as putative less differentiated SCs (Fig. 2e). Pseudotemporal ordering via Monocle2 revealed five differentiation states and two bifurcation points (Fig. 2f, g). Early postnatal stages (P3) predominantly occupied root states, with stromal diversification initiating at P7 — a critical window coinciding with secondary follicle expansion (Fig. 2h). The trajectory analysis positioned Cluster 6 and a subtype of cells in Cluster 4 at differentiation origins. The stage-resolved expression patterns of *Ptn*, *Cyp17a1*, *Enpep*, and *Aldh1a2* (Fig. 2i) further supported the partitioning of SCs into four functionally distinct subtypes defined by differentiation trajectories and the expression of effector gene modules.

### SC classification and functional analysis-based pseudotime trajectories

**Fig. 3:**
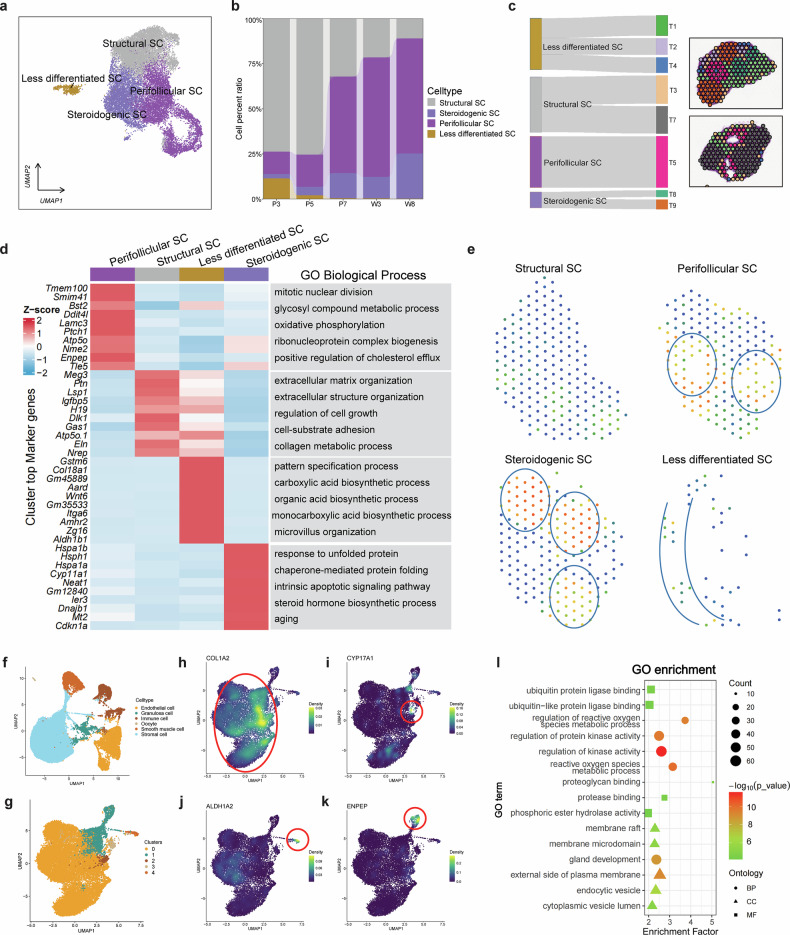
Identification of ovarian SC subtypes. **a** UMAP plot of 4 specific SC subtypes in mouse ovaries. **b** Percentages of the 4 SC subtypes at P3, P5, P7, W3 and W8. **c** MIA of the specific ovarian SC subtypes mapping to ovarian tissue. **d** Heatmap of the top 5 marker genes expressed in the 4 SC subtypes and GO analysis of enriched biological processes. **e** Spatial localization of 4 SC subtypes on slices analyzed using 10× Visium and an RCTD analysis. **f** UMAP plot of 6 ovarian cell populations in the human ovary scRNA-seq dataset (GSE255690); colors indicate the different populations. **g** UMAP plot of SCs colored by the 5 clusters identified in the human ovary scRNA-seq data. **h**–**k** UMAP plots of the cellular markers (*COL1A2*, *CYP17A1*, *ALDH1A2*, and *ENPEP*) of 4 SC subtypes in human ovaries shown as a function of density. Red circles highlight cells with high expression of the indicated genes in different SC clusters in human ovaries. **l** GO analysis of differentially expressed genes in perifollicular SCs in human ovaries.

Integrating multimodal data, we defined four ovarian stromal subtypes — structural SCs (extracellular matrix organization), perifollicular SCs (proliferative niches), less differentiated SCs (differentiation potential), and steroidogenic SCs (hormone synthesis) (Fig. 3a), and UMAP plots of the results obtained at different postnatal stages (P3–W8) are presented in Fig. 3a and Supplementary Fig. S2b. Dynamic shifts in abundance revealed stage-dependent subtype predominance — the abundances of structural SCs and less differentiated SCs decreased postnatally, whereas the abundances of prefollicular SCs and steroidogenic SCs increased after P7 (Fig. 3b). Multimodal intersection analysis (MIA) validated the spatial context of the stromal classification (Fig. 3c). The Gene Ontology (GO) enrichment analysis of subtype-specific markers further resolved the functional divergence (Fig. 3d): Structural SCs were enriched for ECM organization and regulation of cell growth (*Ptn*^15^ and *Col1a2*^16–18^); Perifollicular SCs for mitotic nuclear division and oxidative phosphorylation (*Teme100*^19^ and *Enpep*^20^); Steroidogenic SCs for steroid hormone biosynthetic process and aging (*Star* and *Cyp17a1*^21^); Less differentiated SCs for pattern specification process and microvillus organization (*Gstm2*^22^ and *Aldh1b1*^23^).

We mapped transcription factor (TF) regulon activity via SCENIC to elucidate the transcriptional regulatory mechanism underlying the functions of SC subtypes and identified dynamic TF networks orchestrating folliculogenesis. Leveraging 51 regulon activities (5481 target genes), we identified stage-resolved regulon activation patterns, with key developmental regulators (*Maf*, *Wt1*, *Maff*, *Bclaf1*, and *Stat1*) showing subtype- and stage-specific activity (Supplementary Fig. S2d). Robust Cell Type Decomposition (RCTD)-based spatial mapping demonstrated subtype-specific niche localization, with functional enrichment patterns mirroring their topological distributions (Fig. 3e). These spatially and functionally resolved stromal subtypes redefine ovarian niche organization, linking cellular identity to the regulation of folliculogenesis.

We validated the evolutionary conservation of the ovarian SC classification by analyzing cynomolgus monkey (GSE130664)^24^ and human (GSE255690)^2^ ovarian datasets. Monkey SCs were resolved into four conserved clusters (Supplementary Fig. S2e, f), with the *COL1A2*^+^ and *TMEM100*^+^ subtypes indicating spatially distinct niches in the dimensionality-reduced clusters (Supplementary Fig. S2g, h). In human ovaries, integrated transcriptomics classified SCs into five clusters (Fig. 3f, g). Cross-species alignment using mouse stromal markers (*COL1A2*, *CYP17A1*, *ALDH1A2*, and *ENPEP*; Fig. 3h–k) revealed a conserved stromal zonation: Human *ENPEP*^+^ SCs (Cluster 1) exhibited functional congruence with murine perifollicular SCs and were enriched in the regulation of kinase activity (GO:0043549) and reactive oxygen species metabolic processes (GO:0072593) according to the results of the GO enrichment analysis, as shown in Fig. 3l. These results establish the biological similarity of stromal niche organization across different mammals, linking molecular signals to the regulation of folliculogenesis.

### Specific regulon networks of ovarian SCs during follicle development

**Fig. 4:**
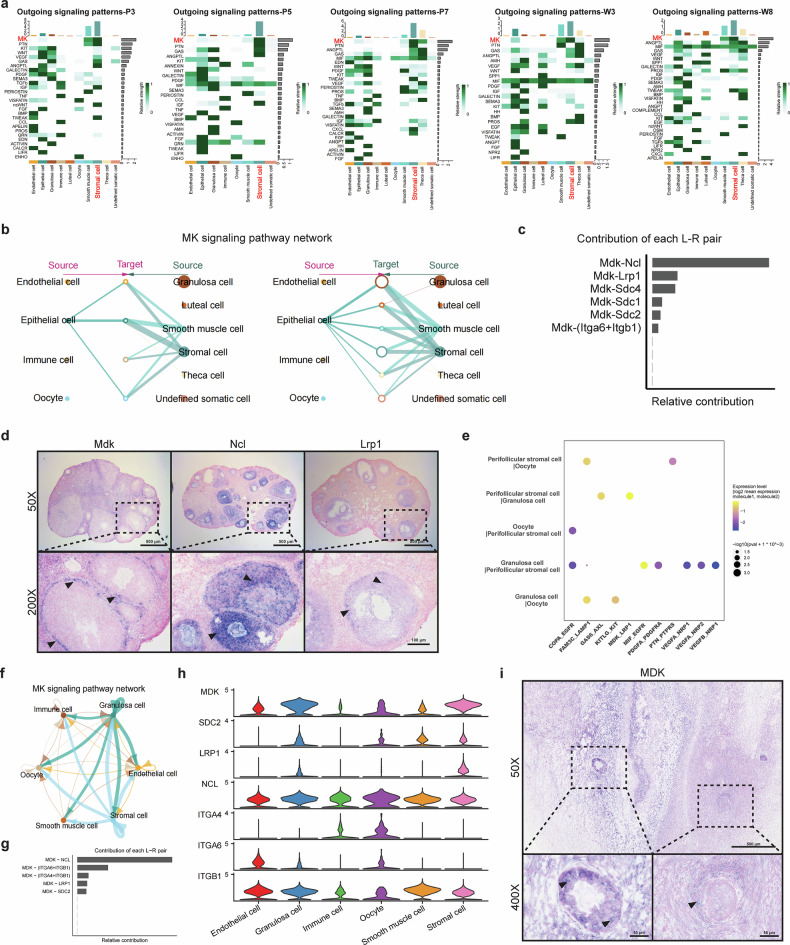
Stromal-derived MDK co-culture promotes follicle development. **a** The outgoing signaling patterns of the 10 ovarian cell populations at P3, P5, P7, W3, and W8. **b** MK signaling pathway network among the 10 ovarian cell populations. **c** The contribution of each L−R pair to the MK signaling pathway. **d** Representative images showing the location and RNA expression levels of *Mdk*, *Ncl* and *Lrp1* by ISH in ovarian sections from W8 mice. Arrowheads indicate positive signals in ovaries. **e** The intensity of signaling pathways related to follicular development among perifollicular SCs, GCs, and oocytes in the follicular microenvironment. **f** The weights/strength of interactions of the 6 ovarian cell populations in the human ovary. **g** The contribution of each L−R pair to the MK signaling pathway in the human ovary. **h** Violin plot showing the expression profiles of ligands and receptors in the MK pathway in six ovarian cell populations. **i** Representative images showing the location and RNA expression levels of *MDK* detected using ISH in a human ovarian section. Arrowheads indicate positive signals in ovaries.

Our analysis revealed pervasive SC communication across all five developmental stages, with SCs engaging in frequent signaling crosstalk with neighboring cell subtypes. Stage-resolved ligand–receptor mapping revealed seven dominant stromal-derived pathways (PTN, MK^15^, noncanonical WNT^25^, PROS^26^, GAS^27^, ANGPTL^28^, and TWEAK^29^) that orchestrate folliculogenesis (Fig. 4a). MK signaling persisted throughout folliculogenesis, while PTN activity was restricted to pre-pubertal stages (P3–W3). A comparative pathway analysis revealed the following stage-specific regulatory modules (Supplementary Fig. S3a): P3 (nWNT, PROS, APELIN, GRN, and CALCR), P5 (IL-1, ANNEXIN, CCL, BMP, and FGF), P7 (TWEAK, APELIN, ANGPT, CALCR, and CXCL), W3 (NPR2, EGF, HH, BMP, and AMH), W8 (LIFR, FGF, KIT, SPP1, and MIF).

Notably, stromal-derived MK signaling spanned all developmental windows, suggesting its role as a constitutive niche maintenance signal. The spatiotemporal regulation of the PTN/MK (early) to TWEAK/ANGPT (mid) to NPR2/HH (late) pathways delineates a stage-specific stromal signaling code. These dynamic secretory programs position SCs as central signaling hubs that coordinate follicular developmental transitions through temporally constrained paracrine modules.

MK signaling exhibited sustained activity throughout folliculogenesis (Fig. 4b), with SCs as primary midkine (MDK) protein sources and GCs as dominant targets. The analysis of receptor–ligand pairs identified *Ncl*, *Lrp1*, *Sdc4*, and *Sdc1* as core components of the MK pathway (Fig. 4c). Spatial mapping of *Mdk* mRNA via ISH revealed that stromal-enriched expression peaked at P5, with perifollicular localization diminishing around the antral follicles and corpora lutea (Supplementary Fig. S3b), consistent with stage-specific roles in follicular maturation. In mature follicles (W8), *Ncl* and *Lrp1* localized predominantly to GCs (Fig. 4d). Single-cell ligand–receptor mapping confirmed that *Mdk* is involved in the strongest SC–GC signaling axis, which is complemented by bidirectional MIF-mediated GC–SC crosstalk (Fig. 4e). The cross-species analysis revealed conserved MK pathway activity in human ovaries, although with expanded cellular sources — both GCs and SCs emitted MK signals (Fig. 4f–h). ISH using a human *MDK* probe showed developmental stage-dependent differences in localization between GCs and SCs, potentially reflecting species-specific niche adaptations. These results establish MK signaling as an evolutionarily conserved SC‒GC communication axis that is dynamically regulated across follicular transitions. While human inter-individual variability necessitates further validation, our multi-species framework positions MK signaling as a central regulator of follicular niche homeostasis.

### Spatiotemporal dynamics of GCs cooperate with SC subtypes to regulate folliculogenesis

**Fig. 5:**
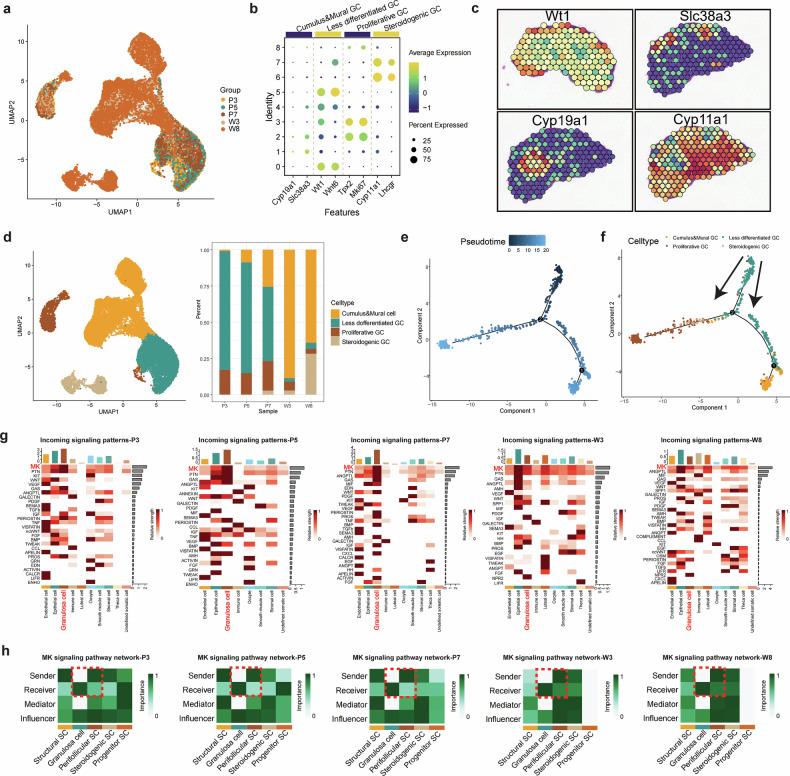
Genetic dynamic signatures of the GC lineage during follicle development. **a** UMAP plot of GCs colored by analysis at P3, P5, P7, W3, and W8. **b** Dot plot of special cellular markers of GCs in mice. **c** SpatialFeaturePlot of ovarian spots colored by the expression of special cellular markers of GCs at 10× magnification. **d** UMAP plot of 4 GC subtypes and percentages of the 4 GC subtypes at P3, P5, P7, W3, and W8. **e** Single-cell trajectories of the GC subtypes as a function of the developmental timeline and cell state. **f** Single-cell trajectories of SC subtypes along with the 4 GC cell types. **g** The incoming signaling patterns of the 10 ovarian cell types at P3, P5, P7, W3, and W8. **h** Comparison of the cellular roles between GCs and 4 SC subtypes in the MK signaling pathway at P3, P5, P7, W3, and W8. The red dashed rectangles highlight the roles played by GCs and perifollicular SCs in the MK signaling pathway at P3, P5, P7, W3, and W8.

Our findings established SC-derived MK signaling as a critical regulator of follicular development through GC modulation. Given the spatiotemporal dynamics of SC subtypes, we asked whether GCs exhibit complementary heterogeneity to coordinate niche functions^30^. UMAP-based clustering resolved four GC subtypes across developmental stages: differentiated cumulus/mural GCs (*Cyp19a1* and *Slc38a3*), less-differentiated GCs (*Wt1* and *Wnt6*), proliferative GCs (*Tpx2* and *Mki67*), and steroidogenic GCs (*Cyp11a1* and *Lhcgr*) (Fig. 5a, b; Supplementary Table S3). Spatial transcriptomics confirmed the histological zonation of these subtypes, with less differentiated GCs localized to primordial follicle niches and steroidogenic GCs enriched in antral follicles (Fig. 5c). Proportional shifts revealed developmental priming: the abundance of less differentiated GCs progressively decreased from P3 to W3, whereas the abundance of steroidogenic GCs increased after P7 (Fig. 5d). Pseudotemporal ordering revealed progressive differentiation from less differentiated (trajectory root) to steroidogenic states (Fig. 5e, f), mirroring follicular maturation. These spatiotemporally resolved GC states synergize with stromal dynamics to coordinate folliculogenesis.

We elucidated the cellular origin of MK signaling targeting GCs by prioritizing ligands and receptors across SC subtypes. MK emerged as the dominant incoming signaling pathway in GCs throughout folliculogenesis (Fig. 5g), with perifollicular SCs identified as the primary MDK protein source through a spatiotemporal signaling network analysis (Fig. 5h). These results establish perifollicular SC-derived MK signaling as a dynamically regulated paracrine axis that is essential for follicular niche maturation, where stage-specific MDK secretion might coordinate GC differentiation and proliferation while maintaining stromal ecosystem connectivity.

### Stromal-derived MDK co-culture promotes follicle development

**Fig. 6:**
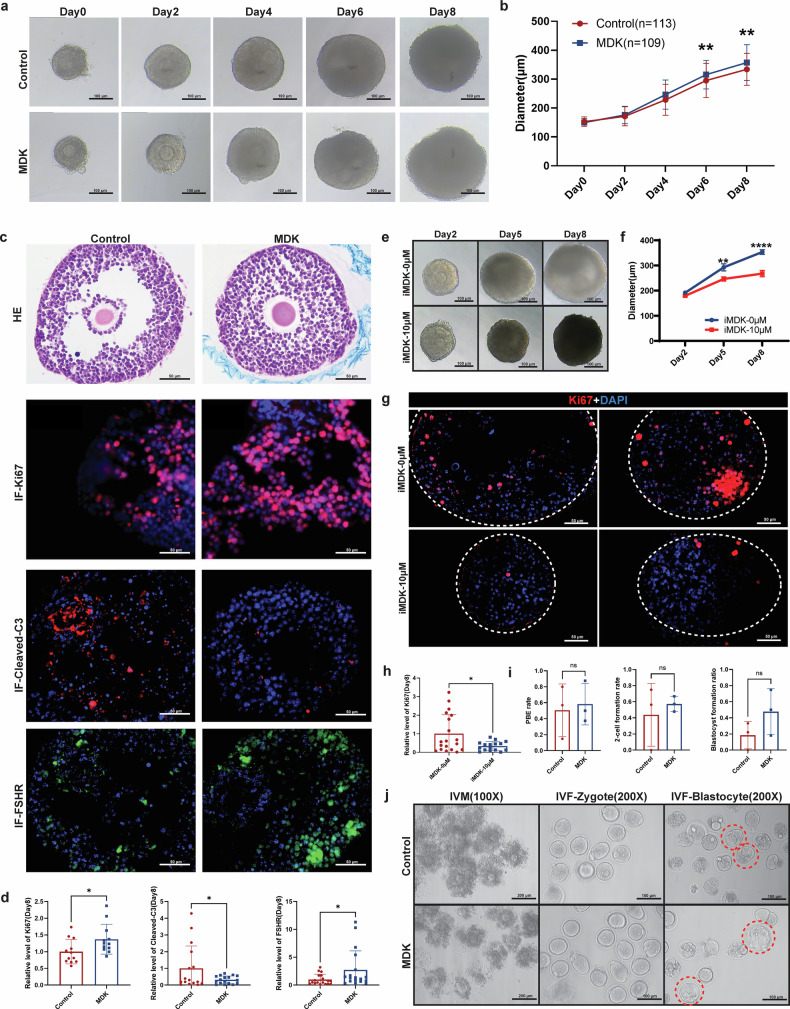
Stromal-derived MDK co-culture promotes follicle development. **a** Analysis of secondary follicles after culture in the alginate system in vitro with/without the addition of 1 mg/L MDK. **b** Analysis of the diameter of the follicles in 8-day cultures (^**^P < 0.01, Student’s *t*-test). **c** Hematoxylin‒eosin (HE) staining was performed to determine the histological structure inside the follicle after culture in vitro on Day 8, and IF staining was performed to determine the proliferation level (Ki67), apoptosis level (cleaved C3), and hormone response (FSHR) of the GCs in the follicle cultures in vitro on Day 8. **d** Relative expression levels of Ki67, cleaved C3, and FSHR detected by IF staining on Day 8. Follicles were fixed with a special 4% PFA fixative containing calcium ions and embedded after dehydration in a gradient of ethanol solutions. Three follicles from each sample were embedded (^*^P < 0.05, Student’s *t*-test). **e** The secondary follicle was divided for culture in the alginate system in vitro with/without the addition of 10 μM iMDK. **f** The diameter of the follicles was analyzed after 8 days of culture (^**^P < 0.01 and ^****^P < 0.0001, Student’s *t*-test). **g** IF staining was performed to de*t*ermine the proliferation level (Ki67) after the addition of iMDK to the follicles cultured in vitro on Day 8. The white dashed circles emphasize the localization of the follicles. **h** Relative expression levels of Ki67 determined by IF staining on Day 8. Three follicles from each sample were embedded (*P < 0.05, Student’s *t*-test). **i** Polar body extrusion (PBE) rate, 2-cell formation rate and blastocyst formation rate obtained after the IVM and IVF of follicles cultured in vitro. **j** Mature oocytes obtained with or without MDK supplementation were subjected to IVM and IVF and further cultured to obtain blastocysts. The red dashed circles emphasize the localization of the follicles.

Functional validation of perifollicular stromal-derived MDK through exogenous supplementation (1 mg/L) in alginate-encapsulated secondary follicles revealed stage-specific developmental regulation (Fig. 6a)^31^. MDK enhanced follicular growth kinetics on Days 6 and 8 (Day 6: control treatment, *n* = 113, 295.3 + 59.15 μm vs MDK treatment, *n* = 109, 315.4 + 49.67 μm, *P* = 0.0048; Day 8: control treatment, *n* = 113, 325.8 + 61.29 μm vs MDK treatment, *n* = 109, 347.1 + 58.41 μm, *P* = 0.0086), accelerating the increase in diameter despite the equivalent terminal size threshold (400 μm) (Fig. 6b). As shown in Fig. 6c, d, treated follicles displayed the activation of GCs through increased levels of proliferative indices (Ki67^+^, control treatment: *n* = 12 vs MDK treatment: *n* = 12, *P* = 0.0354), reduced levels of apoptotic markers (Cleaved C3, control treatment: *n* = 16 vs MDK treatment: *n* = 17, *P* = 0.0482), and increased gonadotropin responsiveness (FSHR^+^, control treatment: *n* = 18 vs MDK treatment: *n* = 18, *P* = 0.0464). This evidence suggests that MDK may become one of the critical paracrine effectors that coordinate follicular niche maturation. A spatial analysis of ENPEP^+^ stromal layers revealed MDK-dependent proliferative amplification by Day 4 (Supplementary Fig. S3c, d; control treatment: *n* = 46 vs MDK treatment: *n* = 71, *P* = 0.0418), demonstrating autonomous expansion mechanisms independent of follicular volume scaling. We also examined the levels of hormone response (*Fshr* and *Lhcgr*) and steroid hormone synthesis (*Cyp11a1*, *Cyp19a1*, and *Hsd17b1*) in GCs cultured in vitro. The results showed that the responsiveness of GCs to FSH and LH was increased by MDK supplementation, but the efficiency of steroid hormone synthesis was not significantly increased (Supplementary Fig. S3e; control treatment: *n* = 9 vs MDK treatment: *n* = 9), which may also be related to the heterogeneity of follicle culture in vitro. However, these peri-follicular stromal subtypes maintain structural homeostasis through mitotic synchronization, suggesting an intrinsic self-organizing program that dynamically calibrates the size of the stromal compartment to meet the stage-specific developmental demands. The MDK-mediated expansion of ENPEP^+^ cells indicates either autocrine regulatory circuits or follicle-derived signal potentiation underlying stromal niche plasticity.

Pharmacological MDK supplementation accelerated folliculogenesis in vitro by increasing GC expansion. Conversely, the administration of the MDK inhibitor iMDK (10 μM) not only blocked MDK signaling but also abrogated downstream PI3K activity. This inhibition significantly reduced the follicle diameter compared with that of the control cells by Day 5 (0 μM iMDK: *n* = 26 vs 10 μM iMDK: *n* = 26, *P* < 0.01), with more pronounced effects on Day 8 (Fig. 6e, f; *P* < 0.0001). IF staining confirmed that iMDK treatment attenuated GC proliferation, directly linking MDK-dependent signaling to follicular growth kinetics (Fig. 6g, h; control treatment: *n* = 19 vs MDK treatment: *n* = 16, *P* = 0.0172). These findings establish MDK as a critical regulator of follicular development.

Functional analyses of antral-stage oocytes from the culture system revealed conserved maturation competence but impaired developmental potential. Mechanically isolated cumulus–oocyte complexes (COCs) exhibited comparable meiotic maturation rates (>60% MII attainment) across conditions. However, in vitro fertilization (IVF) outcomes showed markedly reduced developmental competence compared to in vivo-matured (IVM) controls, with no significant differences in cleavage-stage progression (2-cell) or blastocyst formation frequency (Fig. 6i; control treatment: *n* = 3 vs MDK treatment: *n* = 3). Notably, the MDK-supplemented groups produced blastocysts with an improved morphological integrity (Fig. 6j), despite the inherent limitations of the alginate there-dimensional (3D) system in supporting embryogenic potential — a persistent challenge in artificial folliculogenesis. While MDK supplementation did not directly increase the gamete quality, its ability to modulate the follicular growth rate and optimize the microenvironment likely mitigated oocyte stress during in vitro development. Future studies will establish embryonic transfer protocols to assess the full-term developmental capacity, with expanded datasets clarifying the role of MDK in preserving oocyte developmental competence through indirect modulation of the follicular niche.

### The role of the perifollicular stromal niche in the stage-specific microenvironmental coordination of folliculogenesis

**Fig. 7:**
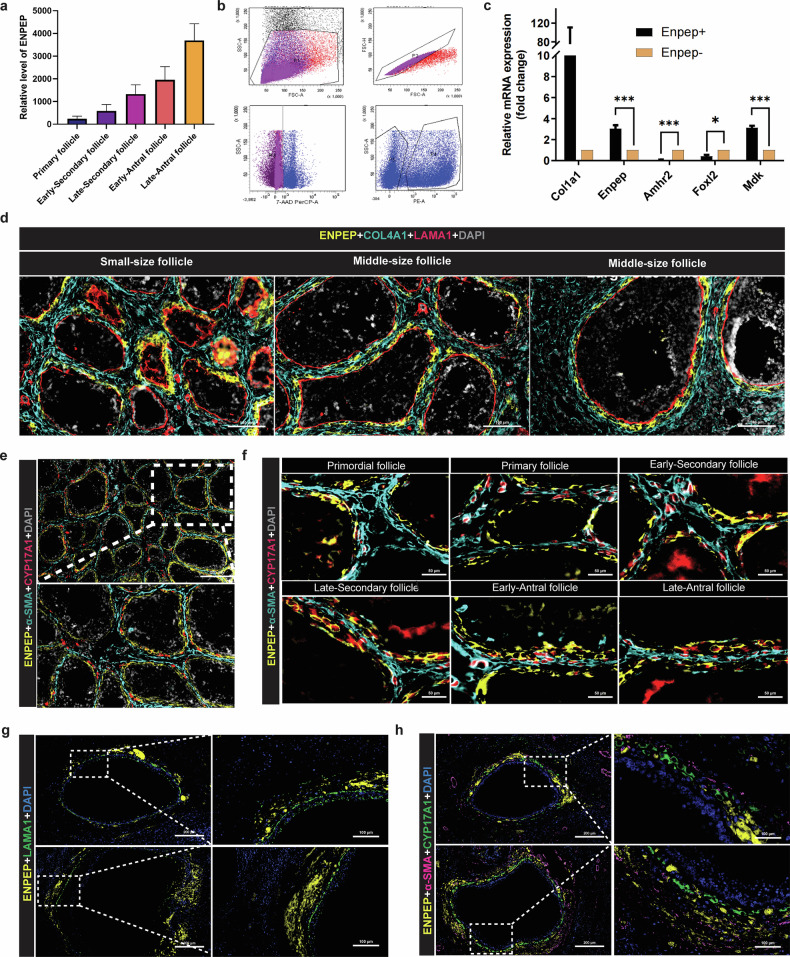
Role of perifollicular SCs in stage-specific microenvironmental coordination. **a** The relative expression level of Enpep in follicles at different developmental stages was determined by performing IF staining. Twelve ovaries were collected for sectioning. **b** Perifollicular SC sorting strategy using the ENPEP-PE antibody. **c** qPCR analysis of *Col1a1*, *Enpep*, *Amhr2*, *Foxl2* and *Mdk* expression in ENPEP^+^ and ENPEP^–^ cells after sorting (^*^P < 0.05 and ^***^P < 0.001, Student’s *t*-test). **d** Spatial location of perifollicular SCs (ENPEP, yellow), collagen IV (COL4A1, bright blue) and laminin (LAMA1, red) in different sizes of follicles in mouse ovaries determined using multiplex IF staining. **e** General spatial location of perifollicular SCs (ENPEP, yellow), ovarian microvessels (α-SMA, bright blue) and theca cells (CYP17A1, red) in mouse ovaries determined using multiplex IF staining. **f** Spatial location of perifollicular SCs (ENPEP, yellow), ovarian microvessels (α-SMA, bright blue) and theca cells (CYP17A1, red) in different sizes of follicles in the mouse ovary, as determined using multiplex IF staining. **g** Spatial locations of perifollicular SCs (ENPEP, yellow) and laminin (LAMA1, green) in the human ovary, as detected using IF staining. **h** Spatial locations of perifollicular SCs (ENPEP, yellow), ovarian microvessels (α-SMA, bright blue) and theca cells (CYP17A1, red) in the human ovary, as detected using multiplex IF staining.

Spatiotemporal profiling revealed ENPEP as a perifollicular SC-specific marker, with paracrine MDK secretion persisting throughout follicular development. The quantitative analysis of IF staining revealed stage-dependent ENPEP upregulation in primary-to-antral follicles (Fig. 7a; Supplementary Fig. S4a; primary follicles, *n* = 33 vs early-secondary follicles, *n* = 77 vs late-secondary follicles, *n* = 62 vs early-antral follicles, *n* = 43 vs late-antral follicles, *n* = 25), consistent with the dynamics of 3D follicular cultures in vitro. ENPEP^+^ perifollicular SCs and ENPEP- SCs were isolated via fluorescence-activated cell sorting (FACS) (Fig. 7b), and the results showed that ENPEP^+^ perifollicular SCs exhibited a fibroblastic morphology (Supplementary Fig. S4b) and distinct molecular signatures: ENPEP^+^ SCs had higher expression of *Col1a1* and *Mdk* with no expression of *Foxl2*; ENPEP^−^ SCs had higher expression of *Foxl2* and *Amhr2* with no expression of *Mdk* (Fig. 7c; Supplementary Fig. S4c). This molecular stratification confirms that ENPEP^+^ perifollicular SCs constitute a functionally specialized stromal subtype that coordinates ECM remodeling and paracrine signaling during folliculogenesis.

Multiplex IF staining with antibodies against ENPEP (perifollicular SCs), collagen IV (COL4A1, GC–theca cell boundary) and laminin (LAMA1, basement membrane) resolved the location of ENPEP^+^ perifollicular SCs at the basement membrane, overlapping collagen IV zones and scattering within the theca cell–SC interface (Fig. 7d). We employed α-SMA (theca externa microvasculature) and CYP17A1 (theca interna steroidogenic cells) as markers to map the localization of perifollicular SCs relative to the follicular theca layers. Analyses of different developmental stages revealed that ENPEP^+^ perifollicular SCs emerged in early secondary follicles alongside α-SMA^+^ microvessels, with CYP17A1 expression remaining low. Late secondary/antral follicles exhibited ENPEP–CYP17A1 colocalization in concentric perifollicular layers (Fig. 7e, f). Human ovarian sections recapitulated this architecture: ENPEP^+^ perifollicular SCs localized to laminin-defined basement membrane peripheries and were spatially interdigitated with CYP17A1^+^ theca cells (Fig. 7g, h). Strikingly, ENPEP and CYP17A1 displayed inverse expression gradients during secondary follicle maturation–ENPEP dominance in the early stages that transitioned to CYP17A1 enrichment in the late phases, revealing lineage coordination between perifollicular SCs and theca cells and indicating that perifollicular SCs most likely differentiate into theca cell progenitors. This spatiotemporal complementarity implies functional synergy during follicular niche maturation.

### Synchronization of dysfunction of perifollicular SCs and folliculogenesis disorders in aging ovaries

**Fig. 8:**
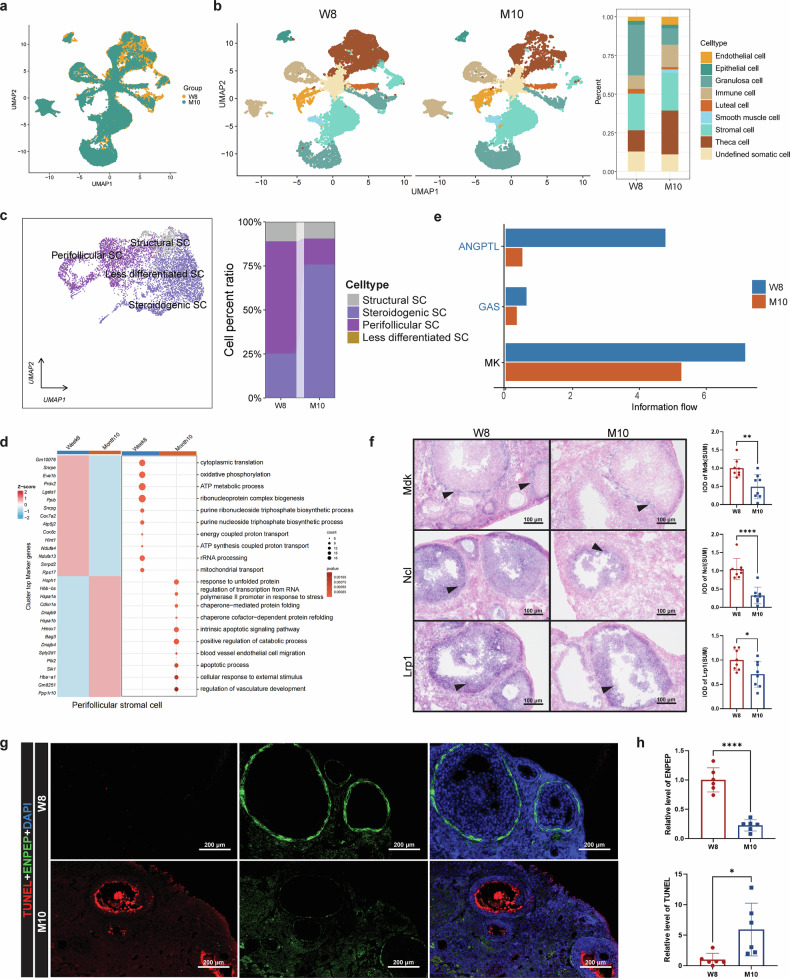
Dysfunction of perifollicular SCs in aging ovaries. **a** UMAP plot of ovarian SCs colored based on samples collected at W8 and M10. **b** UMAP plot of 9 ovarian cell populations identified at W8 and M10. **c** UMAP plot of 4 specific SC subtypes identified at W8 and M10. **d** Comparison of signaling pathways that were enriched with specific cell markers of perifollicular SCs among follicular SCs at W8 and M10. **e** Comparison of ANGPTL, GAS, and MK signaling flow in mouse ovaries at W8 and M10. **f** Representative images showing the locations and mRNA expression levels of *Mdk*, *Ncl* and *Lrp1* detected using ISH in mouse ovary sections at W8 and M10. Six ovaries were collected from the mice in each group (^*^P < 0.05, ^**^P < 0.01, and ^****^P < 0.0001, Student’s *t*-test). Arrowheads indicate posi*t*ive signals in the ovaries. **g** Spatial locations of apoptotic (TUNEL, red) and perifollicular SCs (ENPEP, green) in the mouse ovary detected using IF staining. **h** Relative levels of TUNEL and ENPEP staining in mouse ovaries at W8 and M10. Six ovaries were collected from the mice in each group (^*^P < 0.05 and ^****^P < 0.0001, Student’s *t*-test).

Ovarian aging progresses asynchronously with systemic aging and is influenced by psychosocial and environmental factors. We performed scRNA-seq on the ovaries of 10-month-old mice and integrated datasets with the ovaries of 8-week-old mice to assess stromal subtype dynamics during aging (Fig. 8a). We identified nine ovarian cell populations in the ovaries (Fig. 8b), revealing age-dependent GC depletion but stable stromal proportions. Subclustering of SCs revealed conserved structural SCs across ages, with perifollicular SCs dominating young ovaries (folliculogenesis-associated) versus steroidogenic SCs comprising 75% of the aged stroma (Fig. 8c). The enrichment analysis of differentially expressed genes demonstrated functional reprogramming: young SCs engaged in oxidative phosphorylation and mitochondrial metabolism compared with the enrichment of angiogenesis and apoptosis in their aged counterparts (Fig. 8d; Supplementary Fig. S5a–c). Cell communication analyses revealed an age-related attenuation of pro-folliculogenic pathways (ANGPTL, GAS, and MK). These shifts correlated with a decrease in stromal function — which was characterized by the accumulation of oxidative stress, a reduction in hormone secretion, and the activation of apoptosis — and the establishment of stromal niche erosion as a hallmark of ovarian aging.

The CellChat analysis revealed an age-dependent attenuation of pro-folliculogenic pathways (ANGPTL, GAS, and MK) in the ovaries of 10-month-old mice (Fig. 8e). Spatial validation via ISH confirmed that stromal *Mdk* expression was downregulated and that the expression of its receptors (*Ncl* and *Lrp1*) was reduced in aged GCs (Fig. 8f; W8, *n* = 6 vs M10, *n* = 6). TUNEL assays revealed increased apoptosis in aging ovaries, with fragmented perifollicular stromal niches surrounding atretic follicles (Fig. 8g, h; W8, *n* = 6 vs M10, *n* = 6), indicating that stromal dysfunction and MK signaling collapse occur during age-related folliculogenic failure. The scRNA-seq of human ovaries (young subjects: *n* = 3; aged subjects: *n* = 3) corroborated these findings (Supplementary Fig. S5d). Aged SCs exhibited decreased ENPEP expression, although TCF21^+^/TMEM100^+^ perifollicular stromal subtypes persisted (Supplementary Fig. S5e). The CellChat analysis revealed the conserved downregulation of the MDK–NCL axis in aged human ovaries (Supplementary Fig. S5f), suggesting the evolutionary conservation of MK signaling erosion during follicular decline. The therapeutic potential of reactivating the MK pathway to rescue stromal niche function warrants a mechanistic exploration.

## Discussion

Mammalian ovarian folliculogenesis relies on spatiotemporally coordinated interactions among cells in the follicular niche^32–35^. In recent years, the ovarian stroma has attracted increasing attention because of its dual role in providing structural support for follicular development and undergoing dynamic changes associated with both physiological and pathological states^36^. Single-cell transcriptomic studies have further revealed functionally distinct stromal subpopulations, highlighting the heterogeneity and complexity of this compartment^37,38^. Importantly, emerging evidence suggests that stromal remodeling plays a fundamental role in ovarian aging, underscoring the potential of targeting the stromal niche to restore the function of the follicular microenvironment — a notion that represents a significant advance in our understanding of ovarian physiology^39^. Through integrated multi-omics analyses, we decoded the stromal ecosystem into four functional subtypes: structural SCs (matrix organization), perifollicular SCs (paracrine signaling), less differentiated SCs (multipotent differentiation), and steroidogenic SCs (hormone synthesis). Perifollicular SCs function as signaling hubs, directing follicular maturation through stage-specific secretory modules (e.g., the MDK–NCL axis), thereby redefining the architecture of the ovarian niche. Our work establishes SCs as master regulators of follicular niche coordination, with perifollicular SCs serving as dynamic architects bridging structural support and developmental signaling across reproductive windows.

ENPEP^+^ perifollicular SCs formed concentric layers around developing follicles directly adjacent to theca cell compartments (Fig. 7d, e). In vitro-cultured perifollicular SCs exhibited a fibroblastic morphology and spontaneously differentiated into steroidogenic cells expressing theca cell markers (STAR and CYP17A1) without hormonal induction, which may include theca cells, suggesting the intrinsic differentiation capacity of perifollicular SCs (Fig. 7f). While theca cells are known to be derived from ovarian stromal precursors^1,13,40^, their precise ontogeny remains undefined. Our findings challenge the classical fibroblast-origin hypothesis by suggesting that perifollicular SCs — a defined stromal subtype — serve as theca cell progenitors. Stromal remodeling is a continuous process underlying both follicular development and ovarian aging, yet its molecular mechanisms remain poorly defined. Previous studies have implicated progenitor cells and lineage differentiation in this process. Our findings show that in aged ovaries, the activity of the MK signaling pathway is downregulated in perifollicular SCs, accompanied by a reduction in the number of perifollicular SCs. Importantly, emerging evidence indicates that supplementation with SCs or their components can partially restore ovarian function^38^, highlighting the therapeutic potential of targeting the stromal niche for treating ovarian aging. Notably, perifollicular SC-derived theca cell precursors lacked LH receptor (LHCGR) expression and steroidogenic enzyme activity (Fig. 7g), indicating the initiation of gonadotropin-independent differentiation. Primate studies have shown that ovarian SC transplantation restores theca cell function without requiring LH responsiveness^41–44^. Collectively, through spatial, molecular, and functional validation experiments, we found that perifollicular SCs may be a type of theca progenitor cell, reconciling longstanding debates about the origins of theca cells.

Ovarian SCs are presumed to originate from mesenchymal progenitors^45,46^, yet their developmental hierarchy and functional specialization remain poorly defined. We identified *Aldh1a2* as a marker of less differentiated ovarian SCs localized to the cortical stroma and surface epithelium (Fig. 2d). Aldehyde dehydrogenase 1 (ALDH1) is a marker of normal tissue stem cells and is involved in self-renewal, differentiation, and self-protection^47,48^. Studies have demonstrated Aldh1a1- and Aldh1a2-expressing stem cell niches in ovarian surface epithelial cells and subsurface regions^23,49^. Pseudotemporal ordering positioned Aldh1a2^+^ cells at the root of stromal differentiation trajectories (Fig. 2f–i), with their age-dependent decline correlating with ovarian functional attrition — a phenomenon extending beyond follicular depletion to encompass stromal niche degeneration.

P7 emerges as a critical developmental node that is marked by less differentiated SC branching in pseudotime trajectories and the specific inactivation of *Bclaf1* regulatory activity in the SCENIC analysis^50,51^, suggesting that this time point potentially initiates our proposed three-cell model. As a direct HIF-1 target that mediates HIF-1α transcriptional regulation and stabilization in response to hypoxia, *Bclaf1* has pro-angiogenic functions^52^. HIF-1 is constitutively expressed during reproductive system development and orchestrates angiogenic processes and progenitor cell functionality. Notably, transient HIF-1α suppression in the early reproductive phase appears to protect against hypoxic oocyte damage, although reactivation occurs at later stages^53–55^. This regulatory dynamic coincides with ovarian activation on Day 7, which is marked by follicular stimulation waves, GC proliferation, and the initiation of theca cell differentiation. This temporal coordination raises a key question: could SC differentiation and *Bclaf1* suppression at this juncture establish preparatory conditions for subsequent follicular expansion?

Our folliculogenesis studies employed a 3D in vitro culture system engineered to recapitulate key aspects of the ovarian mechanical niche. Building upon 2D platforms, this 3D architecture establishes a physiomimetic microenvironment through biomaterial-guided spatial organization — alginate hydrogels replicate key physical parameters, including tissue-specific mechanical compliance (100–800 Pa Young’s modulus) and spatial confinement^56–58^. Biomechanical profiling revealed ovarian heterogeneity, with regional stiffness variations within ovarian compartments (cortical stroma < medullary stroma) reflecting distinct ECM compositions. Follicular mechanoadaptation exhibits size-dependent dynamics, with larger follicles displaying an enhanced mechanoadaptation to microenvironments with increased stiffness^59^. These observations support our hypothesis that SCs may orchestrate micromechanical force gradients within our triple-cell model to drive follicular maturation. Furthermore, this tunable culture platform enables the systematic investigation of mechanotransduction pathways relevant to the development of stem cell-based therapies for ovarian dysfunction disorders, particularly premature ovarian insufficiency.

SC pathophysiology extends beyond natural aging processes to critically influence ovarian disorders. In polycystic ovary syndrome (PCOS), which is characterized by oligo-anovulation, hyperandrogenemia, and polycystic ovarian morphology, stromal abnormalities manifest as cortical stiffening, compartment-specific hyperplasia, and antral follicle arrest^60,61^. Ovaries from patients with PCOS exhibit stromal hypervascularization and organomegaly, with superovulation regimens partially reversing stromal pathology in responsive patients through the rescue of follicular maturation. Mechanistic investigations have implicated angiopoietin-like 4 (ANGPTL4)^62^ and TWEAK^29^ signaling as stromal–epithelial mediators in PCOS pathogenesis, although the precise regulatory networks involved remain undefined. Our spatial analyses revealed coordinated perifollicular stromal expansion with folliculogenesis, demonstrating differentiation potential toward theca–interstitial cell lineages^63,64^. Pathological stromal hyperproliferation coupled with excessive theca-committed differentiation establishes a self-reinforcing cycle of stromal expansion and androgen excess. This cellular taxonomy redefines stromal contributions to PCOS pathobiology, revealing therapeutic targets within the stromal signaling axis.

## Materials and methods

### Animals and preparation of cell suspensions

C57BL/6J mice were purchased for scRNA-seq and ovarian section staining from the Animal Experiment Center of Nanjing Medical University (Nanjing, China). We used the single-cell data of P5 from the published reports^65^ from a public database. Bilateral ovaries from at least three mice at each time point, as an ovarian single-cell pool, were dissected from female mice at P3, P7, W3, W8 and month (M) 10. These selected time points for sequencing represent the key stage of a series of cellular events involving ovarian and follicular development. Moreover, ovaries from P7, W3, and W8 mice were collected and embedded in OCT compound. Ten-micron sections were cut for the experiment using the 10× Visium platform. Isolated ovaries were cut into small pieces and incubated with 0.25% trypsin (Gibco, Grand Island, NY, USA) and collagenase II (0.2%, Sigma–Aldrich, C2-22-1G, Darmstadt, Germany) for 6 to 8 min at 37 °C to obtain single-cell populations. The tissues were disaggregated with a pipette to generate single cells, and the solution was filtered through 40-μm cell strainers (BD Falcon, 352340, USA) and washed 2 times with PBS containing 0.04% BSA. The cell viability was acceptable after staining with 0.4% Trypan blue; it was >80%, and the cell concentration (1000 cells/μL) was checked to meet the requirements for sequencing, as was the single-cell rate (no connected cells were observed during cell counting). All animal experiments were conducted under the guidance of the Laboratory Animal Management Committee (Jiangsu Province, China) and approved by the Institutional Animal Care and Use Committee of Nanjing Drum Tower Hospital (SYXK 2021–0509).

### Ethics statement

The mice were maintained under specific pathogen-free conditions in a controlled environment at a temperature of 20 ± 2 °C and a humidity of 50%–70% on a 12:12-h light/dark cycle, with food and water provided ad libitum. Animal care and experimental procedures were performed in accordance with the guidelines of the Experimental Animal Management Committee (Jiangsu, China) and were approved by the Ethics Review Board for Animal Experiments of the Affiliated Drum Tower Hospital of Nanjing University Medical School. All applicable institutional and/or national guidelines for the care and use of animals were followed.

### Preparation of single-cell libraries and sequencing

Single-cell suspensions of ovary cells were captured on a 10× Chromium system (10× Genomics). Single-cell mRNA libraries were subsequently generated using single-cell 3′ reagent V3 kits according to the manufacturer’s protocol. After the generation of gel bead-in-emulsions, reverse transcription reactions were barcoded using unique molecular identifiers (UMIs), and cDNA libraries were then amplified by PCR with appropriate cycles. Subsequently, the amplified cDNA libraries were fragmented and then sequenced on the Illumina NovaSeq 6000 platform (Illumina, San Diego, CA, USA). Furthermore, the “mkfastq” module of Cell Ranger was used to produce FASTQ files with raw base call files as input generated by Illumina sequence alignment.

### 10× Visium processing and sequencing

The obtained ovarian tissues were cut at a thickness of 10 µm and mounted onto the corresponding capture areas on the Visium Spatial Tissue Optimization Slide. Next, the tissues were dehydrated with isopropanol for 1 min followed by staining with hematoxylin and eosin. Sections were mounted in 80% glycerol, and brightfield images were captured using a 3D HISTECH Pannoramic MIDI FL whole-slide scanner at 40× resolution. After permeabilization, the cellular mRNA in the spots was captured by the primers to assess gene expression. All the cDNAs generated from mRNAs captured by primers on a specific spot share a common spatial barcode. Libraries were generated from the cDNAs and sequenced, and spatial barcodes were used to associate the reads back to the images of the tissue sections for spatial mapping of gene expression. Visium spatial gene expression libraries comprise standard Illumina paired-end constructs that are flanked with P5/P7, which is necessary for binding to the Illumina flow cell.

### scRNA-seq data processing and analysis

The Cell Ranger “count” pipeline (version 3.1.0) was applied to the FASTQ data to map the mouse reference genome (version mm10) and process feature–barcode matrices with the default parameters. The number of captured cells in each sample was set to 6000 with the parameter “force-cells” to prevent potential bias during subsequent bioinformatics analysis. The data matrices were then loaded in R (version 4.4.3) using the Seurat package (version 5.2.1). The Seurat object was created based on 2 filtering parameters, “min.cells = 5” and “low.thresholds = 200”, and the exorbitant number of unique genes detected in each cell (i.e., “nFeature_RNA” >8000) was adjusted in each sample to eliminate the empty drops and dying cells and potential doublets/multiplets from subsequent analyses. Moreover, a high percentage of reads mapping to mitochondrial genes were filtered out. After these stringent filters were applied, a high-quality dataset of 58,319 cells was retained for the subsequent downstream analysis. Multiple samples were subsequently processed with “Harmony”. Following the normalization and scaling steps, using the UMAP (a method for visualizing cell clusters in high-dimensional transcriptomic data) technique, a series of commands were executed to visualize cell clusters, including the “RunUMAP” function with a proper combination of the “resolution” and “dims.use,” “FindNeighbors,” and “FindClusters” functions to conduct cell clustering. The “FindAllMarkers” function was used to identify conserved canonical marker genes expressed in clusters with the default parameters. scRNA-seq data from cynomolgus monkeys were downloaded from GSE130664. The scRNA-seq data were analyzed with Seurat V5.2.1 using the same method as that used for the mouse samples.

### 10× Visium data preprocessing

Raw FASTQ files and histology images were processed by sample with SpaceRanger software version 1.2.0, which uses STAR for genome alignment against the mm10 reference genome. We processed the unique molecular identifier (UMI) count matrix using the R package Seurat and normalized the data with SCTransform34 to account for the variance in sequencing depth across data points. The top genes across single cells were identified using the method described by Macosko et al. The cells were clustered based on a graph-based clustering approach and visualized in 2 dimensions using UMAP. We used SPOTlight to integrate the 10× Visium data with the scRNA-seq data to infer the locations of cell subtypes and states within a complex tissue. Furthermore, MIA was performed according to the method described by Moncada et al.

### Pseudotime analysis of single-cell transcriptomes

Cell lineage trajectories were constructed according to the procedure recommended in the documentation for Monocle2, a novel unsupervised algorithm, which reordered the cells to maximize transcriptional similarity based on their differentiation progress, and at the same time, distinguished genes that were activated early and later in differentiation, which contributed to the dissection of the cell differentiation fate, also termed a “pseudotime analysis.” With the gene count matrix serving as the input, a new dataset for Monocle objects was created, and the functions “reduceDimension” and “orderCells” were used to generate the cell trajectory based on pseudotime. In particular, the ordering of genes whose expression differed between clusters in each cell subtype was calculated using the “differentialGeneTest” function in Monocle. In addition, the “BEAM” function was used to calculate the differentially expressed genes at a branch point in the trajectory, and genes with “*q*val < 1 × 10^−4^” are shown in a heatmap. Moreover, the root state (namely, a pre-branch in the heatmap) was set and adjusted after considering the biological meanings of different cell branches.

### GO and KEGG enrichment analyses of the gene set

For a large gene set with more than 100 genes, GO and KEGG enrichment analyses were performed with ClusterProfiler, an R package in Bioconductor, to detect biological processes and signaling pathways related to the differentially expressed genes with a threshold value of “*P*valueCut off = 0.05”, and the top terms are displayed.

### Analysis of the regulon activity of transcription factors with SCENIC

The SCENIC algorithm was developed to assess the regulatory network of TFs and to discover regulons (namely, TFs and their target genes) in individual cells^40^. Using the standard pipeline, the gene expression matrix with gene names in rows and cells as columns was input into SCENIC (version 0.9.1). The genes were filtered with the default parameters, and 9208 genes were ultimately available in the RcisTarget database, the mouse-specific database (mm10) that is used as the default in SCENIC. The coexpressed genes for each TF were constructed with GENIE3 software, followed by a calculation of Spearman’s correlation coefficients between the TFs and the potential targets, and then the “runSCENIC” procedure assisted in generating the GRNs (also termed regulons). Finally, regulon activity was analyzed using AUCell (Area Under the Curve) software.

### Patient sample collection and data analysis

The paraffin sections of ovarian tissues from patients of childbearing age who were involved in this study were obtained from Nanjing Drum Tower Hospital, Affiliated Hospital of Medical School, Nanjing University, from 2021 to 2022. A total of three patients underwent extensive abdominal hysterectomy combined with bilateral salpingo-oophorectomy for malignant cervical tumors. The frozen ovarian sections of the patients of childbearing age who were involved in this study were obtained from Women’s Hospital of Nanjing Medical University between 2024 and 2025. One patient underwent right ovariectomy because of an adult-grade GC tumor with cystic changes and calcification in the left ovary. All of the patients’ sections revealed no pathological changes in the ovaries. This study was approved by the Ethics Committee of Research and Ethics Committee of Drum Tower Hospital (approval no. SC2018-001-04 on October 27, 2020) and Research and Ethics Committee of Drum Tower Hospital (approval no. 2025KY052-001 on June 25, 2025). All procedures performed in studies involving human participants were conducted in accordance with the ethical standards of the institutional and Chinese research committee and with the 1964 Declaration of Helsinki and its later amendments or comparable ethical standards. Informed consent was obtained from all the individual participants who were included in this study. The scRNA-seq data from young women (*n* = 3) and old women (*n* = 3) were downloaded from GSE255690. The scRNA-seq data were analyzed with Seurat V5.2.1 using the same method used for the animal samples.

### ISH with a digoxigenin (DIG)-labeled RNA probe

The ovarian sections were incubated at 37 °C and fixed with 4% PFA at room temperature for 20 min. After acetylation, the preheated probes (Supplementary Table S4) were evenly added to the sections and hybridized at 60 °C overnight. The sections were washed with post-hybridization washing solution, MABT solution and RNA washing solution and blocked at room temperature for 1 h. The AP solution of the anti-DIG antibody was diluted 1:2500, and the antibody was incubated with the sections at 4 °C overnight. We cleaned the sections with the MABT solution at room temperature 4 times and with N^TM^ once, added the chromogenic solution (Beyotime Biotechnology, C3206) to the sections, rinsed the sections, and then stained the nuclei. After being dyed, the sections were treated with double-distilled water, a series of gradient alcohol and xylene solutions and finally mounted with SlowFade® Gold antifade reagent (Life Technologies).

### Follicle culture

Two-layered secondary follicles were mechanically isolated from 12-day-old female F1 hybrid mice (C57BL/6J×DBA/2J) using insulin-gauge needles in L15 media (Invitrogen, Carlsbad, CA) containing 1% fetal calf serum (FCS). Individual follicles were maintained in minimal essential medium (αMEM)/1% FCS at 37 °C and 5% CO_2_ for 2 h before encapsulation. Only those follicles that displayed the following characteristics during the 2-h preincubation period were selected for encapsulation and culture: (1) diameter of 130–150 µm, (2) intact nature with some attached fibroblast-like theca cells, and (3) a visible immature oocyte that was round and centrally located within the follicle. The selected follicles were then encapsulated into 0.25% alginate beads. The alginate beads were incubated in the encapsulation solution for 2 min to cross-link the alginate and then rinsed with culture media (αMEM supplemented with 10 mIU/mL recombinant FSH, 3 mg/mL BSA, 1 mg/mL bovine fetuin, 5 μg/mL insulin, 5 μg/mL transferrin, and 5 ng/mL selenium). Alginate beads containing a single follicle were plated at a density of one follicle per well in 96-well plates in 100 μL of culture media. Encapsulated follicles were cultured at 37 °C with 5% CO_2_ for 12–14 days^66^.

### IVM

The COCs were mechanically isolated from in vitro-cultured follicles and placed in MEMα (Gibco, 32561037) (containing 10% fetal bovine serum (Gibco, 10270106), 10 ng/mL EGF (Gibco, 53003-018), and 1.5 IU/mL human chorionic gonadotropin (Sansheng Pharmaceutical)) for maturation. The PBE rate was analyzed after culture in IVM medium for 14 h.

### IVF

The epididymis tails of fertile male mice were collected and placed in 100 µL of human tubal fluid (HTF) medium (MR-070, Sigma, St. Louis, MO, USA), after which the sperm were released by cutting the epididymis tails for capacitation to achieve IVF. Oocytes were obtained after IVM and placed in 50 µL of HTF medium until insemination. After sperm capacitation, 10 µL of HTF medium containing spermatozoa was added to droplets containing COCs. The oocytes were recovered from the droplets of HTF medium 2 h after insemination and were subsequently washed 3 times with KSOM (M1430, Nanjing Aibei Biotechnology Co., Ltd, China) to remove residual GCs and spermatozoa around the oocytes. Inseminated oocytes were maintained in 30 µL of KSOM. All the cellular cultures were maintained at 37 °C in a 5% carbon dioxide atmosphere. The ratio of the formation of 2-cell embryos was determined by dividing the number of 2-cell embryos by the number of MII oocytes. The ratio of the formation of blastocyst embryos was determined by dividing the number of blastocyst embryos by the number of 2-cell embryos.

### IHC

Ovarian samples were removed and fixed with a 10% formalin solution at room temperature overnight; dehydrated in 70%, 80%, 90%, 95% and 100% ethanol solutions; and then embedded in paraffin. Tissues were sectioned at a 5 μm thickness, deparaffinized, and dehydrated with xylene and an ascending series of alcohol solutions. After antigen retrieval, the sections were incubated with the primary antibody (Supplementary Table S4) at 4 °C overnight and then were washed with a TBST solution at room temperature 3 times. Then, the sections were incubated with a biotin-labeled secondary antibody at room temperature for 30 min, washed with TBST solution, and developed using a DAB peroxidase substrate kit (Zsbio, Beijing, China).

### IF staining

Sections of ovaries were deparaffinized and rehydrated with xylene and an alcohol gradient, and heat-induced antigen retrieval was performed in 10 mM sodium citrate buffer (pH 6.0). Sections of follicles were fixed with a special 4% PFA fixative containing calcium ions and embedded after dehydration using a gradient of ethanol solutions^66^. Three follicles were collected together as one sample. After permeabilization with 1% Triton X-100 in PBS, the sections were blocked with 3% BSA in PBS for 60 min at room temperature. The sections were incubated with primary antibodies (Supplementary Table S4) diluted in blocking solution at 4 °C overnight and then were washed with the TBST solution 3 times for 5 min each. We incubated the sections with secondary antibodies for 45 min and then counterstained them with DAPI (Life Technologies, Carlsbad, USA) for 10 min. The sections were mounted using SlowFade® Gold Antifade Reagent (Life Technologies) and examined with a confocal laser scanning microscope (Leica, Wetzlar, Germany).

### Multiplex IF staining

The ovarian tissue was frozen with liquid nitrogen, embedded in OCT compound and sliced at a thickness of 10 μm. The slices were incubated at 37 °C for 3 min, immersed in 4% PFA for 30 min, sterilized and washed three times for 1 min each, after which multiplex IF staining was performed using a research kit (Absin, China, abs50012). Finally, images were captured using fluorescence microscope (Leica, Wetzlar, Germany).

### FACS

The ovaries of 3-week-old mice were washed with 1× PBS 3 times, digested with 0.1% trypsin/EDTA (Sigma, T8003) and 0.8 mg/mL type I collagenase (Worthington, LS004197) at 37 °C, harvested as single-cell suspensions and resuspended in 1× PBS. The cells were incubated with a phycoerythrin (PE)-conjugated Enpep antibody at 4 °C for 60 min and then sorted using a FACScan flow cytometer (Becton, Dickinson and Company, Franklin Lakes, NJ).

### RNA isolation from follicles and quantitative real-time PCR (qRT-PCR)

Five follicles were collected as one sample. Total RNA extracted from the follicles was reverse transcribed into cDNA using a single-cell sequence-specific amplification kit (P621, Nanjing Vazyme Biotech Co., Ltd, China). Then, the cDNA was diluted to 200 ng/µL. Quantitative real-time PCR was conducted on a qTOWER³ real-time PCR thermal cycler (Analytik, Jena, Germany) with a SYBR green PCR kit (Q231, Nanjing Vazyme Biotech Co., Ltd, China). qPCR was used to determine the relative mRNA expression levels of *Fshr*, *Lhcgr*, *Cyp11a1*, *Cyp19a1*, and *Hsd17b1* after normalization to the mRNA levels of β-actin (*Actb*). The PCR conditions were as follows: 95 °C for 30 s (step 1), followed by 40 cycles of 95 °C for 10 s and 60 °C for 30 s (step 2). qPCR data were analyzed using the 2^–ΔΔCT^ method. A complete list of primer sequences for the target genes is provided in Supplementary Table S4.

### Statistical analysis

The data are presented as the mean ± SD and were obtained from 3 independent experiments. Statistical analyses were performed with GraphPad Prism software (version 9.0), and significant differences were determined with Student’s unpaired *t*-test. Significal significance was set at ^*^P < 0.05, ^**^P < 0.01, ^***^P < 0.001 and ^****^P < 0.0001.

## Supplementary information

Supplementary Information

## Data Availability

Please contact Dr. Sun and Dr. Yan to request all data and reagents described in this article. The scRNA seq and 10× Visium data information and raw data can be found in NCBI Bioproject numbers PRJNA1290881.
