# Supplementary material for: Perifollicular stromal cells sustain folliculogenesis via MDK–NCL signaling in mice and humans
Source: Cell Discov. 2026 Jul 28;12:56. doi: 10.1038/s41421-026-00907-3 (PMC13408105; doi:10.1038/s41421-026-00907-3)
Supplement: Supplementary file 1 — Supplementary Information [file 41421_2026_907_MOESM1_ESM.pdf]

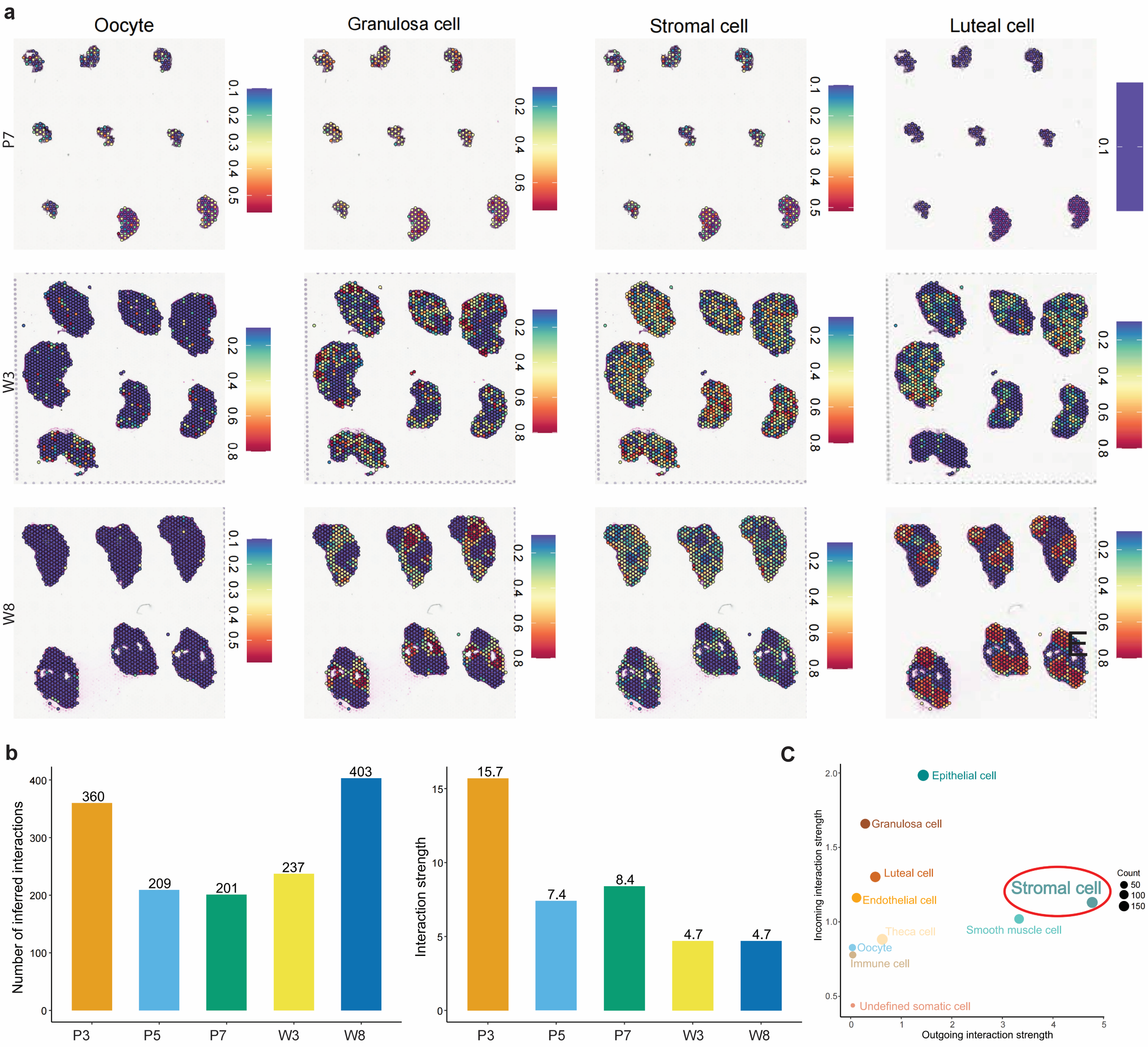
a
Oocyte
Granulosa cell
Stromal cell
Luteal cell
P7
W3
W8
b
Number of inferred interactions
360
209
201
237
403
P3
P5
P7
W3
W8
Interaction strength
15.7
7.4
8.4
4.7
4.7
C
Incoming interaction strength
Outgoing interaction strength
Epithelial cell
Granulosa cell
Luteal cell
Endothelial cell
Stromal cell
Smooth muscle cell
Theca cell
Oocyte
Immune cell
Undefined somatic cell
Count
50
100
150

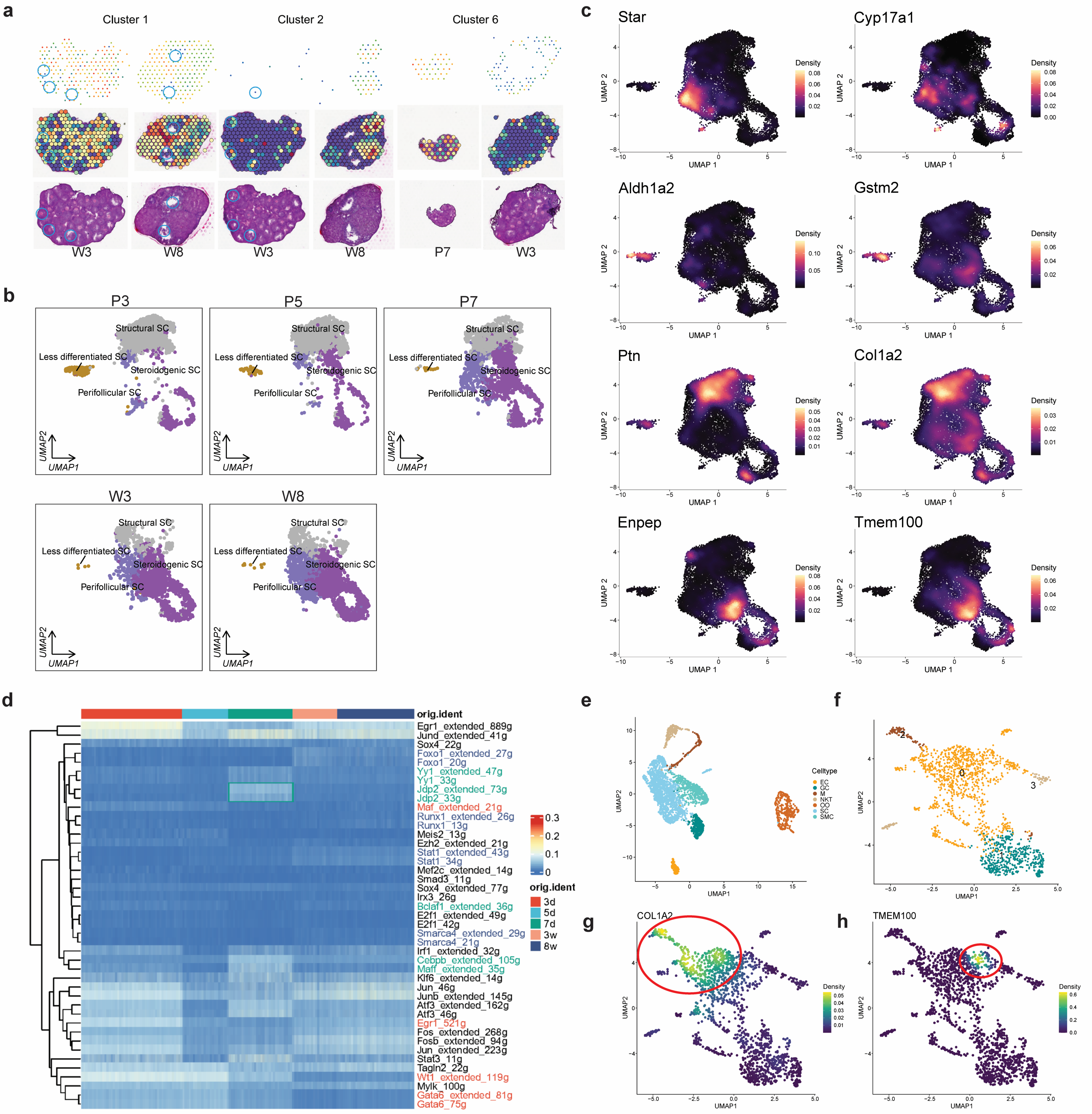
a
Cluster 1
Cluster 2
Cluster 6
W3
W8
W3
W8
P7
W3
b
P3
P5
P7
W3
W8
Structural SC
Less differentiated SC
Steroidogenic SC
Perifollicular SC
UMAP2
UMAP1
c
Star
Cyp17a1
Aldh1a2
Gstm2
Ptn
Col1a2
Enpep
Tmem100
Density
UMAP 1
UMAP 2
d
orig.ident
Egr1_extended_889g
Jund_extended_41g
Sox4_22g
Foxo1_extended_27g
Foxo1_20g
Yy1_extended_47g
Yy1_33g
Jdp2_extended_73g
Jdp2_33g
Maf_extended_21g
Runx1_extended_26g
Runx1_13g
Meis2_13g
Ezh2_extended_21g
Stat1_extended_43g
Stat1_34g
Mef2c_extended_14g
Smad3_11g
Sox4_extended_77g
Irx3_26g
Bclaf1_extended_36g
E2f1_extended_49g
E2f1_42g
Smarca4_extended_29g
Smarca4_21g
Irf1_extended_32g
Cebpb_extended_105g
Maff_extended_35g
Klf6_extended_14g
Jun_46g
Junb_extended_145g
Atf3_extended_162g
Atf3_46g
Egr1_521g
Fos_extended_268g
Fosb_extended_94g
Jun_extended_223g
Stat3_11g
Tagln2_22g
Wt1_extended_119g
Mylk_100g
Gata6_extended_81g
Gata6_75g
0.3
0.2
0.1
0
orig.ident
3d
5d
7d
3w
8w
e
Celltype
EC
GC
M
NKT
OO
SC
SMC
UMAP1
UMAP2
f
0
2
3
UMAP1
UMAP2
g
COL1A2
Density
UMAP1
UMAP2
h
TMEM100
Density
UMAP1
UMAP2

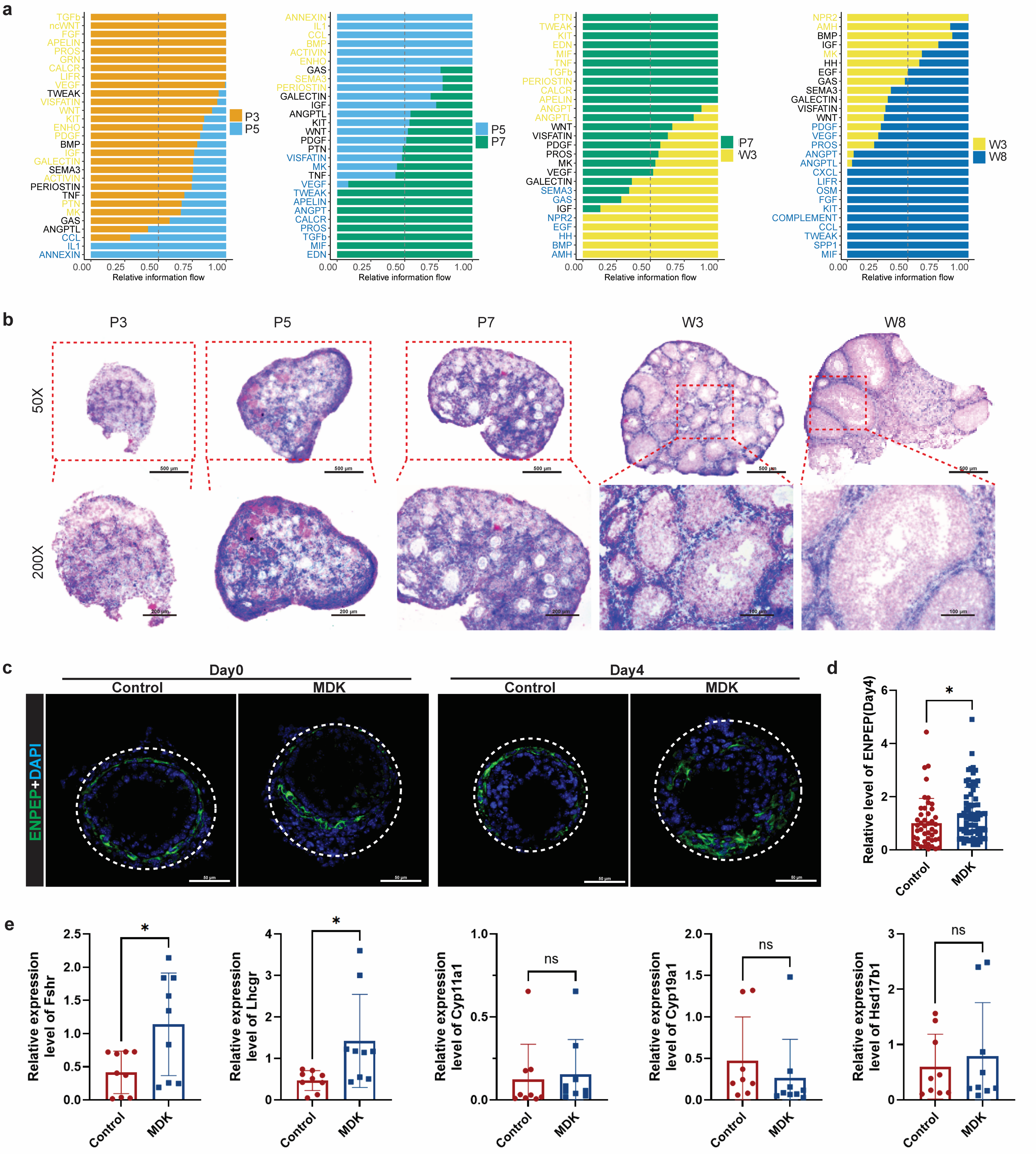
a
Relative information flow
P3
P5
P7
W3
W8
b
P3
P5
P7
W3
W8
50X
200X
c
Day0
Day4
Control
MDK
ENPEP+DAPI
d
Relative level of ENPEP(Day4)
*
Control
MDK
e
Relative expression level of Fshr
Relative expression level of Lhcgr
Relative expression level of Cyp11a1
Relative expression level of Cyp19a1
Relative expression level of Hsd17b1
*
ns
Control
MDK

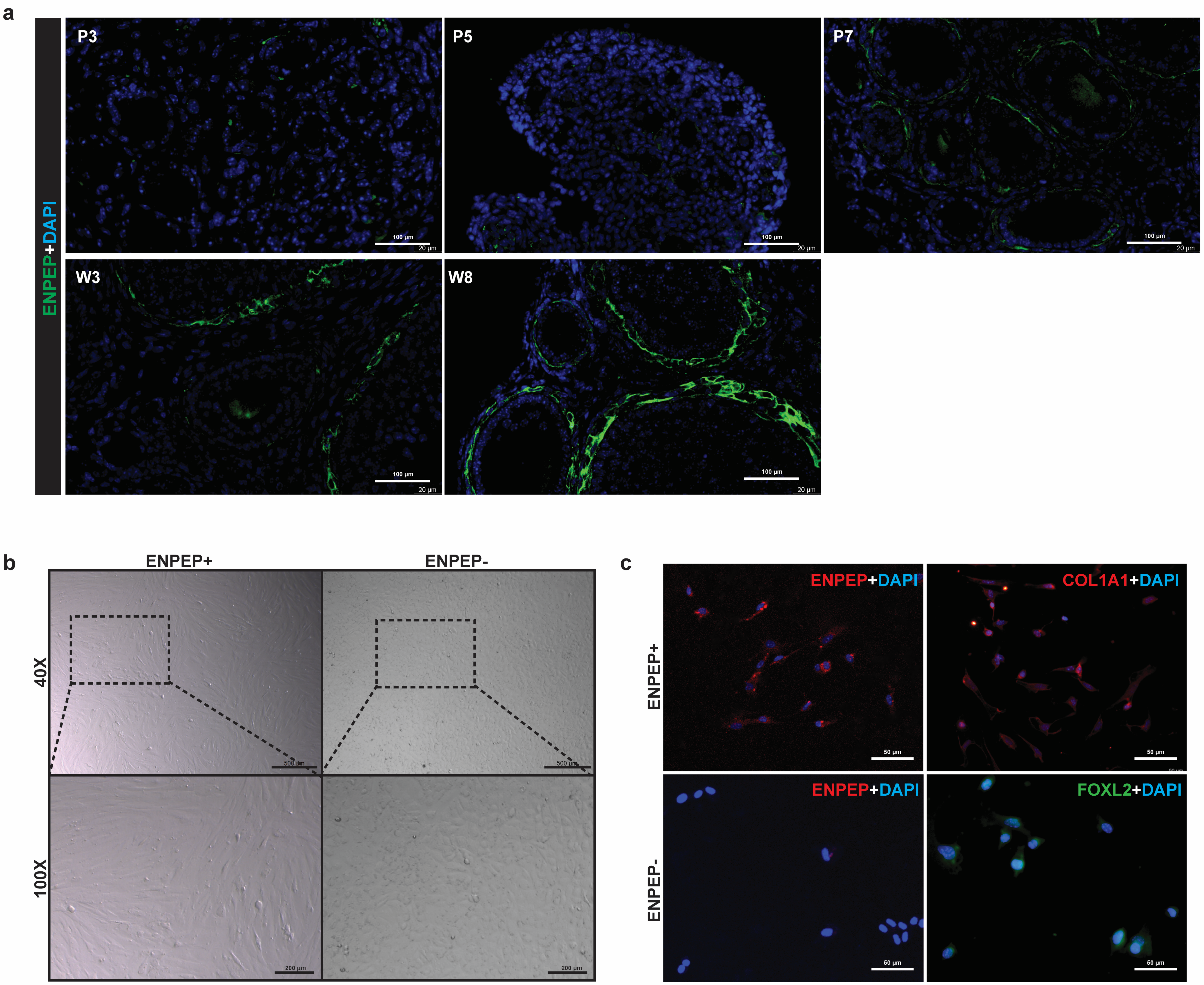
a
ENPEP+DAPI
P3
P5
P7
W3
W8
100 μm
20 μm
b
ENPEP+
ENPEP-
40X
100X
500 μm
200 μm
c
ENPEP+DAPI
COL1A1+DAPI
ENPEP+DAPI
FOXL2+DAPI
ENPEP+
ENPEP-
50 μm

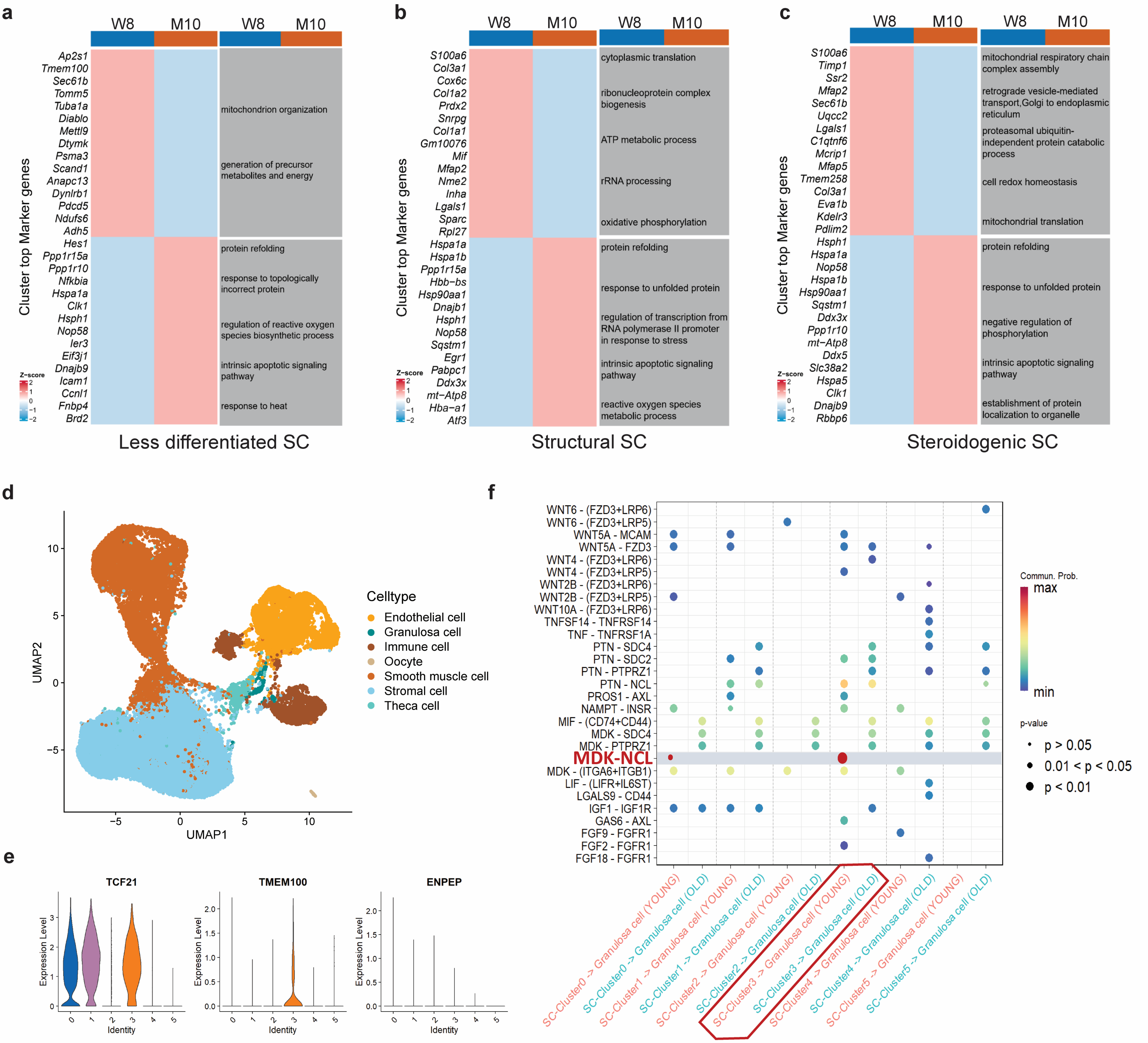

a
W8
M10
W8
M10
Cluster top Marker genes
Ap2s1
Tmem100
Sec61b
Tomm5
Tuba1a
Diablo
Mettl9
Dtymk
Psma3
Scand1
Anapc13
Dynlrb1
Pdcd5
Ndufs6
Adh5
Hes1
Ppp1r15a
Ppp1r10
Nfkbia
Hspa1a
Clk1
Hsph1
Nop58
Ier3
Eif3j1
Dnajb9
Icam1
Ccnl1
Fnbp4
Brd2
mitochondrion organization
generation of precursor metabolites and energy
protein refolding
response to topologically incorrect protein
regulation of reactive oxygen species biosynthetic process
intrinsic apoptotic signaling pathway
response to heat
Z-score
Less differentiated SC
b
W8
M10
W8
M10
Cluster top Marker genes
S100a6
Col3a1
Cox6c
Col1a2
Prdx2
Snrpg
Col1a1
Gm10076
Mif
Mfap2
Nme2
Inha
Lgals1
Sparc
Rpl27
Hspa1a
Hspa1b
Ppp1r15a
Hbb−bs
Hsp90aa1
Dnajb1
Hsph1
Nop58
Sqstm1
Egr1
Pabpc1
Ddx3x
mt−Atp8
Hba−a1
Atf3
cytoplasmic translation
ribonucleoprotein complex biogenesis
ATP metabolic process
rRNA processing
oxidative phosphorylation
protein refolding
response to unfolded protein
regulation of transcription from RNA polymerase II promoter in response to stress
intrinsic apoptotic signaling pathway
reactive oxygen species metabolic process
Z-score
Structural SC
c
W8
M10
W8
M10
Cluster top Marker genes
S100a6
Timp1
Ssr2
Mfap2
Sec61b
Uqcc2
Lgals1
C1qtnf6
Mcrip1
Mfap5
Tmem258
Col3a1
Eva1b
Kdelr3
Pdlim2
Hsph1
Hspa1a
Nop58
Hspa1b
Hsp90aa1
Sqstm1
Ddx3x
Ppp1r10
mt−Atp8
Ddx5
Slc38a2
Hspa5
Clk1
Dnajb9
Rbbp6
mitochondrial respiratory chain complex assembly
retrograde vesicle-mediated transport,Golgi to endoplasmic reticulum
proteasomal ubiquitin-independent protein catabolic process
cell redox homeostasis
mitochondrial translation
protein refolding
response to unfolded protein
negative regulation of phosphorylation
intrinsic apoptotic signaling pathway
establishment of protein localization to organelle
Z-score
Steroidogenic SC
d
UMAP2
UMAP1
Celltype
Endothelial cell
Granulosa cell
Immune cell
Oocyte
Smooth muscle cell
Stromal cell
Theca cell
e
TCF21
TMEM100
ENPEP
Expression Level
Identity
f
WNT6 - (FZD3+LRP6)
WNT6 - (FZD3+LRP5)
WNT5A - MCAM
WNT5A - FZD3
WNT4 - (FZD3+LRP6)
WNT4 - (FZD3+LRP5)
WNT2B - (FZD3+LRP6)
WNT2B - (FZD3+LRP5)
WNT10A - (FZD3+LRP6)
TNFSF14 - TNFRSF14
TNF - TNFRSF1A
PTN - SDC4
PTN - SDC2
PTN - PTPRZ1
PTN - NCL
PROS1 - AXL
NAMPT - INSR
MIF - (CD74+CD44)
MDK - SDC4
MDK - PTPRZ1
MDK-NCL
MDK - (ITGA6+ITGB1)
LIF - (LIFR+IL6ST)
LGALS9 - CD44
IGF1 - IGF1R
GAS6 - AXL
FGF9 - FGFR1
FGF2 - FGFR1
FGF18 - FGFR1
SC-Cluster0 -> Granulosa cell (YOUNG)
SC-Cluster0 -> Granulosa cell (OLD)
SC-Cluster1 -> Granulosa cell (YOUNG)
SC-Cluster1 -> Granulosa cell (OLD)
SC-Cluster2 -> Granulosa cell (YOUNG)
SC-Cluster2 -> Granulosa cell (OLD)
SC-Cluster3 -> Granulosa cell (YOUNG)
SC-Cluster3 -> Granulosa cell (OLD)
SC-Cluster4 -> Granulosa cell (YOUNG)
SC-Cluster4 -> Granulosa cell (OLD)
SC-Cluster5 -> Granulosa cell (YOUNG)
SC-Cluster5 -> Granulosa cell (OLD)
Commun. Prob.
max
min
p-value
p > 0.05
0.01 < p < 0.05
p < 0.01

Supplementary figure Legends

Supplementary Fig. S1 Specific characteristic of different ovarian cell populations. (a) Feature plots of specific marker genes of different ovarian cell types: Oocyte, Granulosa cell, Stromal cell, Luteal cell; (b) The number of inferred interactions and the interaction strength at P3, P5, P7, W3 and W8; (c) The circle plot of the number of interactions among the 10 ovarian cell populations in mice ovary.

Supplementary Fig. S2 Preliminary understanding of stromal subtypes. (a) Cluster1, cluster2 and cluster6 located by RCTD analysis, SpatialFeaturePlot of ovarian spots colored with cluster at 10× magnification; (b) UMAP plot of ovarian SC subtypes splited by P3, P5, P7, W3 and W8; (c) Visualize gene expression density map using plot_density (*Star*, *Cyp17a1*, *Aldh1a2*, *Gstm2*, *Enpep*, *Tmem100*, *Ptn*, *Col1a1*); (d) Heatmap of regulon activity analyzed by SCENIC with default thresholds; (e) UMAP plot of 7 ovarian cell populations in cynomolgus monkey ovary; (f) UMAP plot of ovarian SCs colored by 8 clusters; (g) UMAP plot of *COL1A2* of different cell populations showed by the function of Density in cynomolgus monkey ovary; (h) UMAP plot of *TMEM100* of different cell populations showed by the function of Density in cynomolgus monkey ovary.

Supplementary Fig. S3 The special interactions of SCs in mice ovary. (a) The relative information flow between P3 vs. P5, P5 vs. P7, P7 vs. W3, W3 vs. W8 in mouse ovary. (b) The represented picture of *Mdk* in ovary section at P3, P5, P7, W3 and W8 by ISH; (c) Spatial location of perifollicular SCs (ENPEP, Green) of follicular culturing *in vitro* compared with Day0 and Day4 by immunofluorescence. The dashed circles in white emphasized the localization of follicles; (d) Relative expression level of ENPEP in follicles in Day4, simple follicle was collected for section (*$p < 0.05$, Student’s t test); (e) Relative expression level of *Fshr*, *Lhcgr*, *Cyp11a1*, *Cyp19a1*, *Hsd17b1* analysing by qPCR. Every 5 follicles were collected as one sample, cDNA were obtained by single cell sequence specific amplification kit (P621, Nanjing Vazyme Biotech Co., Ltd., China) (*$p < 0.05$, Student’s t test).

Supplementary Fig. S4 Perifolliclular SCs location and characteristic *in vivo* and *in vitro*. (a) Spatial location of perifollicular SCs (ENPEP, Green) of follicles at at P3, P5, P7, W3 and W8 by immunofluorescence; (b) The represented pictures of ENPEP+ and ENPEP- cells after sorting in P1 generation; (c) Immunofluorescence of ENPEP, COL1A1, and FOXL2 confirmed the characteristic of perifollicular SCs.

Supplementary Fig. S5 Different performance of stromal subtypes in aging ovaries. (a-c) Comparison of signal pathways enriched with specific cell markers in less differentiated SCs, structural SCs, steroidogenic SCs at W8 and M10; (d) UMAP plot of 7 ovarian cell populations in ovaries of young patients (n = 3) and aged patients (n = 3); (e) Violin plot visualization of *TCF21*, *TMEM100* and *ENPEP* in ovaries of young patients and aged patients; (f) Cellchat analysis between 6 SC clusters and GCs by ligands and receptors compared in young patients and aged patients.

Supplementary Table S1

	p_val	avg_log2FC	pct.1	pct.2	p_val_adj	cluster	gene
1	0	2.95114615805782	0.967	0.249	0	0	Col1a1
2	0	3.17987237559947	0.948	0.254	0	0	Dcn
3	0	3.45167845927824	0.861	0.167	0	0	Mfap4
4	0	2.77969084289007	0.786	0.174	0	0	Mmp2
5	0	3.04694441181761	0.665	0.119	0	0	Islr
6	0	2.87067715554087	0.709	0.171	0	0	Ptn
7	0	2.71652285440486	0.675	0.14	0	0	Cdh11
8	0	3.32307301286457	0.677	0.148	0	0	Sfrp1
9	0	2.92987619579761	0.715	0.198	0	0	Lsp1
10	0	3.21719991166005	0.614	0.101	0	0	Tcf21
11	0	3.14585168842458	0.577	0.101	0	0	Ogn
12	0	3.30586554760234	0.586	0.119	0	0	Dlk1
13	0	3.23586011961152	0.563	0.1	0	0	Lum
14	0	2.84105421605064	0.525	0.094	0	0	Lrrc17
15	0	3.02633754477043	0.483	0.08	0	0	Clmp
16	0	2.976126661	0.518	0.148	0	0	Tagln
17	0	2.78539556896044	0.398	0.077	0	0	Arx
18	0	2.79445947759469	0.367	0.065	0	0	Srpx2
19	0	2.82544519640187	0.268	0.048	0	0	Cpz
20	0	2.91509795629452	0.263	0.044	0	0	Ngfr
21	0	3.08945405195125	0.267	0.051	0	0	Pdgfrl
22	0	2.86214755642493	0.245	0.044	0	0	Atp1a2
23	0	3.36003572233553	0.225	0.032	0	0	Syt1
24	0	3.35644793399668	0.223	0.031	0	0	Fmo1
25	0	2.93506959367747	0.231	0.045	0	0	Lbhd2
26	0	2.86901691570445	0.215	0.038	0	0	Lgi2
27	0	2.82476265569875	0.234	0.058	0	0	Mfap5
28	0	2.76875177092575	0.213	0.04	0	0	Pamr1
29	0	2.96751570754207	0.213	0.043	0	0	Pdlim3
30	0	2.9145367923261	0.189	0.037	0	0	Scube2
31	0	2.84358281171871	0.189	0.037	0	0	Rbp4
32	0	2.84123780530334	0.18	0.03	0	0	Tmem119
33	0	3.4148748025913	0.167	0.02	0	0	Gm266
34	0	2.93908374766845	0.174	0.03	0	0	Fgf2
35	0	2.91720566238336	0.164	0.025	0	0	Olfml1
36	0	2.98910177587018	0.161	0.025	0	0	C1qtnf2
37	0	2.88521932306886	0.17	0.042	0	0	Cxcl14
38	0	2.78759682007244	0.156	0.033	0	0	Adamtsl1
39	0	3.11895590129266	0.14	0.022	0	0	Vstm2b
40	0	2.81178564289269	0.144	0.035	0	0	Kcnb1
41	0	2.90659941875573	0.12	0.021	0	0	Osr1
42	0	2.92355900774316	0.113	0.017	0	0	Ms4a4d
43	0	3.02906742083018	0.113	0.018	0	0	Gm11627
44	0	3.31782965611328	0.108	0.016	0	0	Rtl3
45	0	3.78186602536424	0.104	0.012	0	0	Spon2
46	0	2.94703480761959	0.108	0.018	0	0	Col8a2
47	0	2.86361199474651	0.11	0.02	0	0	Gdf10
48	0	2.97826290403306	0.105	0.018	0	0	Cacna1g
49	0	3.19243552598543	0.103	0.017	0	0	Dpt

50	0	2.85638819285122	0.103	0.021	0	0	Insyn1
51	0	3.11488604316153	0.866	0.185	0	1	Col18a1
52	0	2.93796857543676	0.918	0.277	0	1	Itga6
53	0	2.85571082273629	0.832	0.2	0	1	Pcsk6
54	0	3.30666284144714	0.789	0.159	0	1	Aard
55	0	2.9264348507045	0.718	0.122	0	1	Gstm6
56	0	3.07367858182456	0.87	0.315	0	1	Kitl
57	0	2.71887078028641	0.971	0.428	0	1	Kctd14
58	0	2.70819432685353	0.742	0.22	0	1	Fndc5
59	0	2.8940177065442	0.642	0.144	0	1	Gm35533
60	0	2.84743261080066	0.649	0.163	0	1	Smad3
61	0	2.94228462903615	0.543	0.113	0	1	Arap2
62	0	3.49670480050383	0.491	0.067	0	1	Zg16
63	0	2.65233868287992	0.447	0.093	0	1	Mettl24
64	0	2.53500229266874	0.413	0.084	0	1	Krt79
65	0	2.87117842922767	0.413	0.095	0	1	1190002N15Rik
66	0	3.22187630531119	0.362	0.062	0	1	Gm37350
67	0	2.96531945202176	0.365	0.065	0	1	Angptl6
68	0	2.60660211322173	0.324	0.056	0	1	Dmrta1
69	0	4.14495158169718	0.284	0.025	0	1	Cntnap5a
70	0	3.37466667627804	0.257	0.027	0	1	Chst9
71	0	2.5702875750679	0.289	0.063	0	1	Fez1
72	0	3.03958847925378	0.259	0.039	0	1	Pla2g16
73	0	4.35979223242501	0.241	0.022	0	1	Col8a1
74	0	3.12028427451368	0.25	0.037	0	1	Gm5067
75	0	2.85642699720484	0.244	0.041	0	1	Rnf182
76	0	3.78423159386541	0.222	0.021	0	1	Cacna2d2
77	0	2.55688488961822	0.249	0.05	0	1	Tmem206
78	0	3.47358411231451	0.226	0.028	0	1	Ly6g6e
79	0	2.66661901396651	0.251	0.055	0	1	Bmp4
80	0	3.39073444635573	0.221	0.028	0	1	AA414768
81	0	3.1643049348521	0.204	0.029	0	1	Gm21168
82	0	2.6512630159867	0.186	0.03	0	1	Gldn
83	0	3.67441222633449	0.169	0.019	0	1	Hist3h2ba
84	0	2.79746537268358	0.166	0.027	0	1	Ccdc187
85	0	3.71572039958015	0.155	0.021	0	1	AC167229.1
86	0	3.17380349578103	0.154	0.025	0	1	Msx1
87	0	2.77250537680536	0.156	0.029	0	1	Lrrc4
88	0	3.36195927827286	0.149	0.022	0	1	Akr1c19
89	0	2.71085559718712	0.152	0.028	0	1	Calb1
90	0	2.89355012448968	0.145	0.021	0	1	Kif26b
91	0	3.76896679791295	0.137	0.015	0	1	5330431K02Rik
92	0	2.81393185674086	0.143	0.023	0	1	Rnf152
93	0	2.97620627197713	0.137	0.022	0	1	Tub
94	0	3.49677172916257	0.123	0.013	0	1	Hamp2
95	0	3.30356603870345	0.121	0.014	0	1	Gm15222
96	0	3.63261913654584	0.116	0.012	0	1	Tmem215
97	0	4.52645545531967	0.111	0.007	0	1	2810039B14Rik
98	0	2.53487704337181	0.111	0.019	0	1	Fam19a5
99	0	2.57548134245385	0.114	0.023	0	1	Coch
100	0	2.72793512125697	0.11	0.02	0	1	Snca

101	0	3.20799538530496	0.75	0.504	0	2	AY036118
102	0	1.07707460996458	0.955	0.724	0	2	mt-Co2
103	0	0.963220216205053	0.963	0.749	0	2	mt-Co3
104	0	1.08335901758775	0.969	0.791	0	2	mt-Atp6
105	0	2.27574745427757	0.512	0.389	0	2	Hbb-bs
106	0	2.05071098607454	0.984	0.987	0	2	Gm42418
107	0	2.31277130371773	0.56	0.558	0	2	Lars2
108	4.5969523269933e-270	0.952618299231247	0.1	0.335	2.61014953126679e-265	2	Znrd2
109	5.87541535221502e-213	1.18072335269662	0.063	0.235	3.33606083698769e-208	2	Snhg9
110	1.29838973840046e-200	0.885893352536927	0.044	0.193	7.37225693463783e-196	2	Prxl2b
111	9.27363952259332e-196	1.10014885906349	0.062	0.223	5.26557252092849e-191	2	Oga
112	4.61630551146414e-189	0.850606429656278	0.1	0.289	2.62113826940934e-184	2	Cables1
113	1.04714011492064e-171	0.910727184861304	0.049	0.186	5.94566157251939e-167	2	Tasor
114	1.34053926016874e-155	0.953230014531967	0.038	0.158	7.6115819192381e-151	2	Me3
115	2.06134396613161e-154	1.04600515855696	0.042	0.164	1.17043110396953e-149	2	Ano4
116	1.51608945024465e-151	1.05627136508234	0.095	0.254	8.60835589848914e-147	2	Hist1h4d
117	1.23185893794048e-150	0.983573418212483	0.038	0.155	6.99449504962606e-146	2	Hist1h4h
118	7.63225091624727e-147	0.951898520748165	0.048	0.17	4.3335920702452e-142	2	AC149090.1
119	8.97779195624222e-143	0.96837440141049	0.027	0.131	5.09759027275433e-138	2	Tasor2
120	1.83666301900709e-138	0.929713487099284	0.041	0.153	1.04285726219222e-133	2	Sema7a
121	6.57903267266729e-133	2.01319672435845	0.236	0.507	3.73557475154049e-128	2	Gm26917
122	7.94373208476711e-132	1.15799822137432	0.05	0.166	4.51045107773077e-127	2	Hist1h3e
123	3.24415498428224e-127	0.885244591789501	0.026	0.12	1.84203120007545e-122	2	Cyp2s1
124	1.1012650502313e-126	1.00077180982731	0.042	0.148	6.25298295521335e-122	2	Mctp1
125	1.36490259314974e-126	1.04033155758238	0.077	0.208	7.74991692390421e-122	2	Thbs4
126	2.75077062738901e-126	1.17098730510618	0.029	0.125	1.56188756223148e-121	2	Pwwp3a
127	1.2529018985493e-122	1.13755894164718	0.028	0.122	7.11397697996293e-118	2	Sel1l3
128	6.22693725647789e-121	1.16041216437114	0.033	0.129	3.53565497422815e-116	2	Nos2
129	9.05012334642113e-121	1.2081747368347	0.045	0.149	5.13866003609792e-116	2	Phykpl
130	3.50590802857105e-120	0.852313222480374	0.02	0.105	1.99065457862264e-115	2	Pfas
131	1.53438199392867e-118	0.916058420637057	0.02	0.105	8.712220961527e-114	2	Ccdc68
132	4.56634885787611e-118	1.10938231300719	0.158	0.342	2.59277288150205e-113	2	Hist1h1e
133	4.09951984362046e-117	0.970618579152079	0.021	0.105	2.3277073672077e-112	2	Large2
134	1.20825868703121e-116	1.01940934439446	0.022	0.107	6.86049282496323e-112	2	Dpysl5
135	4.79847390261127e-116	1.02757132543356	0.037	0.133	2.72457348190268e-111	2	Rapgef4
136	2.62517417102808e-115	2.41789719093227	0.308	0.251	1.49057389430974e-110	2	Hba-a1
137	3.13771096983272e-107	1.15491318496879	0.036	0.126	1.78159228867102e-102	2	Hist2h2ac
138	3.45318255938192e-102	1.42649906728955	0.509	0.617	1.96071705721705e-97	2	mt-Nd4l
139	1.86908614598549e-93	1.04437558501528	0.038	0.121	1.06126711369056e-88	2	Slc38a3
140	1.0227157259371e-86	1.38594025382049	0.076	0.18	5.80697989187085e-82	2	C230038L03Rik
141	6.40754377177616e-79	1.2827806320609	0.245	0.476	3.6382033536145e-74	2	Gm47283
142	1.0012579322361e-77	1.43394297688706	0.046	0.124	5.68514253923656e-73	2	Hist1h1d
143	1.65264793620389e-63	1.47083800911706	0.042	0.108	9.3837349817657e-59	2	Bc1
144	7.57792328666426e-62	0.994906309134961	0.103	0.201	4.30274484216796e-57	2	Slc38a5
145	5.38799222790197e-57	2.48852503550402	0.248	0.216	3.05930198700274e-52	2	Hba-a2
146	3.49663874686957e-38	1.71026532403006	0.057	0.112	1.98539148047254e-33	2	Hist1h3c
147	9.13031771794064e-36	1.2188242983397	0.194	0.323	5.18419440024669e-31	2	Slc18a2
148	7.80253502162096e-20	0.875263723182004	0.178	0.263	4.43027938527638e-15	2	Amh
149	1.04091398938068e-15	1.25055011679856	0.207	0.306	5.91030963170347e-11	2	Hist1h2ae
150	0.000482272358142054	0.91428713729927	0.347	0.529	1	2	Atp5md
151	0	2.56159402899086	0.957	0.262	0	3	Hsd17b1

152	0	2.48701233308201	0.814	0.16	0	3	Car14
153	0	2.94253212508281	0.809	0.166	0	3	Nap1l5
154	0	2.25415496969556	0.809	0.174	0	3	Apoa4
155	0	2.06567421335205	0.898	0.267	0	3	Fam13a
156	0	2.04365065941753	0.751	0.187	0	3	Cnmd
157	0	2.35523697047158	0.706	0.144	0	3	Tox2
158	0	2.26879825609356	0.706	0.144	0	3	Slc38a5
159	0	1.99229716186529	0.765	0.23	0	3	Tmsb15b2
160	0	2.24343381992068	0.631	0.128	0	3	Rasl10a
161	0	3.57069095777522	0.673	0.195	0	3	Inhba
162	0	1.97825442896832	0.622	0.146	0	3	Mpped2
163	0	2.5366825043299	0.712	0.237	0	3	Inhbb
164	0	2.29587392380409	0.912	0.454	0	3	Prkar2b
165	0	1.96601792965525	0.994	0.56	0	3	Gja1
166	0	2.57065514674569	0.485	0.076	0	3	Dnajb13
167	0	2.31229643078756	0.485	0.092	0	3	Ihh
168	0	2.00853005429587	0.521	0.129	0	3	Fam81a
169	0	2.47149077	0.497	0.117	0	3	Nppc
170	0	2.3929596239462	0.999	0.631	0	3	Inha
171	0	2.16481294683025	0.416	0.082	0	3	Crabp2
172	0	1.95918998035435	0.408	0.084	0	3	Slc38a3
173	0	2.1531006429409	0.401	0.083	0	3	Dhh
174	0	2.20693565087018	0.371	0.065	0	3	Sema3g
175	0	1.93292315585001	0.386	0.086	0	3	Pparg
176	0	2.32764061503034	0.354	0.062	0	3	Cbs
177	0	1.98139837928026	0.354	0.08	0	3	Lrrc2
178	0	2.23654984483612	0.289	0.055	0	3	Gm48882
179	0	2.37018783988557	0.276	0.05	0	3	Fshr
180	0	2.19295061204476	0.262	0.052	0	3	Srcin1
181	0	2.08572216857344	0.258	0.049	0	3	Gulo
182	0	1.98842225090987	0.305	0.096	0	3	Masp1
183	0	2.03061987069314	0.252	0.046	0	3	Gm9961
184	0	2.07050374583052	0.298	0.095	0	3	Mro
185	0	2.05936841349077	0.244	0.05	0	3	Tmem52
186	0	2.09160244729322	0.236	0.046	0	3	Apoc3
187	0	2.39448594231138	0.21	0.032	0	3	Arhgap27os2
188	0	2.17475711295415	0.204	0.031	0	3	Fam183b
189	0	2.11735143426974	0.208	0.037	0	3	Rgs13
190	0	2.25647166129621	0.207	0.036	0	3	Otor
191	0	2.20995872672336	0.197	0.036	0	3	1700007K13Rik
192	0	2.66130611322253	0.179	0.03	0	3	Smim10l2a
193	0	3.43974837152228	0.167	0.022	0	3	Cyp19a1
194	0	2.54962474155863	0.166	0.025	0	3	Drd4
195	0	2.15648975943381	0.174	0.033	0	3	Creb3l3
196	0	2.08798823096666	0.177	0.038	0	3	Prrt4
197	0	3.48412135117142	0.156	0.02	0	3	Grem1
198	0	2.19843304283949	0.128	0.025	0	3	Gm4926
199	1.07130516592032e-281	2.0823518539771	0.108	0.02	6.08287073209559e-277	3	Lrrtm3
200	1.07213798131813e-238	1.94152299166584	0.113	0.025	6.08759945792435e-234	3	Icam4
201	0	2.36149454315288	0.888	0.158	0	4	Cdca8
202	0	2.21571890449769	0.894	0.165	0	4	Mki67

203	0	2.47949852962353	0.851	0.134	0	4	Tpx2
204	0	2.2594239073138	0.89	0.176	0	4	Cenpf
205	0	2.63426342293059	0.892	0.2	0	4	Ube2c
206	0	2.58715430007096	0.763	0.091	0	4	Pbk
207	0	2.30163281015064	0.774	0.104	0	4	Ccna2
208	0	2.21756417191452	0.785	0.122	0	4	Cdca3
209	0	2.5217130735068	0.772	0.121	0	4	Cdc20
210	0	2.28906343560019	0.779	0.142	0	4	Ccnb2
211	0	2.39172312197927	0.707	0.081	0	4	Hmmr
212	0	2.17999742071777	0.82	0.205	0	4	Amh
213	0	2.42462809562356	0.692	0.085	0	4	Ccnb1
214	0	2.16588269632897	0.777	0.172	0	4	Cenpa
215	0	2.27042812907716	0.833	0.249	0	4	Hist1h2ap
216	0	2.1760470961094	0.663	0.084	0	4	Kif11
217	0	2.24345287390414	0.663	0.089	0	4	Bub1b
218	0	2.31615831609091	0.639	0.074	0	4	Aurkb
219	0	2.36865206415213	0.633	0.073	0	4	Bub1
220	0	2.19234183349905	0.646	0.088	0	4	Mis18bp1
221	0	2.15745432024999	0.654	0.097	0	4	Kif22
222	0	2.38822051534009	0.621	0.075	0	4	Sgo2a
223	0	2.21727909796433	0.61	0.081	0	4	Aspm
224	0	2.31331154373457	0.76	0.235	0	4	Inhbb
225	0	2.37963415293834	0.546	0.062	0	4	Aurka
226	0	2.6193234351555	0.539	0.055	0	4	Plk1
227	0	2.23297077058285	0.566	0.086	0	4	Ihh
228	0	2.21461653263742	0.547	0.068	0	4	Hist1h3c
229	0	2.1848763942486	0.539	0.068	0	4	Dlgap5
230	0	2.15845027721833	0.538	0.08	0	4	Cdkn3
231	0	2.29079245759789	0.499	0.056	0	4	Hist1h2ab
232	0	2.25057563585556	0.495	0.057	0	4	Cdca2
233	0	2.39609414198249	0.489	0.052	0	4	Kif2c
234	0	2.44509257170082	0.46	0.043	0	4	Cep55
235	0	2.26633958653489	0.464	0.06	0	4	Cip2a
236	0	2.5078149859998	0.437	0.054	0	4	Ank1
237	0	2.20387146487936	0.417	0.041	0	4	Depdc1a
238	0	2.67029207642273	0.393	0.034	0	4	Nek2
239	0	2.23521419839397	0.372	0.043	0	4	Hist1h2bj
240	0	2.18795435224424	0.342	0.039	0	4	Gm9961
241	0	2.3470720994575	0.297	0.03	0	4	Troap
242	0	2.25131226400974	0.282	0.021	0	4	Hist1h2af
243	0	2.19200851073741	0.272	0.027	0	4	Hist1h2bm
244	0	2.60289501108456	0.258	0.021	0	4	Arhgef39
245	0	2.28972941300635	0.266	0.032	0	4	Otor
246	0	2.31824127339312	0.236	0.024	0	4	Sapcd2
247	0	2.23423398584267	0.215	0.018	0	4	Kif18b
248	0	2.23038334242241	0.165	0.016	0	4	Hist1h2bh
249	0	2.41572979348573	0.108	0.008	0	4	Hist1h2ai
250	0	2.21246760119698	0.101	0.009	0	4	Hist1h2bk
251	0	3.4426288330306	0.873	0.198	0	5	Pclaf
252	0	3.17564123921913	0.826	0.182	0	5	Mki67
253	0	3.13463112923331	0.832	0.228	0	5	Top2a

254	0	2.81291543626778	0.719	0.156	0	5	Tpx2
255	0	3.26923190920046	0.66	0.118	0	5	Knl1
256	0	3.15532525445377	0.644	0.123	0	5	Nusap1
257	0	2.94026698781949	0.655	0.142	0	5	Cdca3
258	0	3.01835686675709	0.63	0.128	0	5	Spc25
259	0	3.51318950372092	0.587	0.085	0	5	Tk1
260	0	4.08908334359592	0.535	0.054	0	5	Mxd3
261	0	2.99251289298326	0.616	0.138	0	5	Kif23
262	0	2.83978481503614	0.623	0.151	0	5	Cenpe
263	0	3.126769578	0.571	0.101	0	5	Ckap2l
264	0	2.90134904353517	0.56	0.11	0	5	Kif15
265	0	3.07536503240319	0.539	0.095	0	5	Esco2
266	0	2.95977420299134	0.537	0.103	0	5	Hmmr
267	0	2.82793000154721	0.547	0.117	0	5	Clspn
268	0	2.90534255205633	0.532	0.103	0	5	Kif11
269	0	3.35077814846694	0.524	0.101	0	5	Ckap2
270	0	3.17941522412377	0.503	0.084	0	5	Anln
271	0	3.17768860747486	0.494	0.079	0	5	Shcbp1
272	0	3.03189029021519	0.507	0.095	0	5	Rad51ap1
273	0	3.06607890329318	0.475	0.072	0	5	Fam111a
274	0	2.83465126434674	0.489	0.099	0	5	Sgo1
275	0	2.84843504357462	0.492	0.108	0	5	Mis18bp1
276	0	2.82754497809435	0.495	0.111	0	5	Ncapg2
277	0	2.92952448480761	0.468	0.094	0	5	Sgo2a
278	0	2.85043573540538	0.462	0.089	0	5	Ndc80
279	0	3.65970190328554	0.419	0.05	0	5	Neil3
280	0	3.35682444648077	0.416	0.061	0	5	Nuf2
281	0	3.19747487208434	0.422	0.07	0	5	Pimreg
282	0	2.79923190018026	0.418	0.08	0	5	Cdkn2c
283	0	3.36758906441734	0.396	0.063	0	5	Tcf19
284	0	2.90400586359076	0.394	0.073	0	5	Melk
285	0	3.12161646896958	0.38	0.06	0	5	Ska1
286	0	2.84052395027396	0.391	0.076	0	5	Cit
287	0	3.09827826404863	0.38	0.067	0	5	Kif20a
288	0	3.2848738537871	0.355	0.052	0	5	Depdc1a
289	0	2.87309186789717	0.37	0.073	0	5	Cdca2
290	0	2.80005612402417	0.338	0.077	0	5	Slfn9
291	0	2.83756704889825	0.308	0.058	0	5	Psrc1
292	0	3.16111372018646	0.234	0.036	0	5	E2f8
293	0	3.13756947252496	0.229	0.038	0	5	E2f7
294	0	2.96539750757019	0.181	0.021	0	5	Cgas
295	0	2.91680999053449	0.177	0.029	0	5	Haspin
296	0	3.12933069834421	0.137	0.016	0	5	Neurl1b
297	0	4.34975245100398	0.131	0.011	0	5	2700099C18Rik
298	0	3.02393313407764	0.142	0.022	0	5	Ankle1
299	0	3.43695093953696	0.101	0.011	0	5	Xkr5
300	1.2314886413109e-250	2.94960795347824	0.101	0.016	6.99239250536331e-246	5	Rtkn2
301	0	3.07507236075074	0.914	0.281	0	6	Prss23
302	0	2.26521479705365	0.914	0.337	0	6	Sema5a
303	0	2.56870508215642	0.838	0.264	0	6	Crim1
304	0	2.89115734011917	0.954	0.392	0	6	Grb14

305	0	2.79107909736786	0.755	0.195	0	6	Pik3ip1
306	0	2.88879997898163	0.694	0.137	0	6	Foxp2
307	0	2.24215658091773	0.935	0.41	0	6	Foxo1
308	0	2.22864304609268	0.697	0.198	0	6	Ccn2
309	0	3.28584435323662	0.566	0.073	0	6	Omd
310	0	2.17464873134276	0.665	0.179	0	6	Bmpr1b
311	0	2.16146322533052	0.668	0.194	0	6	Zfp385b
312	0	2.39518554880731	0.607	0.178	0	6	Col11a1
313	0	3.3158612999176	0.473	0.071	0	6	Ntn4
314	0	2.54462651944357	0.465	0.108	0	6	Fhl1
315	0	2.57061486587056	0.39	0.06	0	6	Csmd1
316	0	3.26996480462178	0.404	0.075	0	6	Adamts5
317	0	2.50087740764063	0.386	0.06	0	6	Ccdc3
318	0	2.61271781165814	0.371	0.067	0	6	Itga2
319	0	2.4409372136132	0.334	0.059	0	6	Vpreb3
320	0	3.3153916092135	0.307	0.035	0	6	Krt20
321	0	2.51080397619447	0.325	0.064	0	6	Fhdc1
322	0	3.15333880996365	0.292	0.034	0	6	4933431K23Rik
323	0	3.15124060463299	0.294	0.037	0	6	Rgs13
324	0	2.45667802239133	0.308	0.057	0	6	Asb4
325	0	2.12963793866361	0.306	0.061	0	6	Pde7b
326	0	3.40887486509523	0.247	0.023	0	6	Mug2
327	0	2.65740447155853	0.241	0.037	0	6	Kcnq5
328	0	2.62114903991538	0.227	0.03	0	6	Fgd5
329	0	2.67528722989838	0.222	0.034	0	6	Sgpp2
330	0	2.13645530011772	0.225	0.047	0	6	Klhl31
331	0	2.19178333002688	0.22	0.043	0	6	Reln
332	0	3.23897405233346	0.193	0.02	0	6	Gm42303
333	0	3.23793722884199	0.19	0.019	0	6	Itih2
334	0	2.22189073920233	0.211	0.042	0	6	Sema3b
335	0	2.27711241571472	0.2	0.036	0	6	Atoh8
336	0	3.41569497751653	0.181	0.018	0	6	Tdgf1
337	0	2.35069672441204	0.203	0.044	0	6	Nr0b1
338	0	2.49272466331919	0.193	0.041	0	6	1110032F04Rik
339	0	4.53090991633479	0.128	0.009	0	6	Prg4
340	0	2.92234849172873	0.136	0.02	0	6	Gm15866
341	0	2.89722507343399	0.112	0.012	0	6	Smim5
342	0	4.24971246532395	0.102	0.006	0	6	Cntf
343	6.78774897538919e-298	2.24462734793156	0.161	0.03	3.85408386822598e-293	6	Rai2
344	2.61402380435508e-292	2.74093548233939	0.138	0.024	1.48424271611281e-287	6	Chrdl1
345	2.62364938983679e-281	2.27167940074391	0.156	0.03	1.48970812354933e-276	6	Scg5
346	8.36890233349031e-277	2.13192077681487	0.13	0.022	4.7518627449558e-272	6	Galnt15
347	1.4564416141627e-182	2.4679358013089	0.119	0.026	8.26967548521583e-178	6	Tnnc1
348	1.27256252412009e-179	2.17975920364141	0.113	0.024	7.22561001195388e-175	6	Dkkl1
349	2.88513135083497e-179	2.50360707573573	0.107	0.022	1.6381775810041e-174	6	Lrrtm3
350	1.17631966278923e-145	2.13165431625262	0.17	0.056	6.67914304531726e-141	6	Neto2
351	0	3.40145117475982	0.785	0.104	0	7	Aldh1a2
352	0	4.12730902755345	0.743	0.084	0	7	Gng13
353	0	3.94610969681401	0.774	0.121	0	7	Aldh1b1
354	0	3.14921341472962	0.788	0.171	0	7	Irx3
355	0	3.54066390799728	0.751	0.15	0	7	Prcd

356	0	3.19233059633508	0.741	0.14	0	7	Krt18
357	0	3.28751894112019	0.698	0.129	0	7	Ano1
358	0	3.36622385142973	0.678	0.126	0	7	Gm45889
359	0	3.25442640408544	0.656	0.116	0	7	Cgn
360	0	3.26948568908964	0.577	0.086	0	7	Fam129a
361	0	3.72470250592233	0.541	0.066	0	7	Smpx
362	0	4.04687129114908	0.517	0.054	0	7	Tnni1
363	0	3.96579361201039	0.508	0.049	0	7	Crygb
364	0	3.85984677615379	0.523	0.073	0	7	Gsta3
365	0	5.3920625561154	0.459	0.022	0	7	U90926
366	0	3.96182216396752	0.439	0.043	0	7	Adora1
367	0	3.32965860364739	0.455	0.064	0	7	Apoc1
368	0	3.20220931422154	0.457	0.071	0	7	Stom
369	0	3.69328995507979	0.461	0.077	0	7	Cox7a1
370	0	3.19707795085695	0.597	0.214	0	7	Cst12
371	0	3.49277451977286	0.423	0.075	0	7	Clec2l
372	0	3.55843423997606	0.363	0.031	0	7	Lgr5
373	0	3.63127670415248	0.371	0.04	0	7	Dtx1
374	0	3.99555855513851	0.36	0.029	0	7	Acsm3
375	0	3.52447585873149	0.348	0.043	0	7	Rec8
376	0	3.61399896942822	0.289	0.035	0	7	Kcnq1
377	0	3.25090512594791	0.276	0.041	0	7	Pde8b
378	0	4.0411665196879	0.25	0.021	0	7	Zfp459
379	0	4.26394253729664	0.222	0.015	0	7	AC122818.3
380	0	4.13163005578794	0.22	0.019	0	7	Ly6d
381	0	4.15416299600208	0.213	0.021	0	7	Cpa2
382	0	3.20730627656715	0.224	0.033	0	7	Csgalnact1
383	0	3.52218884188335	0.21	0.024	0	7	Myzap
384	0	3.94682981834635	0.202	0.017	0	7	Slc16a4
385	0	4.03048244597451	0.199	0.016	0	7	Wfdc6a
386	0	4.35758823759608	0.195	0.014	0	7	AI661453
387	0	3.66746425086951	0.195	0.024	0	7	Nqo1
388	0	4.47244989913211	0.173	0.009	0	7	3100003L05Rik
389	0	4.36376091354604	0.169	0.011	0	7	Gm17660
390	0	3.54299646029053	0.176	0.019	0	7	Cfap54
391	0	4.24183377429998	0.166	0.012	0	7	Necab3
392	0	4.96649795011519	0.161	0.009	0	7	Cst9
393	0	4.29100736636226	0.148	0.009	0	7	Clca3a2
394	0	4.95429035327126	0.134	0.007	0	7	1700003F12Rik
395	0	5.82291393183054	0.129	0.004	0	7	Mhrt
396	0	3.92406175859707	0.129	0.012	0	7	Crygc
397	0	4.21033270431611	0.128	0.012	0	7	Gata1
398	0	3.40277396010631	0.119	0.01	0	7	Svs5
399	0	3.36100510627418	0.109	0.012	0	7	Matk
400	5.34443324253344e-257	3.4408235427149	0.101	0.013	3.03456919511049e-252	7	Slc30a2
401	0	4.71882284656811	0.981	0.2	0	8	Cyp11a1
402	0	5.14072025789068	0.865	0.09	0	8	Mrap
403	0	4.66885387625712	0.894	0.187	0	8	Mgarp
404	0	5.3365485194051	0.713	0.039	0	8	Cemip
405	0	4.56836645794123	0.777	0.11	0	8	Slc25a30
406	0	5.25573735616211	0.739	0.08	0	8	Sfrp4

407	0	4.6879758369424	0.721	0.071	0	8	Tinagl1
408	0	5.58869721193826	0.676	0.027	0	8	Runx2
409	0	4.30043806890031	0.662	0.051	0	8	Mgst2
410	0	4.32084079854429	0.617	0.048	0	8	Lhcgr
411	0	4.84587589835173	0.447	0.02	0	8	Jakmip3
412	0	5.39972067192815	0.43	0.02	0	8	Fabp4
413	0	4.79434699755392	0.398	0.012	0	8	Tmem71
414	0	4.40004171628447	0.403	0.021	0	8	Bves
415	0	5.54078093328069	0.379	0.009	0	8	Slc12a8
416	0	5.09142170945008	0.354	0.009	0	8	Ccn5
417	0	5.9668597904585	0.351	0.006	0	8	Plin5
418	0	6.42443954575694	0.334	0.007	0	8	Bhmt
419	0	4.76171674589594	0.355	0.03	0	8	Ptgfr
420	0	4.8061065096772	0.323	0.013	0	8	Oxtr
421	0	4.70145064196618	0.313	0.014	0	8	Cabp1
422	0	5.36045195644973	0.304	0.007	0	8	D630023F18Rik
423	0	4.30120505858206	0.297	0.01	0	8	Rab27a
424	0	5.48303464096454	0.295	0.009	0	8	Timp4
425	0	4.4392115122307	0.291	0.019	0	8	Ptgs1
426	0	4.99523638784173	0.27	0.008	0	8	Asgr1
427	0	7.68916115149623	0.249	0.002	0	8	Cldn18
428	0	4.45424972396832	0.256	0.01	0	8	Nr1h4
429	0	4.46156502827581	0.249	0.015	0	8	Apln
430	0	4.71755349294592	0.265	0.05	0	8	Akr1c18
431	0	4.64892383208127	0.228	0.015	0	8	Edn2
432	0	4.43814149489009	0.213	0.011	0	8	Prkg2
433	0	5.61725001523982	0.2	0.003	0	8	Ssu2
434	0	4.99750827428942	0.188	0.003	0	8	Plin4
435	0	4.81095375868651	0.176	0.004	0	8	Gm31135
436	0	4.52731459031464	0.178	0.006	0	8	Usp43
437	0	4.77389265689484	0.162	0.005	0	8	Txndc2
438	0	5.13559040937213	0.159	0.003	0	8	Kl
439	0	5.34203914949182	0.159	0.003	0	8	Gm50114
440	0	4.68853530447153	0.16	0.005	0	8	F730043M19Rik
441	0	4.33167142812133	0.156	0.009	0	8	Nrcam
442	0	4.59827731840195	0.148	0.005	0	8	Plekhg4
443	0	5.24618549097433	0.145	0.004	0	8	Mmel1
444	0	4.83121916500022	0.143	0.005	0	8	Slc16a11
445	0	4.86881048266974	0.143	0.005	0	8	Wnt10b
446	0	5.5205936387115	0.137	0.002	0	8	Xpnpep2
447	0	5.72459694768266	0.139	0.007	0	8	Onecut2
448	0	5.83698510388099	0.117	0.002	0	8	Etos1
449	0	4.52140154202454	0.114	0.005	0	8	Apol6
450	0	6.30634255880967	0.102	0.001	0	8	Aldob
451	3.58923294333638e-176	1.21894238840851	0.574	0.264	2.0379664652264e-171	9	Aes
452	1.42480390733227e-169	1.22929930270986	0.323	0.095	8.0900365858326e-165	9	Hyi
453	3.28379182649378e-147	1.21892110091877	0.322	0.104	1.86453699908317e-142	9	Gm43362
454	4.99952036315719e-145	1.41421853413971	0.246	0.068	2.83872766220065e-140	9	Gm19327
455	2.47364504839536e-143	1.31208004556345	0.262	0.076	1.40453565847889e-138	9	Ppp4r1l-ps
456	6.76497067646627e-143	1.16865482484446	0.223	0.058	3.84115035009755e-138	9	3110039I08Rik
457	1.90217384679308e-138	1.15634442734065	0.362	0.13	1.08005431020911e-133	9	Tgfb2

458	1.67502582521967e-125	1.15694037923944	0.226	0.064	9.51079663559726e-121	9	Gm5067
459	4.17376776324295e-122	1.20803576713344	0.239	0.072	2.36986533596935e-117	9	Gm49599
460	7.36764842399203e-122	1.40965306907419	0.197	0.053	4.18335077514267e-117	9	AC121821.1
461	1.36201826116229e-118	1.25841180758619	0.226	0.067	7.7335396868795e-114	9	Rps11-ps1
462	4.41513486711466e-110	1.21150966863662	0.195	0.055	2.50691357754771e-105	9	Gm42992
463	6.52907306261881e-104	1.22737906299496	0.191	0.056	3.70720768495496e-99	9	Ikzf4
464	4.98692138907912e-102	1.2057063662259	0.171	0.047	2.83157396471912e-97	9	Gm37238
465	2.11987349251809e-100	1.15243107838539	0.18	0.051	1.20366416905177e-95	9	Gm21168
466	2.40552545573415e-98	1.47040260105936	0.148	0.038	1.36585735376585e-93	9	9930017N22Rik
467	3.75369955385496e-98	1.1414937129084	0.173	0.049	2.13135060667884e-93	9	Iqcn
468	1.15926051947998e-94	1.47926152963891	0.134	0.033	6.5822812296073e-90	9	Gm45153
469	4.66950690713141e-93	1.32206592500245	0.162	0.046	2.65134602186921e-88	9	C130089K02Rik
470	9.39523766013661e-92	1.26405797576424	0.143	0.037	5.33461594342557e-87	9	Gm42993
471	1.26302468968866e-90	1.24777768255324	0.175	0.053	7.17145418805223e-86	9	Rec8
472	2.11373983235885e-89	1.14416674993631	0.187	0.058	1.20018147681335e-84	9	Fam213a
473	1.36562819462266e-88	1.38009600777891	0.14	0.037	7.75403688906744e-84	9	Gm43359
474	2.18551813199449e-88	1.18345530869311	0.189	0.06	1.24093719534647e-83	9	6820402A03Rik
475	1.04278884237175e-87	1.36528230682702	0.1	0.021	5.92095504698677e-83	9	Gm43022
476	3.96242557552746e-86	1.15940299749312	0.156	0.045	2.24986524178449e-81	9	D3Ertd254e
477	1.00609354123961e-84	1.20564708364458	0.169	0.051	5.71259912715851e-80	9	Mir703
478	8.83230754347406e-81	1.23188459846198	0.141	0.04	5.01498422318457e-76	9	Zim1
479	1.44911079444846e-77	1.42065694413635	0.111	0.028	8.22805109087836e-73	9	Gm37105
480	7.9406709636112e-77	1.18629817207359	0.133	0.037	4.50871297313844e-72	9	Zfp862-ps
481	1.03971881239232e-76	1.23567275731875	0.128	0.035	5.90352341676357e-72	9	Gm8730
482	6.16983512486717e-75	1.28041454719176	0.139	0.041	3.50323238389958e-70	9	Gm43484
483	2.33176707277899e-74	1.3949363988328	0.116	0.03	1.32397734392391e-69	9	5330431K02Rik
484	3.87592087829963e-74	1.19824752073375	0.123	0.034	2.20074787469853e-69	9	B330016D10Rik
485	7.3497888403928e-70	1.3422848423105	0.104	0.027	4.17321010357503e-65	9	Gm37258
486	1.44568826763343e-68	1.25631495714531	0.129	0.038	8.20861798362263e-64	9	Hist3h2ba
487	5.00545893758408e-68	1.18617314352528	0.131	0.039	2.84209958476024e-63	9	A930004J17Rik
488	4.11146899343377e-67	1.23263606205944	0.103	0.027	2.33449209447169e-62	9	Rps10-ps2
489	1.27916549446721e-65	1.3134733348437	0.11	0.03	7.26310167758481e-61	9	Gpx4-ps2
490	2.84655822059994e-65	1.1548990898769	0.11	0.03	1.61627575765664e-60	9	Fam19a5
491	1.66637524384623e-63	1.19406448578194	0.13	0.041	9.46167863455887e-59	9	Acsm3
492	3.87111593987503e-63	1.14458995158676	0.124	0.037	2.19801963066104e-58	9	Gm40309
493	7.89164078303046e-62	1.15927587892051	0.106	0.029	4.48087363660469e-57	9	C230071H17Rik
494	1.600220300579e-61	1.31056615600654	0.102	0.028	9.08605086668757e-57	9	Cnr1
495	2.76459742106389e-61	1.13242880288104	0.123	0.038	1.56973841568008e-56	9	Gm15920
496	8.58125048169702e-61	1.1872428353818	0.105	0.03	4.87243402350757e-56	9	Gm49338
497	7.86174491999309e-57	1.14732851185729	0.123	0.039	4.46389876557208e-52	9	Gm10033
498	6.4123329542142e-47	1.19633405368002	0.119	0.042	3.64092265140282e-42	9	Msx1
499	6.15222128085069e-42	1.17126932699512	0.111	0.04	3.49323124326702e-37	9	Scara5
500	8.90447498922508e-37	1.19269220032469	0.101	0.037	5.055960898882e-32	9	Egfl6
501	0	7.848086979	0.798	0.011	0	10	Cldn5
502	0	6.82743976354886	0.802	0.018	0	10	Ctla2a
503	0	6.71238113082645	0.779	0.022	0	10	Cd93
504	0	7.15768725231879	0.775	0.018	0	10	Pecam1
505	0	6.66139829310977	0.758	0.02	0	10	Ecscr
506	0	7.17656149500853	0.698	0.009	0	10	Rasip1
507	0	7.96388263228667	0.694	0.007	0	10	Flt1
508	0	8.00902699400715	0.646	0.004	0	10	Tie1

509	0	6.91390802678994	0.639	0.008	0	10	Icam2
510	0	7.74656470410155	0.603	0.005	0	10	Ly6c1
511	0	6.76665262324973	0.607	0.013	0	10	Ptprb
512	0	7.81404983198118	0.596	0.003	0	10	Myct1
513	0	8.73716612042176	0.575	0.004	0	10	Gpihbp1
514	0	6.39877811366542	0.576	0.011	0	10	Afap1l1
515	0	7.22625762049158	0.507	0.005	0	10	Robo4
516	0	7.76806347843266	0.493	0.003	0	10	Cyyr1
517	0	7.06933986331235	0.493	0.006	0	10	Grrp1
518	0	6.47425584016226	0.491	0.005	0	10	Mfng
519	0	7.90359831554499	0.465	0.002	0	10	Adgrl4
520	0	6.42075926423403	0.468	0.007	0	10	Prex2
521	0	6.48523369094348	0.451	0.007	0	10	Ushbp1
522	0	7.46997288726827	0.433	0.003	0	10	Sox17
523	0	6.66772945913553	0.427	0.004	0	10	Bcl6b
524	0	7.87557086771584	0.42	0.002	0	10	Mecom
525	0	6.54614792787936	0.425	0.007	0	10	Erg
526	0	7.58560733501154	0.405	0.002	0	10	Fam167b
527	0	7.10566063699444	0.369	0.006	0	10	Flt4
528	0	6.52074747964257	0.349	0.005	0	10	Cd109
529	0	7.53740272981723	0.342	0.002	0	10	Arhgef15
530	0	7.25192214551202	0.335	0.002	0	10	Sox7
531	0	6.90195142473572	0.335	0.005	0	10	Plvap
532	0	7.16636958446735	0.331	0.002	0	10	Dll4
533	0	6.55502928131922	0.279	0.003	0	10	Klhl4
534	0	8.16408475964718	0.229	0	0	10	Gm32688
535	0	7.49401770485471	0.225	0.001	0	10	Ccm2l
536	0	7.39189874634833	0.224	0.001	0	10	Nxpe2
537	0	6.397533834	0.221	0.002	0	10	Zfp366
538	0	7.22837671675041	0.22	0.003	0	10	Vwf
539	0	7.33101581663639	0.208	0.002	0	10	Wscd1
540	0	6.49740241895135	0.19	0.002	0	10	Lhx6
541	0	7.42081668212463	0.171	0.001	0	10	Ces2e
542	0	7.05976407664011	0.154	0.001	0	10	Gria2
543	0	6.90607537665672	0.128	0.001	0	10	Ankrd2
544	0	6.79383452475002	0.127	0.001	0	10	Prnd
545	0	7.06084935198284	0.125	0.001	0	10	Syt15
546	0	7.78485081469225	0.124	0.001	0	10	Gm525
547	0	6.44566518203616	0.123	0.002	0	10	Cd300lg
548	0	6.92609810778693	0.119	0.001	0	10	Gm11730
549	0	7.72222488313125	0.114	0.001	0	10	Depp1
550	0	7.5408830651432	0.106	0	0	10	Tmem252
551	0	3.22676532224676	0.83	0.144	0	11	Prlr
552	0	3.83765921879455	0.887	0.21	0	11	Cyp11a1
553	0	3.58921959899243	0.826	0.152	0	11	Smoc2
554	0	3.1808923390523	0.932	0.272	0	11	Acsbg1
555	0	3.0297875691041	0.843	0.195	0	11	Mgarp
556	0	4.25530910349283	0.748	0.109	0	11	Prxl2a
557	0	3.18355378395612	0.956	0.354	0	11	Aldh1a1
558	0	2.88665248169496	0.925	0.343	0	11	Me1
559	0	3.83407559904843	0.633	0.054	0	11	Lhcgr

560	0	2.86858721507688	0.695	0.13	0		11	Osr2
561	0	3.21730828402961	0.942	0.4	0		11	Hsd3b1
562	0	4.3421432473404	0.559	0.058	0		11	Hao2
563	0	3.24703882039075	0.582	0.103	0		11	Star
564	0	3.39833272891441	0.542	0.069	0		11	E330017L17Rik
565	0	3.80174677540178	0.538	0.066	0		11	Ephx1
566	0	3.1683297110355	0.513	0.061	0		11	Dtx4
567	0	6.80224382793337	0.462	0.033	0		11	Cyp17a1
568	0	2.9626591680535	0.978	0.551	0		11	Fdx1
569	0	5.19777377786208	0.44	0.018	0		11	Gsta2
570	0	2.85887175063429	0.487	0.089	0		11	Cst8
571	0	2.8613533363893	0.46	0.07	0		11	Tulp2
572	0	4.17745776677253	0.45	0.061	0		11	Stc1
573	0	4.49595270698292	0.424	0.037	0		11	Sct
574	0	3.01833403300374	0.528	0.155	0		11	Fads2
575	0	3.26479386357386	0.431	0.066	0		11	Srd5a1
576	0	4.73582129609984	0.373	0.013	0		11	Plin1
577	0	3.20658412627834	0.385	0.043	0		11	C2
578	0	6.34276798742188	0.342	0.006	0		11	Serpina5
579	0	2.95116512799468	0.395	0.059	0		11	Lipg
580	0	3.77609857539063	0.332	0.021	0		11	Sez6l2
581	0	3.09699015643822	0.361	0.052	0		11	Iqgap2
582	0	3.23696724324634	0.331	0.03	0		11	Efhd1
583	0	4.12905433019828	0.281	0.012	0		11	Slc45a3
584	0	4.53642428783415	0.276	0.017	0		11	Bpifb4
585	0	3.86713512712215	0.278	0.041	0		11	Abcb1b
586	0	4.12881041505055	0.245	0.018	0		11	AU021092
587	0	3.23992516871174	0.24	0.018	0		11	Oxtr
588	0	2.8760697572797	0.256	0.036	0		11	Pcx
589	0	3.78639583732561	0.228	0.017	0		11	Nckap5
590	0	4.87831644348889	0.202	0.01	0		11	Sftpc
591	0	4.9714082046602	0.2	0.011	0		11	Adh1
592	0	3.14705909310064	0.202	0.019	0		11	Tspan11
593	0	4.69882826589147	0.174	0.006	0		11	Serpina3c
594	0	3.38902470429003	0.166	0.013	0		11	Slc30a2
595	0	5.74663038454522	0.15	0.003	0		11	Bpifb5
596	0	5.96341085239508	0.136	0.002	0		11	Mgat4c
597	0	3.94771185599763	0.14	0.009	0		11	Cdk18
598	0	5.31739123140627	0.112	0.002	0		11	Stk32a
599	0	6.89592777571207	0.11	0.001	0		11	Insl3
600	3.17266317978228e-137	3.49306278567333	0.529	0.241	1.80143815348038e-132		11	Gpx3
601	0	7.34300584267945	0.967	0.024	0		12	Krt19
602	0	7.6660894958925	0.947	0.014	0		12	Upk3b
603	0	5.3985474764023	0.93	0.058	0		12	Epcam
604	0	7.5844187049593	0.864	0.007	0		12	Tm4sf5
605	0	6.92449903587497	0.87	0.015	0		12	Krt7
606	0	7.51501135579564	0.847	0.009	0		12	Lgals2
607	0	5.50196263366003	0.851	0.03	0		12	Lgr5
608	0	7.1315925054287	0.703	0.006	0		12	Upk1b
609	0	5.99974467383251	0.711	0.017	0		12	Tspan8
610	0	6.20120616135559	0.703	0.012	0		12	Unc45b

611	0	7.41012748388298	0.626	0.003	0	12	Muc16
612	0	6.49750792821655	0.582	0.006	0	12	Gpm6a
613	0	5.71007268265112	0.554	0.011	0	12	Cfap161
614	0	6.29993091506056	0.532	0.005	0	12	Pkhd1l1
615	0	6.75421780788963	0.515	0.006	0	12	Msln
616	0	5.99112201961066	0.494	0.008	0	12	Car9
617	0	6.17591664851736	0.44	0.008	0	12	Cldn15
618	0	6.31745741365017	0.411	0.004	0	12	Adgrg2
619	0	5.66922035333737	0.407	0.006	0	12	Stum
620	0	6.15025169418457	0.394	0.005	0	12	Bnc1
621	0	6.25707183813159	0.38	0.004	0	12	Crb2
622	0	5.77043848995114	0.374	0.008	0	12	Tspan1
623	0	6.76943677752041	0.367	0.003	0	12	Pcnx2
624	0	6.26911879551526	0.366	0.005	0	12	Dmkn
625	0	6.4409880496349	0.351	0.005	0	12	Prr15l
626	0	6.44510641033384	0.332	0.004	0	12	Hsd17b2
627	0	6.19995249514032	0.325	0.003	0	12	Il17re
628	0	5.39582550882043	0.303	0.005	0	12	Lypd6b
629	0	6.74903238734525	0.297	0.002	0	12	Lrrn4
630	0	5.41264625240168	0.265	0.01	0	12	Svs5
631	0	7.17606164657595	0.243	0.001	0	12	Srd5a2
632	0	5.77873893181405	0.223	0.003	0	12	Wnt2b
633	0	5.99584792658648	0.222	0.002	0	12	Kcnf1
634	0	5.87890426945998	0.216	0.003	0	12	Slc23a3
635	0	6.76695630588888	0.208	0.001	0	12	Rarres1
636	0	7.16349814257093	0.194	0.001	0	12	Ppp2r2c
637	0	6.41577071092068	0.194	0.003	0	12	Sectm1b
638	0	6.12703831209148	0.19	0.003	0	12	Plet1
639	0	5.96900749691756	0.182	0.002	0	12	Clic3
640	0	5.53106389023202	0.168	0.002	0	12	Fndc1
641	0	5.77683237672345	0.153	0.002	0	12	A730046J19Rik
642	0	6.39269757127187	0.151	0.001	0	12	Chst4
643	0	6.17552263853335	0.143	0.002	0	12	Prph
644	0	6.23475680326336	0.141	0.001	0	12	Lad1
645	0	6.40672024919819	0.131	0.001	0	12	Ckmt1
646	0	6.08480862221738	0.13	0.002	0	12	Mcf2
647	0	5.91050485547843	0.121	0.001	0	12	Fam163a
648	0	7.19374385771718	0.121	0.001	0	12	Lrp2
649	0	5.70251932780686	0.117	0.002	0	12	Klk1
650	0	5.40642577647891	0.108	0.002	0	12	Rdh9
651	0	7.27453570133553	0.762	0.03	0	13	Sycp3
652	0	7.32330587513541	0.611	0.009	0	13	Lhx8
653	0	7.67441622407136	0.609	0.011	0	13	Sycp1
654	0	7.35904933648359	0.54	0.008	0	13	Mov10l1
655	0	7.76888575003242	0.528	0.013	0	13	Gm15389
656	0	7.39955513557622	0.483	0.003	0	13	Gm47448
657	0	7.28475005865488	0.472	0.004	0	13	Stk31
658	0	7.36474834234867	0.469	0.004	0	13	Mageb4
659	0	7.99681809510166	0.462	0.004	0	13	Syce3
660	0	7.52782130754535	0.455	0.004	0	13	Rbakdn
661	0	8.01289941301815	0.446	0.003	0	13	Trap1a

662	0	7.50578516197316	0.436	0.005	0	13	Gm11985
663	0	7.38898145363383	0.413	0.002	0	13	Gm4779
664	0	7.53120403564305	0.411	0.003	0	13	Fmr1nb
665	0	8.50111507569884	0.364	0.001	0	13	Gm44873
666	0	7.97747835016283	0.319	0.002	0	13	AC141429.2
667	0	7.8122878479542	0.305	0.001	0	13	Gm364
668	0	8.32715999067164	0.301	0.002	0	13	Tsga8
669	0	8.12544863935138	0.287	0.001	0	13	Gm31579
670	0	7.96077435409156	0.286	0.001	0	13	Sult1c1
671	0	7.285329457	0.285	0.001	0	13	Hormad1
672	0	7.3140005930854	0.278	0.002	0	13	Tktl1
673	0	7.89784692090348	0.267	0.001	0	13	Tex101
674	0	8.13723234582516	0.265	0.001	0	13	Pet2
675	0	7.52129711040276	0.255	0.001	0	13	Ankrd31
676	0	8.2008985782428	0.237	0.001	0	13	Gm15298
677	0	7.2813910406804	0.203	0.001	0	13	Tex13b
678	0	7.72753797356987	0.202	0	0	13	Kcnk18
679	0	7.67359184336759	0.202	0.001	0	13	Zfp42
680	0	7.44445874040326	0.202	0.001	0	13	Gm33301
681	0	7.69402169177906	0.191	0.001	0	13	Gm40538
682	0	7.81987149730668	0.18	0	0	13	Gm6890
683	0	8.19525319552127	0.172	0.001	0	13	Gc
684	0	7.3679978600781	0.168	0	0	13	D030068K23Rik
685	0	7.49893127492012	0.167	0.001	0	13	Meiob
686	0	7.68940732955147	0.145	0	0	13	Trim52
687	0	7.86610122390649	0.141	0	0	13	Dmrtb1
688	0	7.89615508214833	0.14	0	0	13	Tmc2
689	0	7.36451763706783	0.137	0	0	13	Gm44113
690	0	7.34587016620616	0.13	0	0	13	Gm11851
691	0	7.50386515142521	0.122	0	0	13	Gm20750
692	0	8.33971699320139	0.114	0	0	13	Rhox2a
693	0	8.58690914699814	0.114	0	0	13	Pdcl2
694	0	7.70411749606713	0.111	0	0	13	Rhox13
695	0	7.95503599976257	0.11	0	0	13	Gm44601
696	0	8.09874544134771	0.109	0	0	13	Gm6880
697	0	7.30930812192435	0.108	0	0	13	Gm43348
698	0	8.44703026255408	0.106	0	0	13	Gm4593
699	0	7.61362305409121	0.102	0	0	13	Xlr3c
700	0	8.66487829027706	0.1	0	0	13	4933404K13Rik
701	0	8.46144318925982	0.845	0.023	0	14	C1qb
702	0	6.57129658515518	0.811	0.021	0	14	Ctss
703	0	8.94306216729842	0.805	0.016	0	14	C1qa
704	0	9.15790891570163	0.785	0.011	0	14	C1qc
705	0	7.39119486106645	0.698	0.009	0	14	Aif1
706	0	6.37743139963484	0.709	0.033	0	14	Cd68
707	0	8.62872799657509	0.681	0.012	0	14	Pf4
708	0	8.60903688885784	0.646	0.004	0	14	Ms4a7
709	0	7.97521464754497	0.63	0.008	0	14	Trem2
710	0	6.84882537795472	0.602	0.011	0	14	Csf1r
711	0	6.24673820338801	0.575	0.01	0	14	Fcgr3
712	0	6.59519939591094	0.561	0.008	0	14	Ly86

713	0	6.01711589932099	0.555	0.009	0	14	Cd300c2
714	0	6.03642626873034	0.549	0.013	0	14	Mpeg1
715	0	7.38025275301051	0.511	0.004	0	14	C3ar1
716	0	6.44290969902512	0.497	0.007	0	14	Ifi207
717	0	7.65295640847462	0.492	0.008	0	14	Mrc1
718	0	8.68097830464459	0.469	0.003	0	14	Fcrls
719	0	7.22328490417982	0.468	0.007	0	14	Cx3cr1
720	0	7.95054625855209	0.442	0.003	0	14	Adgre1
721	0	6.5780691123216	0.441	0.006	0	14	Ms4a6d
722	0	6.13709267698874	0.413	0.005	0	14	Cd84
723	0	6.6462921703537	0.401	0.004	0	14	Fcgr1
724	0	6.5480913756968	0.405	0.014	0	14	Ccl7
725	0	6.28585581849628	0.382	0.005	0	14	Clec4a2
726	0	6.0965295873355	0.382	0.006	0	14	Clec12a
727	0	8.94850127916238	0.366	0.003	0	14	Ccl12
728	0	6.8453892800041	0.357	0.003	0	14	Tbxas1
729	0	6.37309219706146	0.328	0.004	0	14	Msr1
730	0	6.31594948893048	0.314	0.021	0	14	Gdf15
731	0	6.09781930256656	0.282	0.004	0	14	Cd86
732	0	6.03717329406513	0.258	0.005	0	14	Clec4a1
733	0	6.48168857892909	0.23	0.002	0	14	Arl11
734	0	6.59983012326231	0.225	0.003	0	14	Clec4n
735	0	6.11136160622668	0.223	0.004	0	14	F13a1
736	0	6.63910788437929	0.209	0.002	0	14	Tnfrsf11a
737	0	6.78133205970238	0.212	0.005	0	14	Ltc4s
738	0	6.58997623845549	0.197	0.008	0	14	Gpnmb
739	0	6.06325966989126	0.195	0.011	0	14	Arg1
740	0	7.0185428918679	0.177	0.001	0	14	Aoah
741	0	6.75796342926563	0.177	0.003	0	14	Slamf9
742	0	6.27588556291929	0.153	0.001	0	14	Hk3
743	0	7.42484311792604	0.147	0.001	0	14	Gpr34
744	0	7.79787297862794	0.148	0.002	0	14	Cbr2
745	0	5.99076549727299	0.134	0.002	0	14	Cd200r1
746	0	6.12661793068015	0.127	0.001	0	14	Tlr7
747	0	6.87546514622285	0.116	0.001	0	14	Kcnk13
748	0	7.40970345288276	0.112	0.001	0	14	Folr2
749	0	6.00613181372476	0.102	0.001	0	14	Tlr1
750	0	8.14687885521185	0.1	0	0	14	P2ry12
751	0	9.01364979915814	0.607	0.008	0	15	Il1b
752	0	8.23337630738419	0.577	0.024	0	15	Wfdc17
753	0	9.26490961109202	0.426	0.005	0	15	Ifitm6
754	0	9.05554257224741	0.425	0.006	0	15	Hp
755	0	10.1017226238684	0.409	0.002	0	15	Mcemp1
756	0	9.05864834999007	0.401	0.005	0	15	Slpi
757	0	10.8504758924947	0.419	0.047	0	15	S100a9
758	0	10.8363680891565	0.419	0.055	0	15	S100a8
759	0	7.97690284983362	0.367	0.004	0	15	Pilra
760	0	11.8045933058088	0.341	0.003	0	15	Stfa2l1
761	0	8.5462236375292	0.332	0.003	0	15	Cd300lf
762	0	11.7750192281694	0.332	0.011	0	15	Stfa1
763	0	11.5768353856787	0.314	0.01	0	15	Gm5483

764	0	9.39126101273239	0.305	0.002	0	15	Clec4e
765	0	11.0998889708855	0.306	0.003	0	15	Wfdc21
766	0	11.0752701042615	0.312	0.009	0	15	Retnlg
767	0	10.3120897642423	0.302	0.001	0	15	Trem1
768	0	11.4547132139699	0.299	0.004	0	15	Stfa2
769	0	8.55856322399165	0.294	0.003	0	15	Csf3r
770	0	11.1271040421576	0.305	0.014	0	15	BC100530
771	0	8.07814575501719	0.295	0.008	0	15	Il1r2
772	0	10.8939674532854	0.278	0.004	0	15	2010005H15Rik
773	0	9.59818223312076	0.272	0.002	0	15	Hcar2
774	0	10.2174298336006	0.265	0.004	0	15	Asprv1
775	0	9.66297153562298	0.252	0.002	0	15	Mmp9
776	0	10.9274243557998	0.243	0.003	0	15	Stfa3
777	0	9.07411097243288	0.243	0.009	0	15	Lcn2
778	0	7.99871269640871	0.223	0.001	0	15	Pilrb2
779	0	10.4098384648159	0.217	0.001	0	15	Mmp8
780	0	12.4615891604329	0.216	0	0	15	Gm5416
781	0	9.94670197508767	0.215	0.001	0	15	Acod1
782	0	11.6069843435965	0.209	0	0	15	Trem3
783	0	8.76794344052369	0.198	0.002	0	15	Chil3
784	0	8.05052166471385	0.2	0.007	0	15	Chil1
785	0	9.08716978732473	0.193	0.001	0	15	Sirpb1c
786	0	9.72360646907846	0.185	0.001	0	15	F630028O10Rik
787	0	11.9676850608984	0.16	0	0	15	Il1f9
788	0	9.82445489290367	0.16	0.001	0	15	Fpr1
789	0	9.08842540399966	0.154	0.001	0	15	Fpr2
790	0	10.2921993920549	0.147	0	0	15	Mrgpra2b
791	0	9.57860488390928	0.144	0.001	0	15	Tarm1
792	0	9.43527929584235	0.136	0.001	0	15	Sirpb1b
793	0	8.40403260906437	0.135	0.002	0	15	Cxcr2
794	0	8.27237967856039	0.136	0.003	0	15	Prok2
795	0	7.98147854292511	0.123	0.001	0	15	1600010M07Rik
796	0	10.3383485394057	0.126	0.005	0	15	Ngp
797	0	9.65087442448697	0.119	0	0	15	Cd209a
798	0	8.06740636175593	0.116	0.002	0	15	Gm5150
799	0	9.24028482640739	0.114	0	0	15	Trim30b
800	0	7.9152763276789	0.115	0.001	0	15	Mefv
801	0	7.81725034872973	0.866	0.02	0	16	Ndufa4l2
802	0	6.35459757152122	0.872	0.041	0	16	Ebf1
803	0	8.54819092742414	0.792	0.01	0	16	Rgs5
804	0	4.77634903441957	0.859	0.115	0	16	Pdgfrb
805	0	4.95329703046044	0.817	0.112	0	16	Itga1
806	0	5.65816097796863	0.713	0.049	0	16	Abcc9
807	0	10.0652683388632	0.627	0.002	0	16	Higd1b
808	0	5.01101760194846	0.733	0.111	0	16	Gm13889
809	0	5.11117623337181	0.664	0.056	0	16	Cox4i2
810	0	6.39225676832322	0.623	0.022	0	16	Kcnj8
811	0	5.86134366374425	0.604	0.018	0	16	Aspn
812	0	5.69064437965702	0.593	0.027	0	16	Notch3
813	0	4.53578009468641	0.534	0.045	0	16	Des
814	0	6.2037752455424	0.477	0.015	0	16	Ace2

815	0	5.24116717705062	0.485	0.033	0	16	Gpc6
816	0	4.7671848156162	0.495	0.051	0	16	Cspg4
817	0	4.39702113595229	0.423	0.036	0	16	Mcam
818	0	4.43578713039125	0.396	0.032	0	16	S1pr3
819	0	4.87438871680798	0.387	0.031	0	16	Apold1
820	0	5.99395072908603	0.346	0.011	0	16	Prkg1
821	0	8.10306224623874	0.33	0.003	0	16	Cpxm2
822	0	4.50562883288774	0.348	0.047	0	16	Rgs16
823	0	4.88688743455522	0.323	0.022	0	16	Ednrb
824	0	5.55878301108583	0.311	0.012	0	16	Nostrin
825	0	4.74017594709973	0.311	0.023	0	16	Tbx3os1
826	0	4.71074872291918	0.307	0.025	0	16	Gja4
827	0	7.39561339306932	0.286	0.004	0	16	Agtr1a
828	0	4.68477814839616	0.291	0.019	0	16	Inpp4b
829	0	6.33776513982094	0.271	0.008	0	16	Rgs4
830	0	4.35149099010404	0.29	0.032	0	16	Adamts4
831	0	4.32750788425141	0.283	0.027	0	16	Npr3
832	0	5.9170185549776	0.264	0.012	0	16	Frem2
833	0	7.33220625293265	0.256	0.004	0	16	Ccl11
834	0	6.25295604865762	0.258	0.008	0	16	Itga7
835	0	4.83874601657517	0.217	0.011	0	16	Pcdh1
836	0	7.57800402021095	0.195	0.002	0	16	Vtn
837	0	9.07831916370049	0.192	0.001	0	16	Lgi1
838	0	5.71481626523629	0.189	0.009	0	16	Hrc
839	0	5.75075787392267	0.187	0.01	0	16	Mustn1
840	0	4.67983086681235	0.184	0.01	0	16	Adap2
841	0	7.01349458905927	0.174	0.002	0	16	Map3k7cl
842	0	5.39379792682023	0.177	0.009	0	16	Dgkb
843	0	5.07310481784829	0.17	0.008	0	16	Rasl12
844	0	6.36447593260314	0.13	0.002	0	16	Mc2r
845	0	6.47903123201353	0.122	0.002	0	16	Gm14005
846	0	6.48907183314561	0.121	0.002	0	16	Bmp5
847	4.11589787937539e-291	4.85944712602236	0.195	0.016	2.33700681590935e-286	16	Rcan2
848	4.78760068362558e-284	4.82826545474623	0.165	0.012	2.71839966816261e-279	16	Foxc1
849	8.59384962862952e-205	4.74198799818009	0.107	0.007	4.87958781913584e-200	16	Jph2
850	1.05141882550695e-201	4.26086450146446	0.203	0.025	5.96995609122847e-197	16	Nrarp
851	0	9.19137373485336	0.617	0.004	0	17	Ptprcap
852	0	8.63133311592166	0.562	0.011	0	17	AW112010
853	0	8.81385270859188	0.469	0.003	0	17	Cd69
854	0	9.57417558688456	0.467	0.004	0	17	Trbc2
855	0	9.9809552894244	0.452	0.007	0	17	Ccl5
856	0	11.3000082058477	0.436	0.001	0	17	Nkg7
857	0	10.4836533774457	0.402	0.001	0	17	Ms4a4b
858	0	10.9939541100178	0.4	0.001	0	17	Cd3g
859	0	8.30164677667243	0.371	0.006	0	17	Ctsw
860	0	10.484570529818	0.364	0.001	0	17	Gimap3
861	0	10.0637465244131	0.333	0.001	0	17	Cd3d
862	0	9.3887608625649	0.322	0.002	0	17	Il2rb
863	0	10.4707630013512	0.3	0.001	0	17	Trbc1
864	0	9.6809489028204	0.284	0.001	0	17	Cd2
865	0	11.0178732661547	0.255	0.001	0	17	Ifng

866	0	10.3114604364797	0.247	0.001	0	17	Xcl1
867	0	9.96596314234601	0.24	0.001	0	17	Sh2d2a
868	0	12.3373641411802	0.238	0	0	17	Gimap7
869	0	11.9384446347089	0.234	0	0	17	Cd3e
870	0	9.17298671993708	0.226	0.002	0	17	Cd28
871	0	11.4603799630953	0.217	0.001	0	17	Igkc
872	0	8.38003899191144	0.214	0.002	0	17	Itk
873	0	8.5724160126198	0.214	0.009	0	17	Cd79a
874	0	9.83352648562977	0.203	0.001	0	17	Cxcr6
875	0	8.24021273325328	0.197	0.001	0	17	Ccr7
876	0	9.1357423494776	0.193	0.002	0	17	Gm8369
877	0	11.0723023764902	0.191	0.001	0	17	Gzma
878	0	11.371540140986	0.188	0	0	17	Ikzf3
879	0	10.1769765713477	0.184	0	0	17	Gzmb
880	0	10.708660943423	0.171	0.001	0	17	Iglc2
881	0	9.37252604908392	0.169	0.001	0	17	Klre1
882	0	8.62648884246473	0.166	0.001	0	17	Cd226
883	0	9.09613019978059	0.162	0.002	0	17	Klrb1c
884	0	9.88967059449644	0.153	0.001	0	17	Prf1
885	0	9.10103892488412	0.152	0.001	0	17	Iglc3
886	0	10.7805943364548	0.145	0	0	17	Ncr1
887	0	10.0742158043491	0.145	0	0	17	Gm19585
888	0	9.31599228004401	0.141	0	0	17	Cxcr3
889	0	8.85266270978177	0.14	0.001	0	17	Tespa1
890	0	8.56338909480438	0.136	0.001	0	17	Icos
891	0	11.4410230297117	0.128	0	0	17	Ighd
892	0	11.3538541328977	0.122	0	0	17	Gm2682
893	0	8.74278084423018	0.124	0.002	0	17	Ms4a1
894	0	9.66024313945897	0.112	0.001	0	17	Fcmr
895	0	10.8773103796977	0.11	0	0	17	Sh2d1a
896	0	11.7737346713695	0.107	0	0	17	Klrc1
897	0	8.80497545536957	0.107	0.001	0	17	Cd8b1
898	0	8.24178573306967	0.102	0.001	0	17	Tbx21
899	0	8.98938598467596	0.1	0.001	0	17	Klrb1b
900	0	8.63771772180564	0.1	0.001	0	17	Cd5
901	0	2.75475347396903	0.553	0.032	0	18	Figla
902	0	3.37691933400583	0.529	0.02	0	18	Sycp1
903	8.98835476029671e-229	2.80926487115782	0.494	0.034	5.10358783289647e-224	18	Smc1b
904	2.06289455719873e-179	2.95891914091851	0.324	0.019	1.17131152957744e-174	18	Lhx8
905	1.49025573360001e-178	3.40788121265034	0.253	0.012	8.46167205538087e-174	18	Syce3
906	1.60087826095728e-155	2.98255329093233	0.282	0.016	9.08978676571542e-151	18	Mov10l1
907	3.14753985197697e-149	2.93266954059051	0.318	0.021	1.78717312795252e-144	18	Tdrd1
908	2.33920966771919e-133	2.88170697785058	0.247	0.015	1.32820324933095e-128	18	Syce1
909	1.65925758709671e-128	3.07863436245006	0.247	0.015	9.42126457953512e-124	18	Esrp1
910	2.1848985827735e-124	2.80718722567116	0.288	0.021	1.2405854152988e-119	18	Cpeb1
911	8.55891914743834e-119	3.26356107293574	0.512	0.067	4.85975429191549e-114	18	Crygb
912	1.86262837165413e-115	3.32729394524687	0.206	0.012	1.05760038942522e-110	18	Gm11985
913	1.40166429524565e-106	2.76736313211659	0.318	0.029	7.9586498684048e-102	18	Ybx2
914	4.30780204866744e-96	2.94025644212999	0.188	0.012	2.44597000323337e-91	18	Mageb4
915	1.56167953855218e-94	3.17449393539503	0.235	0.018	8.86721641989925e-90	18	Crlf1
916	5.0623664901597e-94	2.95324574914538	0.206	0.014	2.87441169311268e-89	18	Slc25a31

917	3.28412223706078e-91	3.30010448240768	0.171	0.01	1.86472460620311e-86	18	Trap1a
918	1.867664312202e-80	2.7772293975846	0.206	0.016	1.06045979646829e-75	18	Piwil2
919	7.74759614332339e-71	3.195449484	0.159	0.011	4.39908509017902e-66	18	Rbakdn
920	1.7478825293243e-67	3.04606603567475	0.159	0.012	9.9244770015034e-63	18	Tdrd9
921	1.49039119892077e-63	3.00162540050632	0.153	0.012	8.46244122747213e-59	18	Stk31
922	4.11844708077944e-63	3.24991409012257	0.294	0.04	2.33845425246657e-58	18	U90926
923	4.70592236862065e-63	3.43327873973405	0.165	0.013	2.6720227209028e-58	18	Ptprv
924	9.40247749370308e-62	3.13280711762685	0.241	0.028	5.33872672092461e-57	18	Atp10d
925	1.61525163887443e-59	2.77383681654462	0.253	0.031	9.17139880552903e-55	18	Rpl37rt
926	5.50522985961779e-56	2.81907138664414	0.288	0.042	3.12586951429098e-51	18	Acsm3
927	2.650920156818e-55	3.40798791647623	0.124	0.009	1.50519246504126e-50	18	Rpl10l
928	2.77076306843465e-51	2.8501087871842	0.118	0.009	1.5732392702572e-46	18	Taf7l
929	3.33153201383348e-49	2.89149486689924	0.118	0.009	1.89164387745465e-44	18	Tex12
930	2.26556134975315e-47	2.90517875286983	0.194	0.023	1.28638573438984e-42	18	AC122818.3
931	2.85647545660839e-45	2.86032700781376	0.112	0.009	1.62190676426224e-40	18	Phactr3
932	1.12245370986219e-43	2.93999162991565	0.153	0.016	6.3732921645975e-39	18	Crygc
933	1.43429739648789e-41	3.31369305935052	0.112	0.009	8.14394061725822e-37	18	mt-Td
934	4.5066043958414e-41	2.7461070806863	0.129	0.013	2.55884997595875e-36	18	AU015836
935	4.70129006144827e-41	3.21201845758771	0.141	0.015	2.66939249689033e-36	18	Gm47493
936	5.42327347227523e-38	3.1902138518758	0.194	0.029	3.07933467755788e-33	18	Cpa2
937	1.84700802717424e-37	2.99332551206738	0.1	0.008	1.04873115782953e-32	18	Gm47918
938	2.99286647657641e-37	2.7909208003782	0.112	0.01	1.69934958540009e-32	18	Pdzk1
939	2.13747304360128e-36	2.853038768	0.188	0.028	1.21365719415681e-31	18	Gm34220
940	4.62482544594013e-34	2.78162838544675	0.129	0.015	2.62597588820481e-29	18	Gm2694
941	1.85098920730095e-32	2.80193435396893	0.1	0.01	1.05099167190548e-27	18	Fmr1nb
942	3.11194625885147e-29	2.8566178846004	0.118	0.014	1.76696308577586e-24	18	Ggnbp1.1
943	3.33756332009209e-29	2.76598931294561	0.141	0.02	1.89506845314829e-24	18	Gm37423
944	6.33508614722973e-29	2.82345223059927	0.135	0.019	3.59706191439704e-24	18	4930431P19Rik
945	1.74174226301826e-27	3.28200314440023	0.124	0.017	9.88961256941769e-23	18	Gata1
946	4.62849837225961e-26	3.10891892888773	0.112	0.014	2.62806137576901e-21	18	Pcsk1n
947	1.36958117574096e-25	2.89142074699701	0.118	0.016	7.77648191585717e-21	18	3100003L05Rik
948	8.20315935870467e-25	2.86370045218859	0.124	0.018	4.65775388387251e-20	18	Gm17660
949	2.58314232995132e-22	2.91570374807853	0.1	0.013	1.46670821494636e-17	18	A130012E19Rik
950	3.4645058463578e-19	3.10814202915343	0.1	0.015	1.96714641956196e-14	18	Cst9

Supplementary Table S2

	p_val	avg_log2FC	pct.1	pct.2	p_val_adj	cluster	gene
Meg3	0	1.94864745686217	0.819	0.423	0	0	Meg3
Igfbp5	0	1.71116892885139	0.737	0.392	0	0	Igfbp5
Lsp1	0	1.67936556828736	0.824	0.472	0	0	Lsp1
mt-Rnr1	0	1.57178047609966	0.496	0.128	0	0	mt-Rnr1
Ptn	0	1.56620463586592	0.836	0.453	0	0	Ptn
mt-Rnr2	0	1.5516410783743	0.5	0.129	0	0	mt-Rnr2
H19	0	1.50569756609379	0.602	0.196	0	0	H19
Dlk1	0	1.20657683421972	0.707	0.294	0	0	Dlk1
Gucy1a1	0	1.1440125040537	0.68	0.491	0	0	Gucy1a1
Eln	0	1.13354212895415	0.535	0.289	0	0	Eln
Atp5o.1	0	1.08422712079616	0.57	0.17	0	0	Atp5o.1
Sfrp1	0	1.05401383674491	0.697	0.526	0	0	Sfrp1
Col1a1	0	1.03447859095799	0.935	0.844	0	0	Col1a1
Mfap2	0	0.996096938676772	0.89	0.72	0	0	Mfap2
Tgfbi	0	0.991441140913357	0.63	0.409	0	0	Tgfbi
Gas1	0	0.989465435180055	0.639	0.359	0	0	Gas1
Nrep	0	0.978509505074824	0.613	0.358	0	0	Nrep
Igfbp4	0	0.933500281053842	0.865	0.715	0	0	Igfbp4
Nfib	0	0.920274103903003	0.726	0.57	0	0	Nfib
Peg3	0	0.915341170035941	0.624	0.378	0	0	Peg3
Loxl1	0	0.908902234293577	0.629	0.385	0	0	Loxl1
Col3a1	0	0.906666135993988	0.954	0.886	0	0	Col3a1
Cdkn1c	0	0.903836305086104	0.648	0.361	0	0	Cdkn1c
Mfap4	0	0.899503440455093	0.853	0.63	0	0	Mfap4
Usmg5	0	0.852402288953584	0.507	0.164	0	0	Usmg5
Ncam1	0	0.836909334043793	0.543	0.289	0	0	Ncam1
Mdk	0	0.836067694165263	0.911	0.852	0	0	Mdk
Adamts2	0	0.830630571360365	0.628	0.44	0	0	Adamts2
Minos1	0	0.823571135008063	0.479	0.159	0	0	Minos1
2410015M20Rik	0	0.803420988898325	0.464	0.148	0	0	2410015M20Rik
Islr	0	0.780923748385172	0.687	0.521	0	0	Islr
Rbp1	0	0.757982454310328	0.897	0.882	0	0	Rbp1
Auts2	0	0.747589263661536	0.783	0.684	0	0	Auts2
Mmp2	0	0.745274378073869	0.756	0.623	0	0	Mmp2
Ccnd2	0	0.728296570542313	0.791	0.68	0	0	Ccnd2
Aes	0	0.697730025083667	0.437	0.136	0	0	Aes
Col9a2	0	0.69295412602098	0.356	0.139	0	0	Col9a2
Lbhd2	0	0.673061545849426	0.312	0.102	0	0	Lbhd2
Rrbp1	0	0.672211817937193	0.891	0.896	0	0	Rrbp1
6820431F20Rik	0	0.655649864077304	0.264	0.072	0	0	6820431F20Rik
Fstl1	0	0.650541296881201	0.812	0.727	0	0	Fstl1
Col1a2	0	0.570356689868984	0.962	0.931	0	0	Col1a2
Rps27	0	0.435996550263957	0.994	0.998	0	0	Rps27
Rps29	0	0.363927882151237	0.998	0.999	0	0	Rps29
Slit2	2.00290457891133e-307	0.713811782781255	0.355	0.136	1.13724921990585e-302	0	Slit2
Col6a2	4.33846693379265e-302	0.803959814015292	0.796	0.72	2.46338152500746e-297	0	Col6a2
Atxn7l3b	3.79867833814425e-299	0.600836115133865	0.816	0.768	2.15688956039831e-294	0	Atxn7l3b
Nenf	4.81494732742552e-292	0.507596657575002	0.877	0.872	2.73392709251221e-287	0	Nenf
1700020I14Rik	4.56072864788372e-288	0.586045402436067	0.29	0.095	2.58958172626838e-283	0	1700020I14Rik

Sparc	3.60111462040168e-286	0.410051836374017	0.965	0.946	2.04471288146407e-281	0	Sparc
Bst2	0	1.31883707045136	0.843	0.371	0	1	Bst2
Ddit4l	0	1.24266079770321	0.657	0.249	0	1	Ddit4l
Tmem100	0	1.22528791704343	0.617	0.171	0	1	Tmem100
Nme2	0	1.19889327122553	0.946	0.513	0	1	Nme2
Plac8	0	1.17454897275317	0.573	0.179	0	1	Plac8
Akr1cl	0	1.09275002989131	0.965	0.729	0	1	Akr1cl
Ptch1	0	1.05346622221841	0.645	0.287	0	1	Ptch1
Igfbp7	0	0.946114767441671	0.993	0.917	0	1	Igfbp7
Rpl13a	0	0.935761491467211	0.997	0.885	0	1	Rpl13a
Rps10	0	0.92111764640278	0.999	0.957	0	1	Rps10
Cox6c	0	0.917969692155423	0.995	0.913	0	1	Cox6c
Rpl15	0	0.910528446934161	0.999	0.955	0	1	Rpl15
Tle5	0	0.902569311890064	0.856	0.365	0	1	Tle5
Rpl27	0	0.899464833615174	0.996	0.933	0	1	Rpl27
Smim41	0	0.877936780925937	0.532	0.136	0	1	Smim41
Rpl36al	0	0.81245607793963	0.969	0.696	0	1	Rpl36al
Rpl29	0	0.803187275277695	0.997	0.967	0	1	Rpl29
Rpl18	0	0.802445469888649	0.999	0.989	0	1	Rpl18
Rpl24	0	0.80134923646592	0.999	0.949	0	1	Rpl24
2410006H16Rik	0	0.790971613106676	0.926	0.718	0	1	2410006H16Rik
Atp5g2	0	0.782335157883211	0.952	0.557	0	1	Atp5g2
Rpl10a	0	0.77944625745796	0.999	0.986	0	1	Rpl10a
Rpl27a	0	0.776243952297382	1	0.988	0	1	Rpl27a
Atp5o	0	0.77488316033382	0.829	0.339	0	1	Atp5o
Atp5md	0	0.755516844070324	0.821	0.346	0	1	Atp5md
Fhl2	0	0.735770124028953	0.716	0.389	0	1	Fhl2
Tpm1	0	0.730356126191153	0.988	0.916	0	1	Tpm1
Smoc2	0	0.714392567733306	0.499	0.188	0	1	Smoc2
Rps2	0	0.697774615446884	1	0.992	0	1	Rps2
Rps7	0	0.695328034325702	1	0.992	0	1	Rps7
Micos13	0	0.689090406626996	0.738	0.284	0	1	Micos13
Enpep	0	0.682359012952349	0.409	0.123	0	1	Enpep
Rpl30	0	0.659551890491631	1	0.994	0	1	Rpl30
Micos10	0	0.65952912429678	0.818	0.352	0	1	Micos10
Gstm1	0	0.650706621860319	0.886	0.589	0	1	Gstm1
Rpsa	0	0.646865212308204	1	0.994	0	1	Rpsa
Rbis	0	0.645901676880984	0.687	0.267	0	1	Rbis
Grcc10	0	0.640729931315189	0.821	0.445	0	1	Grcc10
Lamc3	0	0.640407854645019	0.395	0.086	0	1	Lamc3
Ppp1r14b	0	0.622026576909446	0.812	0.503	0	1	Ppp1r14b
Actr3b	0	0.60931486576315	0.615	0.276	0	1	Actr3b
Rpl7	0	0.587743878154324	0.999	0.981	0	1	Rpl7
Rplp0	0	0.587708655729325	1	0.994	0	1	Rplp0
Tomm6	0	0.586802628073272	0.713	0.329	0	1	Tomm6
Rpl13	0	0.577983857674398	1	0.999	0	1	Rpl13
Rpl11	0	0.566107737576528	1	0.994	0	1	Rpl11
Rnasek	0	0.55577257521886	0.644	0.268	0	1	Rnasek
Rpl7a	0	0.55147486971429	0.994	0.967	0	1	Rpl7a
Rpl37	0	0.546055379271661	0.999	0.996	0	1	Rpl37
Timm23	0	0.543902257799602	0.608	0.244	0	1	Timm23

Hspa1a	0	2.62747425687237	0.822	0.343	0	2	Hspa1a
Hspa1b	0	2.51805780286686	0.757	0.274	0	2	Hspa1b
Hsph1	0	2.25992388495393	0.669	0.257	0	2	Hsph1
Ppp1r15a	0	2.09081193656241	0.923	0.553	0	2	Ppp1r15a
Dnajb1	0	2.0230698537125	0.848	0.463	0	2	Dnajb1
Hsp90aa1	0	1.98375234844727	0.974	0.902	0	2	Hsp90aa1
Ier3	0	1.98362119381153	0.812	0.401	0	2	Ier3
Cebpb	0	1.84594652964437	0.819	0.454	0	2	Cebpb
Junb	0	1.83389202551824	0.975	0.723	0	2	Junb
Hspb1	0	1.79344683525995	0.551	0.229	0	2	Hspb1
Mt1l	0	1.78657845010371	0.842	0.448	0	2	Mt1
Fos	0	1.77075767319473	0.982	0.793	0	2	Fos
Fosb	0	1.7659858854754	0.908	0.564	0	2	Fosb
Gm12840	0	1.75097138941693	0.527	0.161	0	2	Gm12840
Mt2	0	1.72909993753505	0.641	0.235	0	2	Mt2
Adamts1	0	1.70814863250796	0.588	0.319	0	2	Adamts1
Klf4	0	1.70131626840201	0.724	0.373	0	2	Klf4
Gm42418	0	1.66095316940978	0.999	0.984	0	2	Gm42418
Pim1	0	1.63521554548074	0.721	0.37	0	2	Pim1
Atf3	0	1.6350262890865	0.818	0.493	0	2	Atf3
Nop58	0	1.6323834812188	0.805	0.6	0	2	Nop58
Cxcl1	0	1.62377369299846	0.466	0.184	0	2	Cxcl1
Egr1	0	1.6188791483988	0.976	0.779	0	2	Egr1
Ppp1r10	0	1.59515847618944	0.661	0.382	0	2	Ppp1r10
Sqstm1	0	1.57061473029665	0.828	0.59	0	2	Sqstm1
Ubc	0	1.5641619378473	0.981	0.915	0	2	Ubc
Gadd45b	0	1.55634781733437	0.834	0.548	0	2	Gadd45b
Apoe	0	1.53510593975834	0.828	0.631	0	2	Apoe
Cyp11a1	0	1.51641579750344	0.674	0.197	0	2	Cyp11a1
Dnajb9	0	1.49747851996324	0.648	0.335	0	2	Dnajb9
Cxcl10	0	1.48528539140449	0.556	0.275	0	2	Cxcl10
Gem	0	1.46472742094591	0.558	0.269	0	2	Gem
Bag3	0	1.44392652610269	0.575	0.251	0	2	Bag3
Cdkn1a	0	1.44091755739358	0.467	0.15	0	2	Cdkn1a
Neat1	0	1.43905285353405	0.507	0.173	0	2	Neat1
Nfkbia	0	1.41970307313128	0.852	0.622	0	2	Nfkbia
Ddx3x	0	1.3849199075568	0.787	0.597	0	2	Ddx3x
Pnrc1	0	1.36762545326674	0.925	0.774	0	2	Pnrc1
Klf9	0	1.3579426022088	0.711	0.406	0	2	Klf9
Lars2	0	1.35060523361728	0.862	0.498	0	2	Lars2
Nr4a2	0	1.34518030575664	0.39	0.123	0	2	Nr4a2
Slc38a2	0	1.30406060530632	0.881	0.721	0	2	Slc38a2
Errfi1	0	1.28816489398278	0.514	0.216	0	2	Errfi1
Ifrd1	0	1.28539822276768	0.79	0.571	0	2	Ifrd1
Clk1	0	1.27415878817026	0.873	0.707	0	2	Clk1
Tiparp	0	1.27324167732637	0.704	0.474	0	2	Tiparp
AY036118	0	1.25757028959294	0.823	0.446	0	2	AY036118
Ccnl1	0	1.24668442724755	0.859	0.653	0	2	Ccnl1
Rbbp6	0	1.24335690012876	0.759	0.607	0	2	Rbbp6
Bhlhe40	0	1.21598428326963	0.452	0.201	0	2	Bhlhe40
Hmgb2	0	2.67191337035917	0.977	0.656	0	3	Hmgb2

Cenpf	0	2.47521900346218	0.859	0.077	0	3	Cenpf
Mki67	0	2.33323134393527	0.819	0.078	0	3	Mki67
Cenpa	0	2.28287095670045	0.858	0.069	0	3	Cenpa
Stmn1	0	2.17843923445805	0.987	0.588	0	3	Stmn1
Ube2c	0	2.09031576197596	0.661	0.086	0	3	Ube2c
Birc5	0	2.08535964288646	0.847	0.083	0	3	Birc5
Top2a	0	2.08157547141498	0.612	0.122	0	3	Top2a
Cks2	0	1.95240256538929	0.844	0.292	0	3	Cks2
Cenpe	0	1.88412832572233	0.748	0.057	0	3	Cenpe
Prc1	0	1.86330929985386	0.57	0.082	0	3	Prc1
Ccnb2	0	1.81627767536672	0.833	0.042	0	3	Ccnb2
Tpx2	0	1.79632639359311	0.726	0.059	0	3	Tpx2
H2afz	0	1.73990092225246	0.997	0.909	0	3	H2afz
Cdca8	0	1.70321096001073	0.74	0.058	0	3	Cdca8
Tubb4b	0	1.69008841152159	0.896	0.444	0	3	Tubb4b
Nucks1	0	1.65784456431208	0.963	0.585	0	3	Nucks1
Cdca3	0	1.6041389310929	0.762	0.04	0	3	Cdca3
Smc4	0	1.55653185247004	0.802	0.259	0	3	Smc4
Cdc20	0	1.5468839817871	0.629	0.049	0	3	Cdc20
H2afx	0	1.5336108389288	0.728	0.239	0	3	H2afx
Selenoh	0	1.50431929730355	0.937	0.454	0	3	Selenoh
Hmmr	0	1.44255145173063	0.587	0.02	0	3	Hmmr
Lockd	0	1.39997592887466	0.751	0.061	0	3	Lockd
H2afv	0	1.38553130166279	0.981	0.742	0	3	H2afv
Hmgb1	0	1.37328544753511	0.999	0.955	0	3	Hmgb1
Pclaf	0	1.35980649282607	0.694	0.108	0	3	Pclaf
Lmnb1	0	1.31944773156745	0.794	0.241	0	3	Lmnb1
Nusap1	0	1.31279108610255	0.526	0.046	0	3	Nusap1
Tmpo	0	1.26746910663838	0.865	0.419	0	3	Tmpo
Racgap1	0	1.24106628050166	0.659	0.039	0	3	Racgap1
Spc24	0	1.22709396308235	0.697	0.084	0	3	Spc24
Knstrn	0	1.22653294218135	0.665	0.042	0	3	Knstrn
Ran1	0	1.21947930363183	0.968	0.706	0	3	Ran
Ckap2l	0	1.20058382004598	0.58	0.033	0	3	Ckap2l
Kif23	0	1.19162004680868	0.602	0.05	0	3	Kif23
Ccna2	0	1.1848878520415	0.588	0.038	0	3	Ccna2
Ccnb1	0	1.17583014156663	0.562	0.025	0	3	Ccnb1
Smc2	0	1.17263878697103	0.674	0.201	0	3	Smc2
Knl1	0	1.14176195193196	0.569	0.039	0	3	Knl1
Ccdc34	0	1.11528114180068	0.872	0.387	0	3	Ccdc34
Cdkn3	0	1.06658539253625	0.583	0.021	0	3	Cdkn3
Tacc3	0	1.05534832727651	0.629	0.051	0	3	Tacc3
Aspm	0	1.03750469045826	0.476	0.047	0	3	Aspm
Ckap2	0	1.02634025712857	0.552	0.045	0	3	Ckap2
Cenpm	0	1.02242071159354	0.686	0.102	0	3	Cenpm
Pimreg	0	1.01820382357751	0.487	0.016	0	3	Pimreg
Spc25	0	0.975937533118235	0.489	0.047	0	3	Spc25
Kif11	0	0.966780110730771	0.476	0.027	0	3	Kif11
Anln	0	0.961311293602422	0.536	0.041	0	3	Anln
Pclaf1	0	2.43427278865835	0.857	0.1	0	4	Pclaf
Lig1	0	1.73777369002797	0.943	0.219	0	4	Lig1

Hells	0	1.53544572113194	0.853	0.144	0	4	Hells
Stmn11	0	1.40093049342022	0.977	0.59	0	4	Stmn1
Mcm3	0	1.34740535363794	0.817	0.134	0	4	Mcm3
Smc21	0	1.25085343740011	0.827	0.193	0	4	Smc2
Top2a1	0	1.25030252400702	0.645	0.121	0	4	Top2a
Mcm6	0	1.22953664272883	0.85	0.255	0	4	Mcm6
Dek1	0	1.22215128150567	0.991	0.697	0	4	Dek
Tyms1	0	1.21804088711122	0.739	0.149	0	4	Tyms
Pcna	0	1.20173882499728	0.89	0.329	0	4	Pcna
Ranbp11	0	1.13467436012201	0.962	0.627	0	4	Ranbp1
Dut1	0	1.11481890918223	0.841	0.315	0	4	Dut
Slfn9	0	1.11430480129344	0.54	0.038	0	4	Slfn9
Mcm7	0	1.08089854845147	0.796	0.221	0	4	Mcm7
Clspn1	0	1.06228612555864	0.615	0.062	0	4	Clspn
Fam111a	0	1.05889513912292	0.613	0.065	0	4	Fam111a
Gmnn1	0	1.04919267714549	0.663	0.091	0	4	Gmnn
Dnajc91	0	1.04557605127721	0.784	0.219	0	4	Dnajc9
Mcm5	0	1.03242413602561	0.688	0.119	0	4	Mcm5
Uhrf1	0	1.02493578029322	0.587	0.061	0	4	Uhrf1
Tk11	0	0.990932614953475	0.544	0.044	0	4	Tk1
Gins2	0	0.974736980967062	0.681	0.11	0	4	Gins2
Cenpm1	0	0.966561890061022	0.645	0.107	0	4	Cenpm
Syce2	0	0.921983542843794	0.641	0.13	0	4	Syce2
Cenph1	0	0.911303373358975	0.507	0.048	0	4	Cenph
Dtl	0	0.889265346276847	0.623	0.138	0	4	Dtl
Cenpk1	0	0.888858439008248	0.574	0.074	0	4	Cenpk
Mcm4	0	0.885621589991405	0.709	0.207	0	4	Mcm4
Dhfr	0	0.863859222557125	0.63	0.118	0	4	Dhfr
Dnmt1	0	0.862180820044663	0.675	0.178	0	4	Dnmt1
Rad51ap11	0	0.855518866335987	0.514	0.038	0	4	Rad51ap1
Rpa2	0	0.846027264858238	0.637	0.154	0	4	Rpa2
Rrm2	0	0.837788154368449	0.499	0.053	0	4	Rrm2
Rfc41	0	0.831861375881914	0.662	0.166	0	4	Rfc4
Rrm11	0	0.823628450962456	0.637	0.166	0	4	Rrm1
Rfc2	0	0.802979805222563	0.686	0.2	0	4	Rfc2
Mcm2	0	0.782791865458783	0.574	0.095	0	4	Mcm2
Spc241	0	0.780779056279938	0.517	0.097	0	4	Spc24
Tcf19	0	0.763005759767253	0.464	0.044	0	4	Tcf19
Chaf1a	0	0.756021854659081	0.536	0.085	0	4	Chaf1a
Ncapg21	0	0.698385963307907	0.445	0.07	0	4	Ncapg2
Ung	0	0.695299536327893	0.467	0.068	0	4	Ung
Chaf1b	0	0.692597222090365	0.454	0.038	0	4	Chaf1b
Prim1	0	0.686232967954217	0.555	0.125	0	4	Prim1
Diaph31	0	0.682133154297089	0.446	0.071	0	4	Diaph3
Rad51	0	0.672640966934945	0.435	0.037	0	4	Rad51
E2f1	0	0.639042813952927	0.454	0.056	0	4	E2f1
Dscc1	0	0.598133738541049	0.392	0.035	0	4	Dscc1
Atad5	0	0.578933954241386	0.418	0.073	0	4	Atad5
Tnfaip2	1.8496698344058e-301	0.71069970878624	0.275	0.028	1.05024253197561e-296	5	Tnfaip2
Ebf1	8.916838620535e-240	0.947822324356819	0.334	0.055	5.06298096873977e-235	5	Ebf1
Col14a1	6.37066664770689e-237	1.00606396260304	0.318	0.051	3.61726452256797e-232	5	Col14a1

S100a6	1.69049858214621e-228	1.7667600219945	0.869	0.481	9.5986509494262e-224	5	S100a6
Tm4sf11	2.54081471634418e-200	0.763502644587007	0.382	0.083	1.44267459594022e-195	5	Tm4sf1
Col15a11	2.1140371584633e-178	1.18341281597784	0.581	0.202	1.20035029857546e-173	5	Col15a1
Tmsb4x	2.82041325112101e-169	1.00483873338204	0.998	0.962	1.60143064398651e-164	5	Tmsb4x
Piezo2	5.29356344974172e-155	0.772297305550309	0.329	0.077	3.00568532676335e-150	5	Piezo2
Crip11	1.84067421533375e-152	0.962449629902794	0.484	0.161	1.04513481946651e-147	5	Crip1
Nnmt	1.15203413054495e-151	0.404654951044553	0.255	0.048	6.5412497932342e-147	5	Nnmt
Mrap	6.2932691380583e-130	0.629070677218475	0.316	0.081	3.5733182165895e-125	5	Mrap
Fxyd5	1.64122794174551e-124	0.500770638996803	0.318	0.083	9.31889225323099e-120	5	Fxyd5
Dpt	3.39548421531078e-112	1.13819930702446	0.283	0.077	1.92795593745346e-107	5	Dpt
Nupr11	2.96451330369914e-104	1.11923448325637	0.608	0.292	1.68325065384037e-99	5	Nupr1
Sparc1	4.35144181051546e-101	0.881317889912465	0.995	0.951	2.47074866001068e-96	5	Sparc
Ccl2	2.13599654347908e-98	1.11035604755263	0.287	0.082	1.21281883738742e-93	5	Ccl2
Timp1	3.28677840574684e-97	2.08245237087123	0.531	0.271	1.86623277878306e-92	5	Timp1
Ifitm32	1.68388501247255e-92	0.815089566655708	0.918	0.745	9.56109910081913e-88	5	Ifitm3
Plat1	3.0903200516122e-92	0.661495423859179	0.501	0.219	1.75468372530541e-87	5	Plat
Neat11	9.87144123879736e-89	1.11222669809836	0.486	0.224	5.60500433538914e-84	5	Neat1
Postn	1.19888039571508e-88	1.13564956779585	0.389	0.157	6.8072428868702e-84	5	Postn
H2-T23	4.19751554403734e-88	0.344744143640768	0.255	0.071	2.3833493259044e-83	5	H2-T23
Ecm11	2.13582749020739e-86	0.53049579533319	0.502	0.215	1.21272284893975e-81	5	Ecm1
Ahnak2	2.92931910149501e-85	0.320533214678317	0.254	0.071	1.66326738582887e-80	5	Ahnak2
Cxcl141	3.72633211067618e-85	1.51046855734354	0.413	0.173	2.11581137244194e-80	5	Cxcl14
Ctsc	3.79183400076263e-80	0.673634750237682	0.372	0.144	2.15300334563302e-75	5	Ctsc
Star1	2.77416566923289e-79	0.541488558338438	0.329	0.115	1.57517126699043e-74	5	Star
Anxa11	8.96364890501064e-79	0.615625295564893	0.437	0.191	5.08955984826504e-74	5	Anxa1
Cd63	2.8396897403273e-76	0.561594385079913	0.981	0.931	1.61237583455784e-71	5	Cd63
Fn11	1.10479556824876e-74	1.05196123201709	0.611	0.367	6.27302923651648e-70	5	Fn1
Nrp11	6.09464541271311e-72	0.529908785059173	0.498	0.237	3.4605396653385e-67	5	Nrp1
Sbno2	2.39039364444579e-71	0.358251061966507	0.296	0.102	1.35726551131632e-66	5	Sbno2
C1s11	1.01558614712164e-70	0.424607180345693	0.401	0.164	5.76649814335667e-66	5	C1s1
Lgals12	5.19875650609996e-70	0.754494639801299	0.987	0.898	2.95185394416356e-65	5	Lgals1
S100a11	6.60205421618401e-70	0.620794405902412	0.936	0.824	3.74864638394928e-65	5	S100a11
Lama4	7.27779719097355e-70	0.412715652910397	0.338	0.13	4.13233324503478e-65	5	Lama4
Ccdc80	5.63738809741249e-69	0.913473062107413	0.703	0.452	3.20090896171081e-64	5	Ccdc80
Pdgfrb1	1.77482329780227e-68	0.591763182093932	0.543	0.281	1.00774466849213e-63	5	Pdgfrb
Aebp1	3.60892873358436e-68	0.603970010873737	0.847	0.654	2.0491497349292e-63	5	Aebp1
Prlr	5.53558674766925e-68	0.786912045912904	0.357	0.15	3.1431061553266e-63	5	Prlr
Cst3	2.27790575626227e-67	0.687811108729203	0.972	0.85	1.29339488840572e-62	5	Cst3
Crip2	5.97186273862221e-67	0.351509882074082	0.283	0.099	3.39082366298969e-62	5	Crip2
Vat1	1.11618083175402e-66	0.332034179238468	0.331	0.127	6.33767476269935e-62	5	Vat1
Col4a22	1.34823463485085e-66	1.04532237902007	0.793	0.624	7.65527625668311e-62	5	Col4a2
Capg	2.93232208470583e-65	0.495842165973029	0.409	0.187	1.66497247969597e-60	5	Capg
Pcdh7	8.92940777341202e-65	0.378309386984731	0.409	0.177	5.07011773374334e-60	5	Pcdh7
S100a13	1.15236758714898e-64	0.413944461464979	0.507	0.249	6.54314315983191e-60	5	S100a13
Mndal	3.85689399483777e-63	0.268860028680964	0.332	0.126	2.18994441026889e-58	5	Mndal
Cald1	1.81930098373309e-62	0.632584030829591	0.968	0.891	1.03299909856365e-57	5	Cald1
Des	2.80081819632779e-62	0.376635945285153	0.274	0.099	1.59030457187492e-57	5	Des
Kctd14	0	2.90100512615233	0.958	0.238	0	6	Kctd14
Gstm21	0	2.60144774329116	0.976	0.516	0	6	Gstm2
Gstm6	0	2.42778062445244	0.846	0.029	0	6	Gstm6
mt-Rnr22	0	2.35081495186757	0.867	0.243	0	6	mt-Rnr2

Itga6	0	2.20905292453574	0.915	0.125	0	6	Itga6
Col18a1	0	2.18083801951655	0.894	0.074	0	6	Col18a1
Aes2	0	1.95384528133318	0.925	0.222	0	6	Aes
Chchd10	0	1.94260746222579	0.976	0.467	0	6	Chchd10
Kitl1	0	1.92572969612782	0.891	0.279	0	6	Kitl
Gatm1	0	1.86177347447853	0.858	0.184	0	6	Gatm
Sdc42	0	1.86092081800088	0.948	0.365	0	6	Sdc4
Pcsk6	0	1.77307600975688	0.906	0.163	0	6	Pcsk6
Wnt6	0	1.65133566194789	0.796	0.07	0	6	Wnt6
Lgals71	0	1.60214246187989	0.854	0.204	0	6	Lgals7
Gm35533	0	1.56270880624084	0.66	0.048	0	6	Gm35533
Cdkn1b1	0	1.55785572020928	0.902	0.314	0	6	Cdkn1b
Amhr2	0	1.54214355194422	0.826	0.079	0	6	Amhr2
Fst	0	1.50820849426165	0.711	0.094	0	6	Fst
Aard	0	1.48781975349199	0.717	0.048	0	6	Aard
Map1b	0	1.45783239117124	0.845	0.228	0	6	Map1b
Wfdc10	0	1.39712264617927	0.44	0.057	0	6	Wfdc10
Fndc5	0	1.27021694960773	0.787	0.152	0	6	Fndc5
2410015M20Rik1	0	1.24322090301415	0.906	0.241	0	6	2410015M20Rik
Cst12	0	1.16640110364145	0.358	0.033	0	6	Cst12
Smad3	0	1.14929906607833	0.744	0.116	0	6	Smad3
Hpcal1	0	1.0505391676493	0.765	0.161	0	6	Hpcal1
1190002N15Rik	0	0.995986237662304	0.654	0.096	0	6	1190002N15Rik
Gm45889	0	0.98295933284991	0.551	0.008	0	6	Gm45889
Emx2	0	0.982428316468157	0.705	0.1	0	6	Emx2
Me2	0	0.981651144330301	0.745	0.164	0	6	Me2
Spint2	0	0.966911145971337	0.659	0.043	0	6	Spint2
Arap2	0	0.948437421667989	0.545	0.032	0	6	Arap2
Irx3	0	0.933663489013933	0.516	0.021	0	6	Irx3
Fbxo21	0	0.902848564965377	0.692	0.145	0	6	Fbxo21
Aldh1b1	0	0.894087832697953	0.472	0.019	0	6	Aldh1b1
Dpf3	0	0.875975624031882	0.566	0.053	0	6	Dpf3
Ramp1	0	0.852365622957201	0.414	0.05	0	6	Ramp1
Ezr	0	0.823653218950942	0.638	0.111	0	6	Ezr
Sytl4	0	0.814530552863609	0.563	0.085	0	6	Sytl4
Etnk2	0	0.802512146752008	0.602	0.072	0	6	Etnk2
Gsta4	0	0.78005303824925	0.592	0.095	0	6	Gsta4
Slc26a7	0	0.761040415727663	0.507	0.073	0	6	Slc26a7
Gm14226	0	0.750532880435169	0.455	0.033	0	6	Gm14226
Cyfip2	0	0.733531143616135	0.48	0.021	0	6	Cyfip2
Krt79	0	0.711348869985363	0.373	0.016	0	6	Krt79
Aldh1a2	0	0.707860685827589	0.425	0.046	0	6	Aldh1a2
Espn	0	0.706830944196934	0.438	0.033	0	6	Espn
Crygb	0	0.684823387372064	0.273	0.007	0	6	Crygb
Cgn	0	0.684174127333755	0.435	0.011	0	6	Cgn
Zg16	0	0.67787305855779	0.401	0.008	0	6	Zg16
Mfap51	0	2.86338846453983	0.805	0.172	0	7	Mfap5
Myh11	0	2.63012361938585	0.763	0.113	0	7	Myh11
Actg2	0	1.89172154768559	0.439	0.038	0	7	Actg2
Lmod1	0	1.6295494666819	0.555	0.04	0	7	Lmod1
Cnn1	0	1.35307307874717	0.376	0.023	0	7	Cnn1

Fbxl22	1.7146800745871e-278	1.96275654427338	0.701	0.141	9.73595346350554e-274	7	Fbxl22
Tagln1	2.83028950659152e-260	2.78911085	0.988	0.444	1.60703838184267e-255	7	Tagln
Acta22	6.80038161946435e-250	3.0478099150017	0.998	0.581	3.86125668353186e-245	7	Acta2
Myocd	9.88260067764561e-204	0.870346110195976	0.371	0.045	5.61134066476718e-199	7	Myocd
Myl61	3.411978935748e-192	1.59464391690241	0.998	0.94	1.93732163971772e-187	7	Myl6
Mylk1	2.53137138826597e-186	2.11737822190885	0.963	0.673	1.43731267425742e-181	7	Mylk
Fhl1	5.48645137097855e-170	0.987996620333976	0.357	0.051	3.11520708844162e-165	7	Fhl1
Tpm23	8.05360449269458e-150	1.73144405326885	0.937	0.63	4.57283663095198e-145	7	Tpm2
Myl92	2.65804041795314e-130	1.56904154847651	0.926	0.634	1.50923534931379e-125	7	Myl9
Nexn	1.30508538528919e-123	1.20225559093356	0.571	0.173	7.41027481767202e-119	7	Nexn
Pcdh71	1.33034710595572e-107	1.21493019789471	0.55	0.18	7.55371086761655e-103	7	Pcdh7
Ramp11	8.32750102418701e-106	0.920784139086967	0.306	0.057	4.72835508153338e-101	7	Ramp1
Ppp1r12b	1.35926494058001e-93	1.1088666676732	0.566	0.208	7.71790633261331e-89	7	Ppp1r12b
Map1b1	5.58682907287868e-91	1.41483734662994	0.603	0.241	3.17220154758051e-86	7	Map1b
Tspan181	9.59774155499412e-90	0.743210446729954	0.318	0.069	5.44959765492566e-85	7	Tspan18
Lmcd11	3.58109577052425e-87	1.19710274325268	0.587	0.23	2.03334617850367e-82	7	Lmcd1
Foxp2	7.56421598146384e-73	0.709305490319347	0.276	0.061	4.29496183427517e-68	7	Foxp2
Syt11	2.73051154421371e-71	1.12087355188383	0.506	0.188	1.55038445480454e-66	7	Syt1
Ppp1r12a	3.98654168122268e-71	1.07585333090015	0.759	0.475	2.26355836659824e-66	7	Ppp1r12a
Ahnak21	3.19226684948623e-69	0.66458717184548	0.299	0.075	1.81256911713828e-64	7	Ahnak2
Cnn21	1.19355643708859e-68	0.978732865647865	0.763	0.459	6.77701344978904e-64	7	Cnn2
Plpp31	1.4911141862116e-68	1.40578134614638	0.787	0.526	8.46654634930945e-64	7	Plpp3
Flna1	3.39540483963554e-68	1.06089815593784	0.849	0.611	1.92791086794506e-63	7	Flna
Pdgfrl2	6.23970638178632e-67	1.10660273831442	0.508	0.203	3.54290528357827e-62	7	Pdgfrl
Carmn	1.2981129908459e-66	0.870620797130773	0.334	0.095	7.37068556202301e-62	7	Carmn
Itga12	1.33321767977513e-55	0.862629201686064	0.448	0.176	7.57000998576316e-51	7	Itga1
Cald11	6.32886128856679e-55	0.765716988827077	0.965	0.893	3.59352743964822e-50	7	Cald1
Vldlr	4.57423131320319e-53	0.762966136667434	0.381	0.137	2.59724853963677e-48	7	Vldlr
Actb1	8.06018876609801e-53	0.591873031463188	0.998	0.985	4.57657518139045e-48	7	Actb
Pls3	8.6757197674123e-53	0.930243998418505	0.622	0.369	4.9260736839367e-48	7	Pls3
Id4	2.97683893501386e-51	0.823365191036678	0.309	0.098	1.69024914730087e-46	7	Id4
Dstn	1.8325390665269e-50	0.739103488597346	0.97	0.897	1.04051568197397e-45	7	Dstn
Lpp	1.22755035177695e-49	0.925578472326888	0.708	0.479	6.97003089738955e-45	7	Lpp
Ncam12	5.90889243741048e-47	1.0324895658619	0.643	0.377	3.35506912596167e-42	7	Ncam1
Pcdh17	8.27677451120185e-46	1.00174607862694	0.285	0.093	4.69955256746041e-41	7	Pcdh17
Csrp22	4.34533258752065e-44	0.975604281510377	0.673	0.427	2.46727984319422e-39	7	Csrp2
Id21	5.20201542186447e-44	1.01743987106407	0.787	0.578	2.95370435653465e-39	7	Id2
Gsn1	3.67493388375589e-43	0.860069097433818	0.777	0.569	2.0866274591966e-38	7	Gsn
Slmap	1.19651187375935e-42	0.826514403453166	0.51	0.267	6.79379441920558e-38	7	Slmap
Fstl12	1.04456815159097e-41	0.709976470974246	0.893	0.755	5.93105796473354e-37	7	Fstl1
Tpm11	1.49885692813819e-41	0.614189770804527	0.991	0.93	8.51050963796864e-37	7	Tpm1
Actn1	6.64197907962427e-38	0.70151627051371	0.675	0.462	3.77131572141066e-33	7	Actn1
Csrp1	1.90979168336595e-36	0.664089325893147	0.831	0.637	1.08437971781518e-31	7	Csrp1
Mfap21	2.39121404117512e-36	0.805957543444959	0.856	0.781	1.35773133257923e-31	7	Mfap2
Dst1	7.56731431799313e-36	0.780144251518364	0.652	0.417	4.2967210697565e-31	7	Dst

Supplementary Table S3

	p_val	avg_log2FC	pct.1	pct.2	p_val_adj	cluster	gene
Col18a1	0	3.47023734269354	0.901	0.234	0	0	Col18a1
Gstm6	0	2.99169073299541	0.816	0.157	0	0	Gstm6
Pcsk6	0	3.10965330076666	0.884	0.25	0	0	Pcsk6
Atp5o	0	-2.249014321	0.167	0.798	0	0	Atp5o
Atp5md	0	-1.808532519	0.164	0.792	0	0	Atp5md
Usmg5	0	2.749756906	0.763	0.143	0	0	Usmg5
Aes	0	2.86226684104692	0.758	0.139	0	0	Aes
2410015M20Rik	0	2.54069937412901	0.755	0.138	0	0	2410015M20Rik
Micos10	0	-2.330856352	0.154	0.764	0	0	Micos10
Gatm	0	2.68868023515785	0.893	0.285	0	0	Gatm
Nme2	0	-2.561259892	0.262	0.85	0	0	Nme2
Micos13	0	-2.059094237	0.153	0.741	0	0	Micos13
Fabp5	0	-3.902215877	0.11	0.697	0	0	Fabp5
Atp5o.1	0	2.17109588142871	0.735	0.149	0	0	Atp5o.1
Csrp2	0	-3.290944662	0.186	0.765	0	0	Csrp2
Wnt6	0	2.40598106250038	0.824	0.254	0	0	Wnt6
Fbln1	0	-3.504042761	0.094	0.663	0	0	Fbln1
Itga6	0	3.08688737121597	0.942	0.383	0	0	Itga6
Rnasek	0	-2.504606446	0.133	0.683	0	0	Rnasek
Rbis	0	-1.673300868	0.15	0.7	0	0	Rbis
Peg3	0	2.65321235810427	0.882	0.337	0	0	Peg3
Minos1	0	2.12769340649381	0.674	0.139	0	0	Minos1
Aard	0	2.99028401686339	0.816	0.281	0	0	Aard
Timm23	0	-1.978969532	0.154	0.683	0	0	Timm23
Apoa4	0	-4.790776945	0.034	0.561	0	0	Apoa4
Lgals7	0	1.93847476510742	0.769	0.244	0	0	Lgals7
Car14	0	-5.556399593	0.014	0.533	0	0	Car14
Hsd17b1	0	-3.723256185	0.152	0.665	0	0	Hsd17b1
Ifitm1	0	3.22574246888707	0.768	0.263	0	0	Ifitm1
Ar	0	-2.42559429	0.196	0.7	0	0	Ar
mt-Nd4l	0	-2.247758573	0.343	0.847	0	0	mt-Nd4l
mt-Rnr1	0	2.24610535132202	0.624	0.121	0	0	mt-Rnr1
mt-Rnr2	0	2.46349124831372	0.625	0.122	0	0	mt-Rnr2
Mrpl53	0	-2.835366372	0.099	0.601	0	0	Mrpl53
Tomm6	0	-1.756362533	0.225	0.723	0	0	Tomm6
Myl9	0	2.73168635031754	0.784	0.292	0	0	Myl9
Tle5	0	-1.052546251	0.165	0.657	0	0	Tle5
Smad3	0	3.2618827609761	0.703	0.212	0	0	Smad3
Smc4	0	-2.946452065	0.193	0.677	0	0	Smc4
Znrd2	0	-2.294005779	0.108	0.586	0	0	Znrd2
Crispld2	0	-3.314812145	0.056	0.52	0	0	Crispld2
Gm47283	0	-1.84783708	0.247	0.711	0	0	Gm47283
AY036118	0	-1.421032618	0.224	0.687	0	0	AY036118
Nap1l5	0	-5.348967068	0.04	0.498	0	0	Nap1l5
Colec12	0	2.7475208719409	0.657	0.199	0	0	Colec12
mt-Co2	0	-1.072269996	0.431	0.888	0	0	mt-Co2
Atp5g2	0	-1.674749845	0.415	0.868	0	0	Atp5g2
Hpcal1	0	2.51447954433162	0.814	0.362	0	0	Hpcal1
Tox2	0	-4.286976638	0.032	0.481	0	0	Tox2

Cnmd	0	-2.935674898	0.092	0.54	0	0	Cnmd
Apoa4.1	0	2.08845740577407	0.807	0.266	0	1	Apoa4
Car14.1	0	1.92288364895444	0.756	0.249	0	1	Car14
Col18a1.1	0	-5.817650379	0.054	0.561	0	1	Col18a1
Pcsk6.1	0	-5.360505138	0.072	0.563	0	1	Pcsk6
Wnt6.1	0	-5.258449849	0.065	0.545	0	1	Wnt6
Hsd17b1.1	0	1.61687400707477	0.869	0.391	0	1	Hsd17b1
Fabp5.1	0	1.63547175196685	0.875	0.403	0	1	Fabp5
Gatm.1	0	-5.377996779	0.114	0.585	0	1	Gatm
Nap1l5.1	0	2.09741560818006	0.713	0.242	0	1	Nap1l5
Peg3.1	0	-5.256646534	0.148	0.619	0	1	Peg3
Grb14.1	0	2.64486671600793	0.916	0.451	0	1	Grb14
Prss23.1	0	2.45356440341462	0.826	0.363	0	1	Prss23
Atp5o.2	0	0.838672518032244	0.956	0.493	0	1	Atp5o
Atp5md.1	0	0.886375727623082	0.948	0.489	0	1	Atp5md
Csrp2.1	0	1.91012965373169	0.932	0.478	0	1	Csrp2
Sema5a.1	0	2.08947470494306	0.854	0.402	0	1	Sema5a
Usmg5.1	0	-11.36418913	0	0.439	0	1	Usmg5
Foxo1.1	0	2.05479953553485	0.957	0.519	0	1	Foxo1
Hs6st2.1	0	-4.567146352	0.074	0.509	0	1	Hs6st2
Gstm6.1	0	-6.677086184	0.028	0.463	0	1	Gstm6
Aes.1	0	-14.43810838	0	0.433	0	1	Aes
Atp5o.1.1	0	-10.09730259	0	0.433	0	1	Atp5o.1
2410015M20Rik.1	0	-12.02437512	0	0.43	0	1	2410015M20Rik
Hpgd.1	0	1.79237963685018	0.75	0.322	0	1	Hpgd
Nme2.1	0	1.09830380483035	0.994	0.567	0	1	Nme2
Myl9.1	0	-4.678292818	0.122	0.546	0	1	Myl9
Fxyd6.1	0	-3.792128316	0.173	0.596	0	1	Fxyd6
Micos13.1	0	0.906118916590212	0.88	0.459	0	1	Micos13
Micos10.1	0	0.731587410432391	0.895	0.476	0	1	Micos10
Rasl10a.1	0	2.15957279283299	0.613	0.198	0	1	Rasl10a
Bmpr1b.1	0	1.96181410500856	0.672	0.258	0	1	Bmpr1b
Itga6.1	0	-3.813055762	0.242	0.654	0	1	Itga6
Cnmd.1	0	1.65330256092928	0.713	0.302	0	1	Cnmd
Rbis.1	0	0.890171428049922	0.838	0.434	0	1	Rbis
Mpped2.1	0	1.83691892466718	0.636	0.233	0	1	Mpped2
Slc38a5.1	0	1.73340475315741	0.617	0.215	0	1	Slc38a5
Minos1.1	0	-11.90908094	0	0.399	0	1	Minos1
Ifitm1.1	0	-4.597127803	0.116	0.514	0	1	Ifitm1
Ank3.1	0	1.69520409199124	0.726	0.33	0	1	Ank3
Lgals7.1	0	-4.352867734	0.107	0.499	0	1	Lgals7
Plod2.1	0	1.46704631125482	0.887	0.495	0	1	Plod2
Pdpn.1	0	-3.456002308	0.126	0.512	0	1	Pdpn
mt-Nd4l.1	0	1.12479246884641	0.985	0.599	0	1	mt-Nd4l
Nr5a2.1	0	1.23289178351658	0.922	0.543	0	1	Nr5a2
Foxp2.1	0	2.31439478593972	0.575	0.196	0	1	Foxp2
Rnasek.1	0	0.781923037971146	0.802	0.424	0	1	Rnasek
Tox2.1	0	1.64771878016391	0.63	0.252	0	1	Tox2
Hes1.1	0	-3.351574483	0.235	0.612	0	1	Hes1
Esr2.1	0	1.52003287435943	0.702	0.326	0	1	Esr2
Cables1.1	0	1.62122398508225	0.734	0.359	0	1	Cables1

Cdca8.2	0	2.29490925796854	0.914	0.211	0	2	Cdca8
Tpx2.2	0	2.37563019641949	0.873	0.174	0	2	Tpx2
Mki67.2	0	1.97626528196676	0.903	0.211	0	2	Mki67
Birc5.2	0	2.04819871371703	0.934	0.248	0	2	Birc5
Ccna2.2	0	2.24425584366151	0.809	0.135	0	2	Ccna2
Cenpf.2	0	2.10808599106296	0.9	0.227	0	2	Cenpf
Pbk.2	0	2.47681525234911	0.8	0.128	0	2	Pbk
Prc1.2	0	2.08011643672275	0.859	0.196	0	2	Prc1
Ube2c.2	0	3.04729341840726	0.911	0.25	0	2	Ube2c
Pclaf.2	0	1.4575400096689	0.901	0.242	0	2	Pclaf
Top2a.2	0	1.87913749993863	0.926	0.269	0	2	Top2a
Spc24.2	0	1.87904093850022	0.883	0.233	0	2	Spc24
Cdca3.2	0	2.07275766275147	0.81	0.165	0	2	Cdca3
Racgap1.2	0	2.01799583435565	0.803	0.158	0	2	Racgap1
Cdc20.2	0	2.56396526367029	0.799	0.161	0	2	Cdc20
Cdk1.2	0	2.13973014804535	0.866	0.23	0	2	Cdk1
Spc25.2	0	1.9172135267827	0.772	0.143	0	2	Spc25
Hmmr.2	0	2.32246421746471	0.733	0.105	0	2	Hmmr
Cenpe.2	0	2.03153031885973	0.79	0.166	0	2	Cenpe
Nusap1.2	0	1.66505821657038	0.746	0.133	0	2	Nusap1
Ccnb1.2	0	2.46949696681709	0.721	0.112	0	2	Ccnb1
Knl1.2	0	1.689168173	0.747	0.141	0	2	Knl1
Ccnb2.2	0	2.15498811835801	0.795	0.195	0	2	Ccnb2
Tacc3.2	0	1.96747320075839	0.752	0.153	0	2	Tacc3
Bub1.2	0	2.45608952872661	0.675	0.093	0	2	Bub1
Kif15.2	0	1.91040620590419	0.708	0.126	0	2	Kif15
Aurkb.2	0	2.24932794120213	0.681	0.1	0	2	Aurkb
Smc2.2	0	1.49021039845354	0.934	0.355	0	2	Smc2
Kif11.2	0	1.96199927444322	0.692	0.114	0	2	Kif11
Bub1b.2	0	2.18604924564924	0.696	0.123	0	2	Bub1b
Cenpa.2	0	2.09222657972639	0.792	0.22	0	2	Cenpa
Cenpw.1	0	1.74646431182456	0.893	0.322	0	2	Cenpw
Kif23.2	0	1.5296695954784	0.738	0.167	0	2	Kif23
Incenp.2	0	1.70754995088384	0.746	0.179	0	2	Incenp
Kif22.2	0	2.19681109410447	0.695	0.13	0	2	Kif22
Ckap2l.2	0	1.93738321358961	0.676	0.112	0	2	Ckap2l
Kif20b.2	0	1.96916426068904	0.713	0.151	0	2	Kif20b
Mis18bp1.2	0	1.93419192530944	0.676	0.119	0	2	Mis18bp1
Sgo2a.2	0	2.14313656272005	0.655	0.099	0	2	Sgo2a
Cenpm.2	0	1.4154140958065	0.793	0.239	0	2	Cenpm
Dbf4.2	0	1.68870753327382	0.764	0.22	0	2	Dbf4
Cenpp.1	0	1.86888387609498	0.722	0.182	0	2	Cenpp
Esco2.2	0	1.73582872423973	0.644	0.105	0	2	Esco2
Kif4.2	0	2.0302617584347	0.639	0.101	0	2	Kif4
Rfc4.1	0	1.56797053270392	0.816	0.28	0	2	Rfc4
Knstrn.2	0	2.12079270375368	0.675	0.14	0	2	Knstrn
Aspm.2	0	2.19085335601461	0.635	0.102	0	2	Aspm
Gmnn.2	0	1.55038584335797	0.81	0.277	0	2	Gmnn
Tyms.2	0	1.58943796409825	0.818	0.295	0	2	Tyms
Ncapg.2	0	2.2284624316509	0.614	0.092	0	2	Ncapg
Col1a1.3	0	6.04979037223329	0.972	0.133	0	3	Col1a1

Mfap4.3	0	5.9140599666878	0.897	0.076	0	3	Mfap4
Bgn.3	0	5.11991346274553	0.949	0.136	0	3	Bgn
Sparcl1.3	0	5.09936180029364	0.912	0.103	0	3	Sparcl1
Dcn.3	0	5.42262979894711	0.946	0.14	0	3	Dcn
Ptn.2	0	5.87793412534871	0.886	0.088	0	3	Ptn
Rbp1.3	0	4.5664942319607	0.954	0.188	0	3	Rbp1
Lsp1.2	0	5.86431540347146	0.85	0.103	0	3	Lsp1
Ltbp4.3	0	4.60831468235203	0.818	0.09	0	3	Ltbp4
Mmp2.3	0	4.75541692348092	0.817	0.09	0	3	Mmp2
Col6a2.2	0	3.96606489159985	0.904	0.179	0	3	Col6a2
Dlk1.3	0	6.47349641734203	0.769	0.048	0	3	Dlk1
Col1a2.3	0	4.85536051980279	0.987	0.274	0	3	Col1a2
Cdh11.3	0	5.24494825697791	0.761	0.052	0	3	Cdh11
Igfbp7.3	0	3.28002902432436	0.934	0.248	0	3	Igfbp7
Mdk.2	0	3.78917727115924	0.972	0.298	0	3	Mdk
Islr.3	0	4.95183782752711	0.701	0.047	0	3	Islr
Igfbp4.3	0	3.57875960817442	0.906	0.256	0	3	Igfbp4
Tubb6.3	0	4.64420624800039	0.738	0.088	0	3	Tubb6
Sfrp1.3	0	4.8914882849544	0.744	0.095	0	3	Sfrp1
Emilin1.3	0	5.17566226195167	0.689	0.04	0	3	Emilin1
Tpm2.3	0	3.65548675117102	0.777	0.143	0	3	Tpm2
Mki67.3	0	3.12220458853572	0.891	0.26	0	3	Mki67
Fstl1.3	0	2.92703138348868	0.899	0.27	0	3	Fstl1
Col6a3.3	0	4.00621567558974	0.741	0.124	0	3	Col6a3
Inha.3	0	-2.423009598	0.286	0.9	0	3	Inha
Nusap1.3	0	3.46128853455819	0.786	0.172	0	3	Nusap1
Grb10.3	0	4.16604102109534	0.696	0.084	0	3	Grb10
Col6a1.2	0	3.07194329318432	0.941	0.329	0	3	Col6a1
Pclaf.3	0	3.36697060805575	0.896	0.289	0	3	Pclaf
Prc1.3	0	2.89835264085243	0.849	0.243	0	3	Prc1
Birc5.3	0	2.77162154909114	0.903	0.298	0	3	Birc5
Top2a.3	0	3.22596981843462	0.919	0.315	0	3	Top2a
Ncam1.3	0	3.1614292944263	0.741	0.138	0	3	Ncam1
Pmp22.3	0	3.46555779592628	0.791	0.189	0	3	Pmp22
Eln.3	0	4.29117908478834	0.664	0.064	0	3	Eln
Loxl1.3	0	4.40099819822849	0.704	0.106	0	3	Loxl1
Nid1.3	0	2.69391870293551	0.812	0.219	0	3	Nid1
Knl1.3	0	3.31336627910937	0.768	0.181	0	3	Knl1
Gpc3.3	0	3.36006902559509	0.718	0.131	0	3	Gpc3
Fst.3	0	-2.554971322	0.233	0.817	0	3	Fst
Cpe.2	0	-2.439380045	0.35	0.93	0	3	Cpe
Pdgfra.3	0	4.75935867357501	0.637	0.057	0	3	Pdgfra
Col3a1.2	0	3.70442932527249	0.986	0.414	0	3	Col3a1
Csrp1.2	0	3.76527172103999	0.733	0.161	0	3	Csrp1
Tagln2.3	0	3.01378577598965	0.812	0.244	0	3	Tagln2
Tpx2.3	0	2.5707126051403	0.796	0.229	0	3	Tpx2
Cdca8.3	0	2.46423278911452	0.833	0.267	0	3	Cdca8
Col5a2.3	0	3.29374867660838	0.725	0.162	0	3	Col5a2
Spc25.3	0	3.11911922308078	0.752	0.189	0	3	Spc25
Amh.3	0	1.98580660425588	0.947	0.391	0	4	Amh
Slc18a2.4	0	1.4231867639422	0.944	0.482	0	4	Slc18a2

Hsd11b2.3	0	1.83439780487685	0.634	0.264	0	4	Hsd11b2
Smim41.4	0	2.05463456025148	0.383	0.089	0	4	Smim41
Shbg.4	0	2.34016162884608	0.359	0.075	0	4	Shbg
Sox18.2	0	2.45651657247125	0.329	0.071	0	4	Sox18
mt-Co2.4	0	0.765424275347958	0.985	0.736	0	4	mt-Co2
Isyna1.3	0	0.938111478410011	0.974	0.785	0	4	Isyna1
Rpl9.3	0	0.423545763959566	1	0.999	0	4	Rpl9
Rps11.4	0	0.455452479115958	1	0.999	0	4	Rps11
Hspe1.3	1.96781815583414e-296	0.6939772514695	0.993	0.947	1.11732714888262e-291	4	Hspe1
Rpl30.4	1.74588198468242e-289	0.544076663585391	1	0.997	9.91311790902678e-285	4	Rpl30
Gdpd2.2	2.85051647691173e-282	1.87137886767014	0.415	0.122	1.61852325559048e-277	4	Gdpd2
Rps16.3	3.35831105726912e-266	0.359936113097128	1	0.999	1.90684901831741e-261	4	Rps16
Cd320.4	1.36631651041214e-263	1.07790330804104	0.877	0.571	7.75794514612012e-259	4	Cd320
Tmem184a.3	5.81673535372228e-256	1.13774568552662	0.684	0.304	3.30274233384351e-251	4	Tmem184a
Gm14226.3	1.95834069937758e-254	1.25774323849767	0.73	0.364	1.11194584910659e-249	4	Gm14226
Hsp90ab1.1	4.86700150329698e-249	0.338163753577222	1	0.999	2.76348345357202e-244	4	Hsp90ab1
Rpsa.3	1.87425623272982e-248	0.439854539206627	1	0.997	1.06420268894399e-243	4	Rpsa
Rps27a.2	4.00331415500742e-248	0.373626922169366	1	0.999	2.27308177721322e-243	4	Rps27a
Rpl24.4	1.66688640196961e-244	0.595723596397912	0.998	0.946	9.46458099038345e-240	4	Rpl24
Hspd1.3	5.10078505824013e-244	0.665007861090297	0.992	0.902	2.89622575606875e-239	4	Hspd1
mt-Co1.3	2.39036216873537e-242	0.55348319582859	1	0.997	1.35724763940794e-237	4	mt-Co1
Rps28.2	1.47638849989157e-241	0.438405210257932	1	0.999	8.38293390238435e-237	4	Rps28
Tle5.3	3.62047993288531e-239	0.780843518677577	0.872	0.485	2.05570850589228e-234	4	Tle5
Wnt4.4	5.71812188221652e-238	1.14069951544995	0.739	0.389	3.24674960472254e-233	4	Wnt4
Rps13.4	2.61391478649103e-237	0.387301449575614	1	0.998	1.4841808157696e-232	4	Rps13
Rps5.3	8.15211727748149e-236	0.393772732989026	1	0.999	4.62877219015399e-231	4	Rps5
Il13ra2.1	2.25564259831985e-232	1.97165585019851	0.272	0.064	1.28075386732601e-227	4	Il13ra2
Rps10.4	6.17981698637407e-231	0.603696110265134	0.996	0.957	3.5089000848632e-226	4	Rps10
Rps20.2	1.00899498396717e-230	0.382562259291024	1	1	5.72907351896558e-226	4	Rps20
Stra6.3	3.61141515367349e-229	1.46082250801895	0.466	0.167	2.05056152425581e-224	4	Stra6
Slc25a42.2	2.29602278321141e-226	1.68013859637132	0.371	0.115	1.30368173630744e-221	4	Slc25a42
Atp5o.5	2.90917975491866e-224	0.806405171025805	0.933	0.588	1.65183226484282e-219	4	Atp5o
Rpl3.3	4.77636456783541e-224	0.391127220964499	1	0.997	2.71201980161695e-219	4	Rpl3
Rpl27a.4	1.95435814133744e-219	0.557984771285162	0.999	0.99	1.1096845526514e-214	4	Rpl27a
Rpl17.3	2.56439793067956e-219	0.404742525623011	0.999	0.998	1.45606514503985e-214	4	Rpl17
Rpl13.4	1.31666793593026e-218	0.425200689412036	1	1	7.476040540212e-214	4	Rpl13
Rps19.4	4.05976707513431e-218	0.465578922908766	1	0.999	2.30513574526126e-213	4	Rps19
Rpl29.4	4.82773093708617e-218	0.490317429701487	0.998	0.986	2.74118562607753e-213	4	Rpl29
Gldn.4	2.36394287790141e-217	1.58309851905433	0.31	0.084	1.34224676607242e-212	4	Gldn
Rpl32.2	2.19783644615276e-216	0.327591006955184	1	1	1.24793153412554e-211	4	Rpl32
Rps2.3	3.14952474623878e-216	0.400220600184615	1	0.998	1.78830015091438e-211	4	Rps2
Eef1b2.3	3.70780364510078e-216	0.449951860392638	1	0.987	2.10529090968822e-211	4	Eef1b2
Socs2.4	2.50281802464204e-215	0.707342287444039	0.977	0.801	1.42110007439175e-210	4	Socs2
Cald1.3	6.85928551721687e-213	-2.861994893	0.301	0.594	3.89470231667574e-208	4	Cald1
Rpl28.3	1.44202967465158e-212	0.399771064499085	1	0.999	8.18784449267168e-208	4	Rpl28
Tppp3.4	3.23977110811739e-211	2.03794125803101	0.305	0.087	1.83954203518906e-206	4	Tppp3
Apoa1.4	4.05723136945251e-211	0.847141141725626	0.789	0.413	2.30369597157514e-206	4	Apoa1
Nnat.4	1.5355612756747e-208	1.18970632457473	0.733	0.408	8.71891692328094e-204	4	Nnat
Gng13.3	0	5.36604935443905	0.906	0.107	0	5	Gng13
Serpine2.4	0	-4.97214597	0.143	0.89	0	5	Serpine2
Aldh1a2.5	0	3.3484091728936	0.868	0.155	0	5	Aldh1a2

Prcd.5	0	3.67706430627784	0.897	0.219	0	5	Prcd
Aldh1b1.5	0	3.2570681158717	0.906	0.23	0	5	Aldh1b1
H19.5	0	3.3332496320085	0.861	0.189	0	5	H19
2410015M20Rik.4	0	2.18233684770402	0.947	0.288	0	5	2410015M20Rik
Gstm6.5	0	2.18875736325456	0.974	0.32	0	5	Gstm6
Nr5a2.3	0	-6.104621844	0.021	0.672	0	5	Nr5a2
Krt18.4	0	4.10757304354987	0.877	0.232	0	5	Krt18
Aes.4	0	1.92421375215902	0.93	0.29	0	5	Aes
Tenm4.4	0	-3.49546272	0.199	0.818	0	5	Tenm4
Atp5o.6	0	-4.503482919	0.028	0.642	0	5	Atp5o
Sulf2.4	0	-3.826674345	0.094	0.705	0	5	Sulf2
Atp5md.5	0	-4.142862354	0.029	0.637	0	5	Atp5md
Htra1.4	0	-3.725512075	0.202	0.809	0	5	Htra1
Mest.5	0	3.09415670410888	0.764	0.164	0	5	Mest
Usmg5.5	0	1.42085898703386	0.893	0.297	0	5	Usmg5
Fam129a.5	0	3.46693231397457	0.701	0.112	0	5	Fam129a
Lgals7.5	0	2.94569722431401	0.951	0.37	0	5	Lgals7
Minos1.5	0	1.81274476233118	0.849	0.269	0	5	Minos1
U90926.1	0	4.96335989302001	0.624	0.045	0	5	U90926
Atp5o.1.5	0	1.35992860311696	0.868	0.294	0	5	Atp5o.1
Wnt6.4	0	1.96348034165212	0.966	0.394	0	5	Wnt6
Nme2.5	0	-4.49467392	0.134	0.705	0	5	Nme2
Gm45889.5	0	2.77435612472201	0.795	0.23	0	5	Gm45889
Gja1.5	0	-3.928635239	0.257	0.82	0	5	Gja1
Ano1.4	0	2.82741683933978	0.767	0.205	0	5	Ano1
Irx3.4	0	2.62088864634183	0.855	0.293	0	5	Irx3
Mfap2.3	0	-3.00167808	0.234	0.79	0	5	Mfap2
Tnni1.5	0	4.23785583698719	0.63	0.082	0	5	Tnni1
mt-Rnr2.4	0	2.42584690439565	0.79	0.243	0	5	mt-Rnr2
mt-Rnr1.5	0	2.42007700698644	0.789	0.243	0	5	mt-Rnr1
Ephx2.2	0	-2.945452724	0.209	0.754	0	5	Ephx2
Gatm.5	0	2.04318894005821	0.981	0.438	0	5	Gatm
Hs6st2.4	0	2.24177726290467	0.906	0.371	0	5	Hs6st2
Smpx.5	0	3.1957007651277	0.644	0.115	0	5	Smpx
Celf2.5	0	2.74209033833603	0.747	0.223	0	5	Celf2
Apoc1.3	0	4.3933755669457	0.593	0.069	0	5	Apoc1
St3gal5.3	0	-2.482664331	0.282	0.805	0	5	St3gal5
Spats2l.5	0	2.56488974524576	0.756	0.236	0	5	Spats2l
Cgn.4	0	2.64560294714264	0.726	0.207	0	5	Cgn
Lgr5	0	5.96631646543066	0.534	0.019	0	5	Lgr5
Espn.4	0	2.83304531130256	0.731	0.221	0	5	Espn
Krt8.3	0	3.05040956658417	0.871	0.372	0	5	Krt8
Peg3.4	0	1.77929096260547	0.971	0.473	0	5	Peg3
Atp5g2.5	0	-2.504130439	0.26	0.758	0	5	Atp5g2
Tspan18.5	0	2.54874819032846	0.736	0.24	0	5	Tspan18
Cox7a1.3	0	3.85106237463338	0.613	0.119	0	5	Cox7a1
Adora1.2	0	3.81036310750473	0.557	0.065	0	5	Adora1
Mrap.6	0	5.70637837922795	0.97	0.114	0	6	Mrap
Tinagl1.6	0	6.16952605157562	0.914	0.066	0	6	Tinagl1
Cyp11a1.5	0	4.55973132663896	0.988	0.203	0	6	Cyp11a1
Cemip	0	6.51713531634628	0.812	0.03	0	6	Cemip

Prlr.6	0	4.29562519945222	0.956	0.182	0	6	Prlr
Runx2	0	6.66181531418852	0.785	0.025	0	6	Runx2
Slc25a30.5	0	4.6288941344632	0.919	0.174	0	6	Slc25a30
Prss35.6	0	4.60557614155571	0.977	0.247	0	6	Prss35
Sbsn.4	0	4.64684834268753	0.879	0.158	0	6	Sbsn
Lhcgr.6	0	4.15052828226852	0.79	0.073	0	6	Lhcgr
Star.5	0	3.91451036996205	0.808	0.112	0	6	Star
Mgarp.6	0	4.6478819042726	0.987	0.292	0	6	Mgarp
Lgals3.1	0	4.78521023728319	0.746	0.055	0	6	Lgals3
Ppp1r14a.5	0	4.72037696834944	0.792	0.104	0	6	Ppp1r14a
S100a6.3	0	5.07390624576517	0.984	0.297	0	6	S100a6
Rora.6	0	4.35851365163951	0.844	0.165	0	6	Rora
Mgst2.4	0	4.31976572694756	0.757	0.079	0	6	Mgst2
Cited4.4	0	3.65755463648252	0.904	0.233	0	6	Cited4
Sfrp4.4	0	4.52718714535354	0.755	0.096	0	6	Sfrp4
Cdkn1a.5	0	4.20984515059953	0.839	0.18	0	6	Cdkn1a
Glul.5	0	4.09359220710285	0.93	0.286	0	6	Glul
Acsl4.5	0	3.86696960341312	0.81	0.172	0	6	Acsl4
Mt2.3	0	3.49846097641595	0.895	0.267	0	6	Mt2
Heg1.4	0	3.53914828796218	0.955	0.331	0	6	Heg1
Loxl2.5	0	4.09390070082743	0.761	0.139	0	6	Loxl2
Gm2a.4	0	3.82917991418904	0.96	0.339	0	6	Gm2a
Parm1.5	0	3.1353130537037	0.801	0.189	0	6	Parm1
Avpi1.4	0	3.24515185542765	0.804	0.2	0	6	Avpi1
Tagln2.6	0	2.45984388890725	0.854	0.264	0	6	Tagln2
Dhcr7.5	0	3.47850965007787	0.743	0.158	0	6	Dhcr7
Kcnk3	0	4.67804939255324	0.623	0.045	0	6	Kcnk3
Ltbp1.4	0	2.80565325269128	0.861	0.29	0	6	Ltbp1
Fabp4	0	6.48145605799208	0.592	0.026	0	6	Fabp4
Rnaseh2a.5	0	3.49397989161112	0.95	0.387	0	6	Rnaseh2a
Entpd1.3	0	3.53600696936961	0.67	0.109	0	6	Entpd1
Jakmip3	0	4.80470398175873	0.597	0.036	0	6	Jakmip3
Chst11.3	0	2.82698101323129	0.794	0.234	0	6	Chst11
Aif1l.5	0	3.07344014896047	0.831	0.271	0	6	Aif1l
Susd3.4	0	3.13365539293034	0.928	0.374	0	6	Susd3
Tnfrsf12a.5	0	2.25332671893646	0.878	0.324	0	6	Tnfrsf12a
Sgk1.2	0	3.48099387568942	0.905	0.357	0	6	Sgk1
Hs3st1.6	0	2.40479492666474	0.829	0.281	0	6	Hs3st1
Neat1.4	0	3.59365967620905	0.951	0.403	0	6	Neat1
Frmd5.4	0	3.23889787828742	0.678	0.132	0	6	Frmd5
Ermp1.4	0	2.90154741323385	0.753	0.211	0	6	Ermp1
Tmem86a.5	0	2.99977167157798	0.982	0.441	0	6	Tmem86a
Acsbg1.5	0	2.58981327812997	0.964	0.423	0	6	Acsbg1
Abcb1b	0	3.97338127020535	0.57	0.031	0	6	Abcb1b
Id2.6	0	4.0826151878261	0.968	0.429	0	6	Id2
Bnip3.5	0	3.31696474996565	0.9	0.365	0	6	Bnip3
Fxyd1.7	0	4.2235580849052	0.868	0.125	0	7	Fxyd1
Cyp11a1.6	0	3.97002206175901	0.915	0.21	0	7	Cyp11a1
Smoc2.6	0	4.94600885941684	0.833	0.141	0	7	Smoc2
Prss35.7	0	2.73468646078067	0.937	0.253	0	7	Prss35
Prlr.7	0	3.06818984312282	0.863	0.19	0	7	Prlr

Igfbp7.7	0	3.40384020294404	0.939	0.278	0	7	Igfbp7
Prxl2a.6	0	4.58063576069633	0.778	0.154	0	7	Prxl2a
Cped1.7	0	2.74226375319932	0.829	0.219	0	7	Cped1
Lhcgr.7	0	3.47958607190757	0.684	0.081	0	7	Lhcgr
Mgarp.7	0	2.61992352497316	0.883	0.3	0	7	Mgarp
Osr2.4	0	3.65916532132862	0.691	0.127	0	7	Osr2
Aebp1.7	0	3.09089058948919	0.968	0.42	0	7	Aebp1
Hao2.7	0	4.19316584791261	0.636	0.099	0	7	Hao2
Acsbg1.6	0	2.90936366218906	0.959	0.426	0	7	Acsbg1
Pcolce.5	0	2.38508320368356	0.939	0.419	0	7	Pcolce
Ppp1r14a.6	0	2.61071806466532	0.626	0.114	0	7	Ppp1r14a
Ephx1.7	0	4.03403583472121	0.596	0.086	0	7	Ephx1
Mt2.4	0	2.77025091722146	0.776	0.275	0	7	Mt2
Cav1.5	0	3.15774751897029	0.606	0.107	0	7	Cav1
Map1b.6	0	-3.136528086	0.377	0.869	0	7	Map1b
Cd47.5	0	2.12737358385788	0.812	0.325	0	7	Cd47
Rbp1.7	0	1.57586357182565	0.717	0.231	0	7	Rbp1
Cyp17a1	0	8.24232177483542	0.522	0.037	0	7	Cyp17a1
Star.6	0	3.46587929241998	0.609	0.124	0	7	Star
E330017L17Rik.5	0	3.20031763937775	0.606	0.123	0	7	E330017L17Rik
Mrap.7	0	1.92364366269323	0.612	0.133	0	7	Mrap
Tle5.6	0	1.86758057429475	0.967	0.494	0	7	Tle5
Dtx4	0	4.22729604698499	0.516	0.044	0	7	Dtx4
Me1.7	0	2.63170439705587	0.934	0.464	0	7	Me1
Fdxr.5	0	2.30048933499845	0.925	0.462	0	7	Fdxr
Ctsf.4	0	2.42205586454201	0.704	0.244	0	7	Ctsf
Abca1.6	0	2.94960514002251	0.705	0.248	0	7	Abca1
Tpm2.7	0	2.16363717773007	0.632	0.176	0	7	Tpm2
Nrp2.2	0	3.54590830066466	0.521	0.072	0	7	Nrp2
Aldh1a1.7	0	2.689291803	0.969	0.521	0	7	Aldh1a1
Pkdcc.4	0	3.559218214	0.536	0.089	0	7	Pkdcc
Rerg.4	0	3.31790450538564	0.521	0.076	0	7	Rerg
Gsta2	0	5.61107044128565	0.464	0.02	0	7	Gsta2
Twist1.1	0	3.32673394067344	0.486	0.048	0	7	Twist1
1110008P14Rik.5	0	2.89804629746185	0.697	0.259	0	7	1110008P14Rik
Tmem86a.6	0	2.03354860040507	0.879	0.448	0	7	Tmem86a
Serpine2.6	0	-3.231426177	0.448	0.871	0	7	Serpine2
Ogn.2	0	3.6010905267257	0.489	0.066	0	7	Ogn
Tulp2.4	0	2.8583799894686	0.5	0.078	0	7	Tulp2
Tmem159.6	0	2.55660457623649	0.581	0.16	0	7	Tmem159
Sct	0	5.59916919151861	0.451	0.032	0	7	Sct
Rnasek.6	0	1.89335069300268	0.913	0.506	0	7	Rnasek
Bicc1.7	0	2.35167444561949	0.506	0.102	0	7	Bicc1
Plin1	0	6.29166380862483	0.41	0.008	0	7	Plin1
Slc7a8.5	0	3.08366735806908	0.492	0.093	0	7	Slc7a8
Gm42418.7	4.73390508359417e-183	1.87003708443334	0.989	0.993	2.68791130646477e-178	8	Gm42418
AY036118.7	1.01787760076389e-150	3.04571797385017	0.786	0.546	5.77950901713734e-146	8	AY036118
Sdc4.7	5.11157113803126e-126	-2.232545439	0.133	0.76	2.90235009217415e-121	8	Sdc4
Gstm2.8	7.92101887381536e-120	-2.716678507	0.231	0.817	4.49755451655236e-115	8	Gstm2
Maged2.7	1.65622892776132e-117	-2.38947236	0.231	0.835	9.4040678518288e-113	8	Maged2
Gstm1.7	3.70183150086786e-116	-2.328475441	0.225	0.819	2.10189992619277e-111	8	Gstm1

Anxa2.8	7.6153938657717e-115	-2.260376668	0.138	0.721	4.32402063698517e-110	8	Anxa2
Marcksl1.7	2.25546682390212e-114	-1.337652912	0.199	0.855	1.28065406261163e-109	8	Marcksl1
Acaa2.7	2.17750711154711e-110	-1.744966914	0.125	0.726	1.23638853793645e-105	8	Acaa2
Med10.7	3.93458754172225e-108	-1.369134296	0.17	0.797	2.23405880618989e-103	8	Med10
Cirbp.8	1.25349328498834e-102	-1.18602544	0.138	0.742	7.11733487216377e-98	8	Cirbp
Hsp90ab1.4	3.67856833763328e-102	-0.590769995	0.963	0.999	2.08869110210817e-97	8	Hsp90ab1
Bmyc.7	3.34702120307625e-100	-1.761246253	0.131	0.701	1.90043863910669e-95	8	Bmyc
Hmgcs2.7	3.7347005565951e-100	-1.948513962	0.105	0.663	2.1205629760347e-95	8	Hmgcs2
Cdkn1b.7	6.53041967605671e-98	-2.054591115	0.103	0.648	3.707972292065e-93	8	Cdkn1b
Tmem176b.7	2.49211664280219e-97	-1.174762182	0.21	0.818	1.41502382978308e-92	8	Tmem176b
Cdc42ep5.7	1.07710481036642e-96	-1.570822716	0.155	0.723	6.11580111326052e-92	8	Cdc42ep5
Eid1.5	4.89535517986213e-94	-0.896770056	0.144	0.741	2.77958267112572e-89	8	Eid1
Kdm5b.6	2.93144406751524e-93	-1.588268299	0.083	0.62	1.66447394153515e-88	8	Kdm5b
Dctn2.4	1.49298563101419e-92	-1.102110089	0.127	0.706	8.47717241289855e-88	8	Dctn2
Tsc22d1.6	3.1927825124854e-92	-1.512865878	0.518	0.964	1.81286191058921e-87	8	Tsc22d1
Capns1.6	5.63229095163674e-92	-1.139419877	0.194	0.792	3.19801480233934e-87	8	Capns1
Ech1.6	1.11180021866455e-91	-0.817285932	0.175	0.793	6.31280164157732e-87	8	Ech1
Gnai2.6	8.3607602788047e-91	-0.7617419	0.227	0.864	4.74723968630531e-86	8	Gnai2
Gstm5.8	9.49453136446074e-91	-1.081640995	0.138	0.715	5.39099490874081e-86	8	Gstm5
Ldhb.8	6.12623846741563e-90	-1.179817881	0.426	0.949	3.47847820179859e-85	8	Ldhb
Ppdpf.5	2.50830438696165e-88	-0.813682337	0.125	0.691	1.42421523091682e-83	8	Ppdpf
Akr1a1.7	2.56666770313594e-88	-1.012547307	0.315	0.927	1.45735392184059e-83	8	Akr1a1
Srp14.6	2.73519493249341e-88	-0.780515819	0.208	0.827	1.55304368266976e-83	8	Srp14
Sqstm1.7	1.73124217639284e-87	-0.99893686	0.172	0.753	9.82999307755855e-83	8	Sqstm1
Tmem176a.7	1.16068836573718e-85	-0.981168844	0.148	0.703	6.59038854065571e-81	8	Tmem176a
Polr2g.5	2.86126876859496e-85	-0.79813845	0.135	0.696	1.62462840680822e-80	8	Polr2g
App.6	5.2997010465174e-85	-1.26300497	0.161	0.706	3.00917025421258e-80	8	App
Cpe.6	3.35980915469763e-84	-1.120546883	0.336	0.894	1.90769963803732e-79	8	Cpe
1110065P20Rik.8	4.26339130545894e-84	-1.099258889	0.118	0.654	2.42075358323959e-79	8	1110065P20Rik
Supt4a.2	1.39480276912022e-83	-0.584831551	0.159	0.738	7.91969012306459e-79	8	Supt4a
Ube2r2	1.74722011820918e-83	-0.830471461	0.161	0.736	9.9207158311917e-79	8	Ube2r2
S100a10.8	3.87106118584041e-83	-1.26364142	0.304	0.841	2.19798854132019e-78	8	S100a10
Itm2c.8	5.38830574889303e-83	-1.039354558	0.153	0.703	3.05948000422146e-78	8	Itm2c
Fam104a.4	5.65656221497194e-83	-0.554689755	0.175	0.764	3.21179602566107e-78	8	Fam104a
Tst.7	7.00676363872854e-83	-1.149013002	0.124	0.656	3.97844039407006e-78	8	Tst
Ttc3.6	5.34087019000484e-82	-0.747305768	0.229	0.831	3.03254609388475e-77	8	Ttc3
Ube2e3.8	6.67533337113384e-82	-0.757639251	0.142	0.695	3.79025448129794e-77	8	Ube2e3
Spint2.7	2.20677202597411e-81	-1.599609399	0.072	0.56	1.2530051563481e-76	8	Spint2
Nfe2l2.5	3.95731334657293e-81	-1.043271323	0.146	0.689	2.24696251818411e-76	8	Nfe2l2
Idh1.8	7.89103047005990e-81	-1.378547135	0.144	0.67	4.48052710090001e-76	8	Idh1
Atxn7l3b.7	8.69512042631702e-81	-0.807201913	0.301	0.91	4.9370893780628e-76	8	Atxn7l3b
Mxd4.7	1.54392790743513e-80	-1.678403028	0.066	0.546	8.76642265841668e-76	8	Mxd4
Aamp.6	2.11509298870093e-80	-0.75304336	0.181	0.761	1.20094979898439e-75	8	Aamp
Map1lc3a.7	2.34098133672666e-80	-0.839273504	0.229	0.825	1.3292092029934e-75	8	Map1lc3a

Supplementary Table S4. Information of antibody, primer sequences, and reagents

a. Antibody information

Protein name	Manufacture (catalogue number)	Applications (working dilution)
Nucleolin	abcam(ab129200)	IHC(1:1000)
Lrp1	abcam(ab92544)	IHC(1:1000)
Aidh1a2	abcam (ab75674)	IHC(1:2000)
Cyp17a1	Proteintech(14447-1-AP)	IHC(1:1000)
Enpep-PE	Santa Cruz Biotechnology(sc-52444)	FCA(1 μg per 1x10^6 cells)
Enpep	Proteintech(17655-1-AP)	IF(1:200)
Collegan IV	abcam(ab309503)	IF(1:100)
Lama1	abcam(ab314883)	IF(1:100)
α-SMA	Abcam(ab124964)	IF(1:1000)

b. Primer sequences

Gene name	Sequences (5’-3’)	Application
Mdk	5'>AATTAACCCTCACTAAAGGGATGCAGCACCGAGGCTTCTT<3'	ISH
	5'>TAATACGACTCACTATAGGGTTAGTCCTTTCCTTTTCCTTTCTTG<3'	ISH
Enpep	5'>AATTAACCCTCACTAAAGGGATGAACTTTGCAGAGGAAGAGC<3'	ISH
	5'>TAATACGACTCACTATAGGGTCTGCAGCCTGGATCACCAC<3'	ISH
Ptn	5'>AATTAACCCTCACTAAAGGGATGAATGTATGTGCGTTCTCTCT<3'	ISH
	5'>TAATACGACTCACTATAGGGTTAGTTAACGGTGATTTCATTTGCTAC<3'	ISH
Ncl	5'>AATTAACCCTCACTAAAGGGATGGTGAAGCTCGCAAAGG<3'	ISH
	5'>TAATACGACTCACTATAGGGCATCCTCTGAGGCAGGAGCA<3'	ISH
Lrp1	5'>AATTAACCCTCACTAAAGGGACTATGGATGCCCCTAAAACTTG<3'	ISH
	5'>TAATACGACTCACTATAGGGATCTACTGGCTCATTCTTGGC<3'	ISH
MDK	5’>AATTAACCCTCACTAAAGGGGATTGCGGCGTGGGTTTC<3’	ISH
	5’>TAATACGACTCACTATAGGGTGTGGGGAAGAACAAAAGCG<3’	ISH

Foxl2	5'>ACAACACCGGAGAAACCAGAC<3'	q-PCR
	5'>CGTAGAACGGGAACTTGGCTA<3'	
Amhr2	5'>GGGGCTTTGGACACTGCTT<3'	q-PCR
	5'>GTCTCGGCATCCTTGCATCTC<3'	
Gapdh	5'>AGGTCGGTGTGAACGGATTTG<3'	q-PCR
	5'>TGTAGACCATGTAGTTGAGGTCA<3'	
Cyp17a1	5'>GCCCAAGTCAAAGACACCTAAT<3'	q-PCR
	5'>GTACCCAGGCGAAGAGAATAGA<3'	
Star	5'>ATGTTCCTCGCTACGTTCAAG<3'	q-PCR
	5'>CCCAGTGCTCTCCAGTTGAG<3'	
Ptn	5'>ATGTCGTCCCAGCAATATCAGC<3'	q-PCR
	5'>CCAAGATGAAAATCAATGCCAGG<3'	
Mdk	5'>GAAGAAGGCGCGGTACAATG<3'	q-PCR
	5'>GAGGTGCAGGGCTTAGTCA<3'	
Col1a1	5'>GCTCCTCTTAGGGGCCACT<3'	q-PCR
	5'>GCTCCTCTTAGGGGCCACT<3'	
Enpep	5'>ATAGTGGGACTTTCTGTGGGT<3'	q-PCR
	5'>GGTCGTAGTGAACTGGATTGATG<3'	
Fshr	5'>TGTGTAACCTCGCCTTTGCT<3'	q-PCR
	5'>AAATTGGGGCCATGCAGAGA<3'	
Lhcgr	5'>ACGAGACGCTTCATCACTCTG<3'	q-PCR
	5'>GGGAGGCAGATGCTGACTTT<3'	
Cyp11a1	5'>ACTTCCGGTTACAGGATCGC<3'	q-PCR
	5'>CCAAAGCCCTTCCAAAACCG<3'	
Cyp19a1	5'>GCTGTAGGGGGCATACACAT<3'	q-PCR
	5'>CACAGTTTCCGGGACCATGA<3'	
Hsd17b1	5'>GACCGTTCCCAGAGCTTCAA<3'	q-PCR
	5'>GGCCAGCATTACAGACCAGT<3'	

c. Reagents

Reagent	Source	Identifier
DAB peroxidase substrate kit	Zsbio	ZLI-9018
SlowFade® Gold Antifade Reagent	Beyotime	P0126-25ml
Chromogenic solution	Biyotime Biotechnology	C3206
Trypsin/EDTA	Sigma	T8003
Type I collagenase	Worthington	LS004197
MEM Alpha(1X)	Gibco	32561-037
Recombinant Human Follitropin Alfa Solution for Injection(FSH)	Merck Serono	928002
Bovine Serum Albumin(BSA)	Sigma	A1933-25G
Fetuin	Sigma	F3004-1G
ITS	Sigma	13146-5ML
Midkine human	Sigma	SRP3114

TUNEL BrightRed Apoptosis Detection Kit	Vazyme	A113-01
Four-color multiple fluorescent immunohistochemical staining kit	Absin	abs50012
Single cell sequence specific amplification kit	Vazyme	P621
